# CVD-grown tunable carbon films for high-performance sodium storage

**DOI:** 10.1039/d6ee01852a

**Published:** 2026-06-12

**Authors:** Enis Oğuzhan Eren, Evgeny Senokos, Tim Horner, Leonardo Cancellara, Ernesto Scoppola, Jiyong Kim, Morten Johansen, Kangkang Ge, Barbara Daffos, Pierre-Louis Taberna, Patrice Simon, Paolo Giusto

**Affiliations:** a Department of Colloid Chemistry, Max Planck Institute of Colloids and Interfaces 14476 Potsdam Germany enis.eren@mpikg.mpg.de paolo.giusto@mpikg.mpg.de; b Department of Biomaterials, Max Planck Institute of Colloids and Interfaces 14476 Potsdam Germany; c Functional Materials and Devices, Fraunhofer Institute for Applied Polymer Research IAP 14476 Potsdam Germany; d Deutsches Elektronen-Synchrotron DESY Notkestr. 85 22607 Hamburg Germany; e Université de Toulouse, CIRIMAT UMR CNRS 5085 118 Route de Narbonne 31062 Toulouse France; f Réseau sur le Stockage Electrochimique de l’Energie (RS2E) Rue Beaudelocque 80000 Amiens France

## Abstract

Sodium-ion batteries are considered a promising and sustainable energy-storage technology, yet achieving competitive energy density requires electrode materials with high reversible capacity. Here, we introduce a chemical vapor deposition strategy that allows the growth of uniform, electronically continuous carbon coatings onto highly porous carbon substrates for use as high-capacity negative electrodes. This coating narrows and partially seals the open pore network, suppresses surface reactivity, and establishes nanoconfined domains that allow sodium to be stored in a more compact manner. This restructuring significantly enhances sodium storage performance, delivering total reversible capacities exceeding 500 mAh g^−1^ together with an unprecedented 420 mAh g^−1^ reversible plateau capacity. By enabling control over heteroatom composition, confinement, and electronic structure, this approach not only addresses the challenge of designing uniform and electronically continuous carbon coatings on highly porous substrates but also establishes a general route for designing functional carbon films for advanced electrodes, catalysts, and interfaces with tunable electronic properties.

## Abstract 2

A thin tunable carbon film grown by CVD confines porous carbons, enabling diffusion-controlled Na-ion storage with ultrahigh plateau capacity for sustainable sodium-ion batteries.

## Graphical abstract

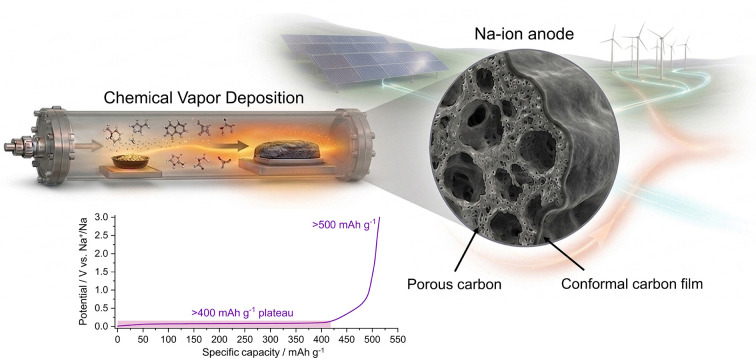

**Broader context**

The transition toward sustainable energy systems requires the development of cost-effective, high-performance, and resource-abundant battery technologies beyond lithium. Sodium-ion batteries are particularly attractive due to the natural abundance of sodium, yet their widespread adoption is hindered by the lack of suitable electrode materials capable of delivering high performance. Disordered carbons have emerged as promising anode materials. However, conventional synthesis approaches often provide limited control over pore architecture, surface chemistry, and interfacial properties, making it challenging to systematically tailor electrochemical performance while simultaneously maintaining scalable material processing. Such a reaction-limited chemical vapor deposition strategy provides a versatile route to engineer conformal and electronically tunable carbon films within porous carbon systems. Beyond achieving exceptional reversible capacities, it also enables additive- and current collector-free electrode architectures, thereby simplifying electrode fabrication. More broadly, such carbon films emerge as a promising platform for tailoring interfacial chemistry, transport properties, and functional behavior across a wide range of electrochemical and electronic applications.

Carbons with open and accessible porosity are widely used in electrochemical energy storage technologies, benefiting from rapid ion transport and large surface areas.^1–3^ Despite these advantages, most of them remain fundamentally incompatible with the requirements of sodium-ion batteries (SIBs), a technology gaining momentum as a sustainable complement to lithium-ion batteries (LIBs).^4–7^ The highly open structure promotes surface-driven storage and defect-associated binding, which drive capacitive behavior, excessive solid–electrolyte interphase formation, and severe initial capacity loss.

Electrochemical sodium storage in carbonaceous materials becomes advantageous only when sodium forms quasimetallic clusters within nanoconfined spaces, a behavior that requires closed or partially closed pore environments, such as those found in hard carbons.^8–11^ The formation of these clusters generates a distinct voltage plateau, which is critical for achieving high energy density at the application scale.^12–15^ However, hard carbon synthesis relies on precursor-driven thermal condensation, which offers limited ability to rationally control closed porosity. As a result, progress has largely relied on time- and resource-intensive empirical precursor screening rather than rational structural design.^16,17^

A more versatile approach is to start from abundant, structurally well-understood commercial porous carbons and restructure them into sodium-compatible systems. Establishing such a strategy would unlock a tunable platform for anode design beyond conventional hard carbons. Here, carbon films offer a promising route for such structural transformation.^18–21^ Their intrinsic physicochemical properties enable them to stabilize reactive surfaces and narrow or partially close porous networks. However, realizing uniform and well-controlled carbon films on highly porous substrates remains a challenge. Vapor-phase deposition and infiltration methods offer controllable routes, but the growth often becomes limited by mass transport, resulting in uneven thickness and conformal growth.^22,23^

Here, we developed a reaction-limited atmospheric pressure chemical vapor deposition (AP-CVD) method that addresses this limitation, converting highly porous carbons into nano-structurally confined, electronically integrated materials through conformal carbon thin films (Cfilms). When applied to commercial activated carbon fibers (ACF), the coating forms a continuous layer that tightens the pore network, mitigates surface reactivity, and gives rise to the confined spaces required for efficient sodium storage. As a result, these Cfilm/ACF hybrids deliver exceptional performance, reaching a total reversible capacity of 515 mAh g^−1^, of which 420 mAh g^−1^ arises from the low-voltage plateau region, among the highest reported for carbonaceous anodes. The chemical tunability of the precursors further allows systematic modulation of heteroatom incorporation, structural order, and electronic properties, establishing a general design platform for electrochemical, catalytic, and electronic applications.

## Reaction-limited growth of tunable carbon films

**Fig. 1:**
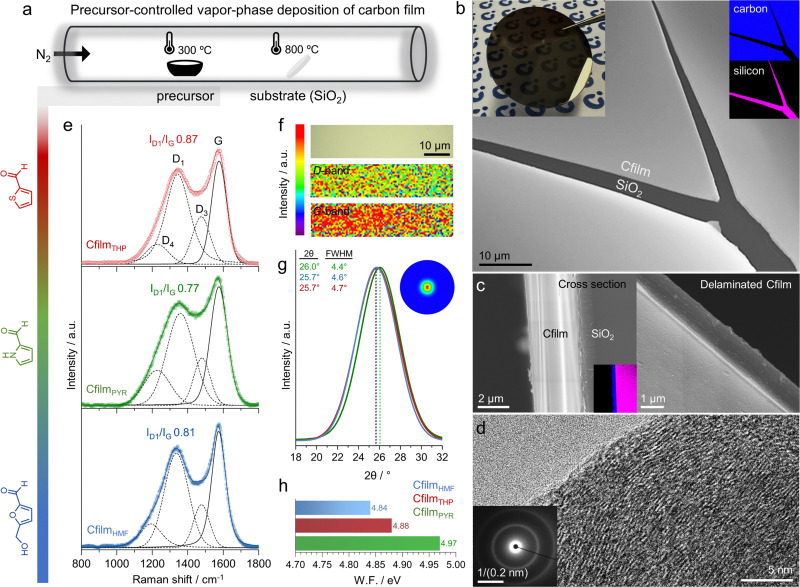
Vapor-phase deposition of carbon films from heterocyclic precursors and their structural properties. (a) Schematic of the CVD setup used to deposit carbon films on a SiO_2_ wafer. Heterocyclic precursors, thiophene-2-carbaldehyde (top, red), 1*H*-pyrrole-2-carbaldehyde (middle, green), and 5-(hydroxymethyl)furan-2-carbaldehyde (HMF) (bottom, blue), are vaporized at 300 °C and deposited on a substrate at 800 °C under a nitrogen atmosphere. (b) Optical image of a wafer-scale carbon film (inset) and SEM image of the carbon film deposited on a SiO_2_ substrate featuring an on-purpose notch to enhance elemental contrast. Insets: EDS elemental maps showing distinct distributions of carbon and silicon. (c) SEM images of a cross-section and a delaminated edge of the carbon film, highlighting its uniform thickness and conformity to the substrate. Inset: EDS mapping of the cross-section highlighting the separation between carbon and silicon regions. (d) High-resolution TEM image of the carbon film, revealing a layered, turbostratic structure. Inset: Corresponding SAED pattern. (e) Raman spectra of the carbon films derived from the three precursors, displaying characteristic D and G bands with multi-peak fitting. (f) Raman mapping of the D- and G-band intensities across the film surface, indicating lateral homogeneity. (g) GIXRD patterns of the carbon films, showing C(002) peaks associated with the graphitic stacking. Inset: Pole figure of the C(002) reflection. (h) Work function values of the films measured by PESA, highlighting differences in electronic properties as a function of precursor identity.

Carbon films were deposited from heterocyclic aldehyde precursors (Fig. 1a), where vaporized molecules undergo surface-mediated condensation under reaction-limited conditions to form smooth, continuous films. The presence of an aldehyde group in the precursor is critical, as it enhances thermal condensation by promoting intermolecular crosslinking during vapor-phase growth, thereby enabling efficient formation of extended sp^2^-rich carbon networks. Beyond facilitating film formation, the incorporation of different heteroatoms allows systematic modulation of the electronic structure and surface polarity, influencing conductivity, defect chemistry, and interfacial interactions. Accordingly, thiophene-2-carbaldehyde, 1*H*-pyrrole-2-carbaldehyde, and 5-(hydroxymethyl)furan-2-carbaldehyde were used as sulfur-, nitrogen-, and oxygen(alone)-containing carbon sources, respectively, providing access to compositionally tunable carbon films (Cfilm_THP_, Cfilm_PYR_, and Cfilm_HMF_). As confirmed by thermogravimetric analysis coupled with mass spectrometry (TGA-MS, Note S1), these precursors decompose at relatively low temperatures to generate reactive gaseous intermediates, effectively promoting the formation of heteroatom-doped carbon films.

The carbon films can be deposited on a wide range of substrates by relying on appropriate interfacial interactions, enabling uniform coating on flat surfaces such as SiO_2_ and Cu-foil (Note S2), as well as on porous carbon powders. The resulting carbon films exhibit uniform coverage on SiO_2_ wafers (Fig. 1b, inset). Scanning electron microscopy (SEM) reveals smooth, defect-free surfaces at the microscale, while an intentional scratch highlights the clear compositional contrast between the carbon layer and the SiO_2_ wafer, as shown by energy-dispersive X-ray spectroscopy (EDS) (Fig. 1b, inset and Note S3). Cross-sectional SEM imaging (Fig. 1c, left panel) shows a seamless interface between the carbon layer and the wafer, with no evidence of delamination or interfacial gaps. In the right panel (Fig. 1c), an intentionally delaminated region reveals that the film preserves its continuous form and remains intact after peeling it off.

Transmission electron microscopy (TEM) reveals a partially crystalline structure, characterized by short-range order and pseudo-graphitic layers (Fig. 1d). The corresponding selected area electron diffraction (SAED) pattern displays clear yet slightly diffuse rings, indicative of local graphitic alignment without long-range arrangements. The formation of these layers suggests a polymerization-condensation pathway that promotes structural alignment and graphitic organization. Consequently, the films do not display the amorphous-like texture of hard carbons or diamond-like carbons (DLC), but instead show local order and layered features characteristic of soft carbons.^24,25^ This behavior originates from the reaction-limited vapor-phase growth mechanism, where controlled precursor decomposition and surface-mediated growth enable the formation of a soft carbon structure with well-defined graphitic-like domains. Unlike conventional bulk thermal condensation, where, for example, the HMF precursor yields highly disordered hard carbon even at higher temperatures,^12^ the deposition of thin films from HMF by AP-CVD leads to carbon films with more extended graphitic-like domains at moderate temperatures, highlighting the critical role of the deposition and growth kinetics under reaction-limited conditions.

The Raman spectra (Fig. 1e and Note S4) corroborate the TEM findings, showing partial graphitic order with broad D and G bands that position the films between hard and graphitic carbons.^26–28^ The incorporation of nitrogen within the graphitic lattice, where substitutional nitrogen introduces comparatively less lattice distortion. In contrast, sulfur and oxygen functionalities are mainly located at defect sites or sheet edges, where they contribute more strongly to structural disorder. Raman mapping further confirms uniform D and G band distributions across the film surface (Fig. 1f). These structural features are further corroborated by grazing-incidence X-ray diffraction (GIXRD) analysis (Fig. 1g). The diffractograms reveal a C(002) reflection with a lower full width at half maximum (FWHM) than the typical hard carbons.^24^ A slight shift in the peak position among the films reflects differences in stacking distance. Cfilm_PYR_ shows a marginally smaller *d*_002_ compared with Cfilm_HMF_ and Cfilm_THP_, as discussed in Note S4. The corresponding pole figure exhibits a uniform ring pattern, confirming the absence of preferred orientation and supporting the isotropically stacked nature of the turbostratic domains.

The work functions of the carbon films, crucial for governing charge transfer and interfacial reactions, were evaluated by photoelectron spectroscopy in air (PESA, Note S4) (Fig. 1h). The measured values are 4.84 eV for Cfilm_HMF_, 4.88 eV for Cfilm_THP_, and 4.97 eV for Cfilm_PYR_, showing that the precursor chemistry influences the surface electronic properties. The higher work function of the nitrogen-derived Cfilm_PYR_ reflects the typical character of N-doped noble carbons,^29^ featuring a lower Fermi level and improved stability against oxidation. This indicates an enhanced ability to accommodate additional electrons and resistance to undesirable surface reactions.^30^ In contrast, sulfur and oxygen functionalities in Cfilm_THP_ and Cfilm_HMF_ slightly lower the work function owing to their electron-donating nature.^31^ Sheet resistance measurements (Table S1) show that Cfilm_PYR_ exhibits the lowest resistance among the films, followed by Cfilm_HMF_ and Cfilm_THP_, confirming that the precursor chemistry directly modulates the electrical conductivity.

## Carbon film growth on activated carbon fibers

**Fig. 2:**
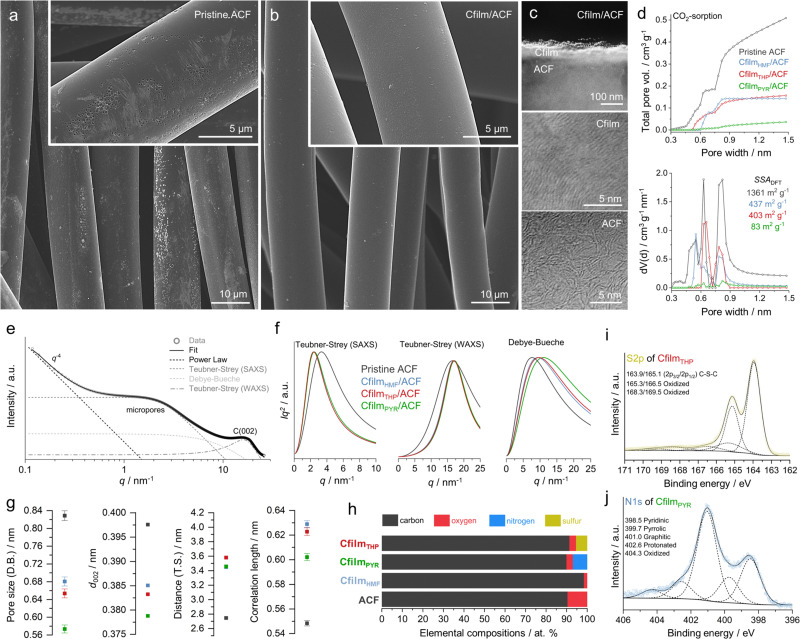
Physicochemical characterizations of pristine ACF and Cfilm/ACF hybrids. SEM images of pristine (a) ACF and (b) Cfilm/ACF. Insets show higher magnification images highlighting surface texture. (c) HRTEM images of the Cfilm/ACF interface, prepared as a cross-sectional slice using the ultramicrotome method. The top dark-field image shows a denser carbon film deposited uniformly on the ACF surface. The middle and bottom images reveal the structural contrast of the Cfilm and underlying ACF, respectively. (d) Cumulative pore volumes (top) and pore size distributions (bottom) of pristine ACF and Cfilm/ACF hybrids, derived from CO_2_ physisorption measurement. (e) Representative SAXS profile of Cfilm/ACF fitted using a combination of functions. (f) Kratky plots of Teubner–Strey and Debye–Bueche fits to SAXS and WAXS patterns, revealing macro- and nano-scale structural changes induced by Cfilm. (g) Quantitative analysis of structural parameters extracted, including correlation length, domain distance (Teubner–Strey), pore size (Debye–Bueche), and interplanar distance of C(002). (h) Elemental compositions of ACF and different Cfilm/ACF samples obtained by XPS. XPS spectra of (i) S2p of Cfilm_THP_ showing thiophene and oxidized sulfur species; (j) N1s of Cfilm_PYR_ indicating the presence of pyridinic, pyrrolic, graphitic, and oxidized nitrogen species.

The coating of porous carbons requires a reaction-limited and uniform deposition across high-surface-area and tortuous pore networks. To test this stringent condition, activated carbon fibers (ACF) with high surface area (>1500 m^2^ g^−1^) were chosen as a representative porous substrate. Pristine ACF exhibit a rough, heterogeneous surface with defective and irregular morphology at the SEM scale (Fig. 2a). After the Cfilm deposition, these surface irregularities are fully masked, and the Cfilm/ACF hybrids feature a smooth, continuous coating that uniformly follows the curvature of each individual fiber, free of cracks or particulate residues (Fig. 2b), as further detailed in Note S5. TEM images of ultramicrotomed cross-sections reveal a continuous Cfilm layer tightly adhered to the ACF scaffold (Fig. 2c). The interface between the two regions is well-defined with a pronounced contrast, arising from the density difference, separating the Cfilm from highly disordered ACF.

To assess the effect of Cfilm on the gas-accessible surface area, complementary N_2_ and CO_2_ sorption measurements were conducted (Fig. 2d and Note S6). Pristine ACF exhibits a high specific surface area (SSA_BET_ = 1630 m^2^ g^−1^) due to its highly open micro- and meso-porous network. After the Cfilm deposition, N_2_ uptake is nearly suppressed, indicating that the carbon layer effectively seals the pore structure and prevents molecular access to internal pores. The CO_2_ sorption further confirms this trend: the CO_2_-accessible surface area (*SSA*_DFT_) decreases from 1361 m^2^ g^-1^ in pristine ACF to 437, 403, and 83 m^2^ g^-1^ for Cfilm_HMF_/ACF, Cfilm_THP_/ACF, and Cfilm_PYR_/ACF, respectively (Fig. 2d). The Cfilm_PYR_ coating imposes the strongest confinement, likely due to smaller, more reactive gas-phase species infiltrating and condensing within the porous network.

Structural rearrangements within the Cfilm/ACF hybrids were further examined by small- and wide-angle X-ray scattering (SAXS/WAXS, Note S7). The scattering profiles were modeled using a combination of Debye-Bueche and Teubner–Strey functions to describe the hierarchical and partially ordered nature of the Cfilm/ACF hybrids (Fig. 2e and f).^32^ This approach allows quantitative evaluation of pore correlations, domain spacing, and correlation length, providing structural information independent of gas accessibility (Fig. 2g). The average pore size decreases from 0.83 nm in pristine ACF to 0.57–0.68 nm after Cfilm deposition, indicating that the Cfilm not only conforms to the external pore entrances but also tightens internal pores through partial filling and interfacial densification. Simultaneously, the domain distance expands from 2.7 to 3.6 nm, and the correlation length increases from 0.55 to 0.63 nm, reflecting the emergence of more coherent Cfilm domains surrounding the ACF scaffold. In the WAXS region, the C(002) reflection indicates a decrease in the average interlayer spacing, representing the combined scattering response of the ACF scaffold and the Cfilm.

The surface chemistry of the Cfilms and pristine ACF was analyzed by X-ray photoelectron spectroscopy (XPS) to understand how the precursor chemistry influences the bonding configuration and elemental composition of the resulting films (Note S8). The quantified compositions show that Cfilm_PYR_ contains 6.9 at% N and Cfilm_THP_ contain 5.3 at% S, while Cfilm_HMF_ and pristine ACF display only oxygen functionalities. Pristine ACF exhibits a relatively high oxygen content of 9.5 at%, originating from functional groups inherited by the activation process, whereas Cfilm_HMF_ contains only 1.5 at % oxygen (Fig. 2h). The S2p spectrum of Cfilm_THP_ shows a dominant doublet at 163.9 and 165.1 eV, corresponding to C–S–C bonding of thiophene (Fig. 2i).^33,34^ Such thiophenic sulfur cannot be incorporated into a graphitic carbon framework, as sulfur's larger covalent radius and preferred bonding configuration introduce significant local distortion and disrupt the sp^2^ carbon network. Instead, sulfur is stabilized at edge sites and structural defects, where the local curvature and lower coordination allow the formation of aromatic C–S rings. In contrast, the N1s spectrum of Cfilm_PYR_ is dominated by a peak of graphitic-N (401.0 eV), with weaker pyrrolic-N and pyridinic-N components (Fig. 2j).^35,36^ Unlike sulfur, nitrogen can replace a carbon atom in graphitic configuration while donating an electron pair into the π-system. The dominance of graphitic-N in Cfilm_PYR_ corresponds to its higher work function and suggests improved electronic delocalization and oxidative stability.^29^ Consistent with this, Cfilm_PYR_/ACF exhibits the highest electrical conductivity, followed by Cfilm_HMF_/ACF and Cfilm_THP_/ACF, which can be attributed to a longer π-conjugated electronic network arising from substitutional nitrogen incorporation that suppresses charge-carrier scattering within the graphitic lattice (Note S9).

### Carbon film-mediated transition to diffusion-controlled sodium storage

**Fig. 3:**
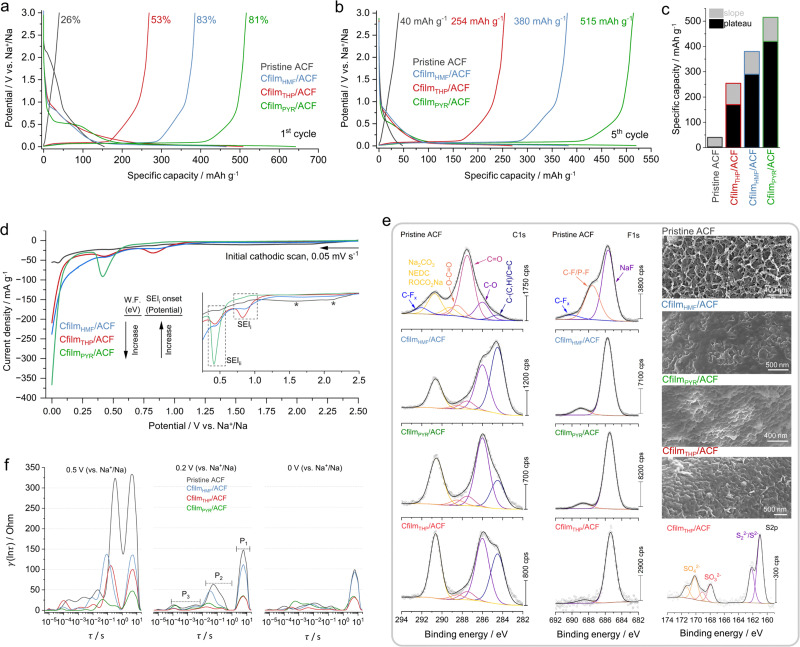
Electrochemical evaluation of pristine ACF and Cfilm/ACF electrodes. (a) Initial GCD cycles at a current density of 30 mA g^−1^. Numbers indicate the ICE for each sample. (b) Fifth GCD cycles illustrating improved reversible capacities for Cfilm/ACF. (c) Bar chart deconvoluting reversible capacities into slope- and plateau-type contributions, revealing an increased fraction of diffusion-limited storage. (d) Initial cathodic CV scan at 0.05 mV s^−1^, showing a correlation between the SEI onset potential and the work function of the carbon films. Inset: Zoom-in of SEI formation regions. (e) XPS depth profiling spectra collected after 2000 s Ar^+^ sputtering, together with SEM images of cycled electrodes after the initial cycle. The C1s and F1s spectra reveal pronounced differences in SEI composition between pristine ACF and Cfilm/ACF electrodes. The pristine ACF exhibits extensive fluorophosphate/organic fluorinated decomposition together with thick dendritic porous SEI structures, whereas the coated electrodes point to more chemically controlled interphases. Cfilm_THP_/ACF additionally shows sulfur-containing species, indicating active participation of sulfur functionalities in interphase formation. (f) DRT from impedance spectra measured during initial discharge at 0.5, 0.2, and 0 V (*vs.* Na^+^/Na).

The sodium storage behavior of the Cfilm/ACF electrodes was next evaluated in a half-cell configuration. The Coulombic efficiency (ICE) of the pristine ACF sample during the first galvanostatic charge–discharge (GCD) cycle (Fig. 3a) was only 26%, reflecting important irreversible reactions arising from the high surface area and reactivity of ACF, where abundant oxygen-containing functional groups and open porosity promote parasitic reactions and unstable SEI growth.^13,37^ Following Cfilm deposition, ICE values improved to 53%, 83%, and 81% for Cfilm_THP_/ACF, Cfilm_HMF_/ACF, and Cfilm_PYR_/ACF, respectively. The Cfilm effectively passivates the reactive ACF surface, closing accessible sites responsible for irreversible electrolyte decomposition and enabling more controlled SEI formation. Here, differences in ICE are not expected to be primarily governed by electrolyte-available surface, as all Cfilm/ACF samples exhibit negligible N_2_-accessible surface area (probing pore mouths >1 nm) that are readily available to solvated sodium ions, while smaller pores (<1 nm) detected by CO_2_ sorption experiments have restricted pore entrances and are only partially accessible (especially <0.5 nm),^13^ with a lower contribution to first-cycle irreversibility. Here, ICE values reaching up to 83% are comparable to those reported for carbon anodes with similar plateau capacities (Table S3). This reflects a certain trade-off, where the nanoconfined pore structures required to achieve extended low-voltage plateaus can also amplify irreversibility during the first cycle. In particular, insufficient desolvation may promote electrolyte decomposition within confined regions.^38^ Such effects may be mitigated, for example, by optimizing aging protocols.^38^ Further improvements in ICE can be achieved by reducing surface reactivity, optimizing pore accessibility, and through electrode and electrolyte formulation.^39,40^ The lower ICE of Cfilm_THP_/ACF is attributed to its higher content of sulfur- and oxygen-containing functional groups. Sulfur functionalities exhibit a strong affinity toward alkali metals, which can promote irreversible sodium trapping during the first cycle, while oxygen-containing groups further enhance electrolyte decomposition.^41–43^ In contrast, the Cfilm_PYR_/ACF sample, composed predominantly of graphitic nitrogen species, exhibits improved interfacial stability due to its more electronically stabilized surface, while Cfilm_HMF_/ACF shows the highest ICE, consistent with its lower content of oxygenated groups.

Cfilms not only improve the ICE but also enable significantly higher reversible capacity, reaching 255 (±9), 381 (±10), and 519 (±14) mAh g^−1^ for Cfilm_THP_/ACF, Cfilm_HMF_/ACF, and Cfilm_PYR_/ACF, respectively, compared with 40 mAh g^−1^ for pristine ACF (Fig. 3b and Note S17). Beyond the capacity enhancement, the emergence of a well-defined low voltage plateau is particularly significant, highlighting a transition from surface- to diffusion-controlled sodium storage mechanism, enhancing the energy density of the electrode.^8,44^ The *b*-value analysis (Note S10) further supports this interpretation, revealing a clear transition from surface-driven storage in pristine ACF to diffusion-controlled kinetics in the Cfilm/ACF electrodes. Capacity contributions show that the slope capacity remains nearly identical across all Cfilm/ACF samples, while the plateau contribution varies significantly with the nature of the Cfilms (Fig. 3c). This indicates that, unlike surface-driven sodium storage, the formation and stabilization of confined sodium clusters are substantially affected by the specific chemistry of coated carbon films. In particular, the Cfilm_PYR_/ACF electrode delivers a desodiation plateau capacity of 420 mAh g^−1^ (<0.15 V *vs.* Na^+^/Na, unless otherwise stated), which, to the best of our knowledge, exceeds previously reported values for carbon-based anodes (Table S3). Compared with Cfilm_THP_ and Cfilm_HMF_, the Cfilm_PYR_ layer induces a stronger structural compaction and acts as an electrically continuous overlayer that bridges defective regions, reconnecting otherwise isolated graphene fragments and restoring lateral electronic coherence across the fiber surface, as evidenced by the improved electrical conductivity (Note S9). This restored continuity facilitates the formation and stabilization of quasimetallic sodium clusters in nanoconfined regions.^9^ In contrast, Cfilm_THP_/ACF, though structurally similar to Cfilm_HMF_/ACF, delivers a lower plateau capacity. Both oxygen and sulfur do not directly participate in the graphitic structure and therefore reside at edge and defect sites or within surface functional groups. In the Cfilm_THP_, the much higher sulfur and oxygen content compared to the oxygen content in the Cfilm_HMF_ leads to a pronounced decrease in plateau capacity due to sodium trapping and restricted ion mobility. These interactions limit cluster growth and suppress the development of an extended plateau region.^34^

Cyclic voltammetry (CV) was then employed to investigate the SEI formation. The initial cathodic scan at 0.05 mV s^−1^ (Fig. 3d) reveals differences between pristine ACF and Cfilm/ACF samples. The cathodic features between 2.5 and 1.5 V (stars in the inset, Fig. 3d) observed for pristine ACF arise from parasitic reactions associated with its high-surface-area, defect-rich nature. In contrast, these features are largely suppressed in all Cfilm/ACF electrodes, confirming that the carbon film effectively passivates the surface and mitigates side reactions in this potential window, leading to more controlled SEI formation at lower potentials. At lower potentials, two cathodic features labeled SEI_i_ and SEI_ii_ can be distinguished, corresponding to a distinct interphase formation process governed by the surface properties of the deposited carbon film. Cfilm_PYR_/ACF exhibits a strongly suppressed SEI_i_ together with a pronounced SEI_ii_ peak, which could originate from its graphitic-N-rich and electronically stabilized surface that delays solvent reduction. In contrast, Cfilm_THP_/ACF shows both features, indicating more complex interfacial reactions and probably less controlled SEI evolution.

To gain further insights, *ex situ* SEM and XPS depth-profiling were conducted after the formation cycle. SEM images reveal that pristine ACF develops a thick branched dendritic-like interphase, whereas the Cfilm/ACF electrodes form comparatively smoother and more homogeneous SEI layers (Fig. 3e and Note S11). XPS depth profiling (Fig. 3e, after 2000 s sputtering, more details in Note S11) further confirms substantial differences in SEI chemistry. The C1s spectra consist of multiple contributions, including C–C/C=C (∼284.5 eV), together with oxidized carbon species assigned to C–O, C=O, and O–C=O at sequentially higher binding energies. Around ∼290 eV, strong contributions originate from organic and inorganic carbonate products commonly found in SEI, including Na_2_CO_3_, ROCO_2_Na, and sodium ethylene dicarbonate (NEDC).^45–47^

The pristine ACF electrode exhibits a substantially different interphase chemistry compared to the coated systems. In particular, the C1s spectra show a dominant C=O contribution rather than the C–O-dominated features observed for the Cfilm/ACF electrodes, together with pronounced C–F_*x*_ species and the absence of the underlying carbon signal even after prolonged sputtering. This behavior indicates the formation of a substantially thicker SEI, likely originating from extensive electrolyte decomposition on the highly oxygenated and defect-rich ACF surface. Consistently, the F1s spectra also reveal more pronounced C–F_*x*_ and C–F/P–F contributions.

For the Cfilm/ACF electrodes, the F1s and C1s spectra offer similar features (Fig. 3e), where NaF, together with organic/inorganic carbonate species, dominate the SEI composition. Among them, Cfilm_PYR_/ACF exhibits the strongest relative NaF contribution. Combined with the delayed emergence of the C–C/C=C signal during sputtering, this suggests the formation of a relatively thicker and NaF-rich interphase. This behavior may correlate with the extended SEI_ii_ feature around ∼0.5 V during the initial cathodic sweep, reflecting prolonged electrolyte decomposition. In Cfilm_THP_/ACF, sulfur-containing species are observed after cycling, indicating active participation of sulfur functionalities in interphase formation. Considering that sodium adsorption on sulfur-containing sites can occur at relatively higher potentials,^42^ irreversible interfacial redox reactions may occur during the initial cycle, leading to the formation of reduced sulfur species, potentially associated with Na–S bonding environments, together with oxygenated sulfur moieties incorporated into the SEI.^48^ Furthermore, the overall F1s intensity is significantly weaker, while the C1s spectra show a stronger Na_2_CO_3_ contribution, indicating a sulfur- and carbonate-rich SEI rather than a NaF-dominated interphase. Given the important role of NaF in forming mechanically robust and ionically conductive SEI,^49^ the reduced NaF fraction together with the emergence of sulfur moieties can explain the comparatively inferior electrochemical behavior of Cfilm_THP_/ACF.

*In situ* electrochemical impedance spectroscopy (EIS) combined with distribution of relaxation times (DRT) analysis was used to deconvolute the distinct electrochemical processes at different potentials, as detailed in Notes S12 and S13, respectively. As shown in Fig. 3f, three characteristic relaxation regions are identified, corresponding to solid-state sodium diffusion within the carbon matrix (P_1_, *τ* > ≈10^0^ s), charge-transfer resistance (P_2_, ≈10^−2^ < *τ* < ≈10^0^ s), and interfacial resistance associated with the passivation layer (P_3_, ≈10^−4^ < *τ* < ≈10^−2^ s).^50–52^ Compared to pristine ACF, all Cfilm/ACF electrodes exhibit reduced P_1_ and P_2_ contributions, indicating facilitated solid-state sodium diffusion and faster charge-transfer processes. At intermediate potentials (*e.g.*, 0.5 V), pristine ACF exhibits substantially larger solid-state diffusion and charge-transfer resistances than Cfilm/ACF samples (Fig. 3f). This behavior originates from its heterogeneous composition and surface morphology, which promotes nonuniform electrolyte decomposition and inefficient redox processes, hindering both charge transfer and diffusion.

At 0.2 V (Fig. 3f), corresponding to the onset of the plateau region, interfacial processes associated with SEI formation are largely established, and sodium transport proceeds through the formed interphase. In this potential range, the P_2_ contribution decreases and becomes negligible at lower potentials, reflecting reduced charge-transfer resistance under increasing overpotential. In contrast, the P_3_ contribution and diffusion-related features remain largely unchanged, demonstrating stable interphase and transport behavior.

Upon full sodiation at 0 V, a substantially larger P_1_ is observed for pristine ACF and Cfilm_HMF_/ACF, revealing stronger diffusion limitations. By comparison, Cfilm_PYR_/ACF and Cfilm_THP_/ACF exhibit a reduced P_1_ contribution, corresponding to lower effective diffusion resistance. This trend highlights the beneficial role of nitrogen- and sulfur-containing carbon films in establishing an improved ionic transport within the carbon framework.

**Fig. 4:**
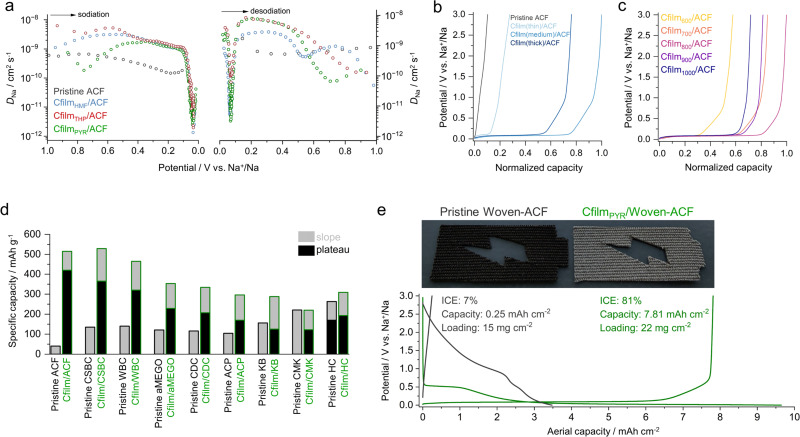
(a) Sodium ion diffusion coefficients during sodiation and desodiation obtained from GITT measurements. (b) Normalized capacity plot of Cfilm/ACF samples with varying film thicknesses, demonstrating that an optimal coating thickness is required to achieve high plateau capacities. (c) Normalized capacity as a function of deposition temperature, identifying an optimal deposition regime. (d) Comparison of slope and plateau capacities for pristine and Cfilm_PYR_-coated porous carbons, highlighting the systematic enhancement of sodium storage. (e) Optical images of freestanding Woven-ACF and Cfilm_PYR_/Woven-ACF. The coated sample exhibits a clear color change from black to grey, easily distinguishable by the naked eye. Areal capacity profiles of pristine Woven-ACF and Cfilm_PYR_/Woven-ACF, showing significantly improved ICE and high areal capacity.

Galvanostatic intermittent titration technique (GITT) was employed to probe the sodium-ion diffusion behavior of Cfilm/ACF electrodes (Note S14). As shown in Fig. 4a, all Cfilm/ACF samples exhibit a similar profile. In the plateau region, the relative diffusion coefficient (*D*_Na_) sharply decreases during sodiation due to charge repulsion as sodium accumulates near pore entrances.^8,11,44^ With further sodiation, *D*_Na_ rapidly recovers as pore filling progresses and quasimetallic sodium clustering occurs, where charge neutralization mitigates repulsion and facilitates faster ion transport.^8^ During sodiation, Cfilm_PYR_/ACF exhibits a shallow decrease in the sodium diffusion coefficient around 0.7 V, coinciding with a minor plateau feature in the GCD profile (Fig. 3b) and indicating a transient diffusion hindrance. Outside this region, all Cfilm/ACF electrodes maintain higher diffusion coefficients than pristine ACF. This trend is consistent with the DRT analysis, where pristine ACF shows a more pronounced solid-state diffusion impedance compared to the Cfilm/ACF electrodes. During desodiation, diffusion coefficients largely follow the same trends as sodiation (Fig. 4a), with Cfilm_HMF_/ACF and pristine ACF showing slightly lower values between 0.2 and 0.5 V due to higher interfacial and diffusion resistance. *Ex situ* pair distribution function (PDF) analyses were additionally performed (Note S14). Upon sodiation, attenuation of C–C correlations is observed, reflecting modification of the local carbon environments due to ionic Na–C interactions within the confined structure.^53^ Most of these features recover after desodiation, indicating reversible sodiation/desodiation processes. Importantly, no pronounced Na–Na correlations characteristic of sodium metal-like features are observed.

The influence of Cfilm thickness and deposition temperature on electrochemical performance was further examined to identify optimal coating conditions. Fig. 4b shows that the reversible capacity clearly depends on film thickness. Thinner films provide insufficient coverage, leaving portions of the ACF surface exposed to side reactions, whereas excessively thick films hinder ion transport by over-confining the porous network,^18^ as structurally confirmed by SAXS/WAXS analysis (Note S7). Similarly, the deposition temperature strongly affects the structural and electrochemical properties (Fig. 4c). Films deposited at 800 °C promote the largest plateau. Low temperatures (<800 °C) lead to limited thermal condensation, while high deposition temperatures (>800 °C) promote excessive graphitization that restricts sodium-ion mobility by limiting diffusion pathways and accessible sites, as detailed in Note S15. These findings highlight that both optimal film thickness and deposition temperature are essential to balance structural confinement.

Extending the Cfilm coating method to a range of porous carbon substrates (Note S16) results in similar enhancements in sodium storage performance. In several cases, reversible capacities exceeding 400 mAh g^−1^ are achieved, reaching 465 mAh g^−1^ for coated wood-based carbon (Cfilm_PYR_/WBC) and 528 mAh g^−1^ for coated coconut shell-based carbon (Cfilm_PYR_/CSBC). However, when considering plateau capacity alone, Cfilm_PYR_/ACF remains the top-performing porous carbon (Fig. 4d). The Cfilm approach efficiently suppresses electrolyte-accessible pores to levels comparable to commercial hard carbons, although the extent of sealing depends on the intrinsic pore architecture. For example, CO_2_ sorption indicates that the Cfilm_PYR_/CSBC system is nearly fully sealed, whereas Cfilm_PYR_/ACF retains higher ultramicroporous surface area. This highlights that while the Cfilm method effectively modulates pore accessibility, complete sealing is ultimately dictated by the host carbon structure. Consistent with this, highly open porous carbons benefit most from the coating method, whereas carbons with intrinsically closed pore structures (*i.e.*, hard carbons) exhibit a more limited change in the electrochemical performances, as discussed in Note S16.

Rate capability and cycling stability tests are thoroughly explained in Note S17. The rate performance is governed by both intrinsic structural properties and electrode-level parameters that control ion transport and storage kinetics. In particular, increasing the degree of structural ordering (*e.g.*, *via* higher deposition temperatures) leads to a denser carbon framework with reduced pore accessibility, which limits ion transport pathways and increases diffusion resistance at high current densities. Conversely, samples with lower degrees of graphitization exhibit a larger contribution from the sloping capacity region, associated with faster surface-driven processes, resulting in improved capacity retention at high rates. In addition, electrode design parameters such as mass loading play a critical role. Increasing areal loading leads to thicker electrodes with longer ionic diffusion distances and higher tortuosity, thereby enhancing transport limitations and polarization under high-rate conditions. Furthermore, the nature of the underlying carbon substrate significantly influences the rate performance. Substrates that favor a higher contribution from sloping capacity generally exhibit better rate capability, whereas those dominated by plateau capacity show reduced performance due to the diffusion-limited nature of sodium storage in nanoconfined pores.

When benchmarked against leading anode materials (Note S18), Cfilm_PYR_/ACF stands out by simultaneously delivering one of the highest plateau capacities and one of the lowest average operating voltages,^11,14,54^ a combination that is particularly impactful at the device level as it minimizes anode polarization and directly enhances the energy density of the full-cell. To demonstrate practical relevance, full-cell analysis was carried out using Cfilm_PYR_/ACF paired with an NVPF cathode, validating the electrode's compatibility in a realistic configuration (Note S19).

Beyond performance enhancement, this approach is inherently versatile and enables the direct deposition of functional carbon films onto woven, free-standing activated carbon fiber fabrics (Woven-ACF), eliminating the need for binders, conductive additives, and current collectors (Fig. 4e). Upon Cfilm_PYR_ coating, the woven electrodes show a boost in reversible capacity (0.25 *vs.* 7.81 mAh cm^−2^), the emergence of a well-defined plateau once again, and a significant improvement in ICE (7 *vs.* 81%) compared with pristine fabric (Fig. 4e). Such functional carbon textiles open new horizons for lightweight, mechanically flexible, and scalable electrode architectures, facilitating their integration into advanced sodium-ion batteries where high gravimetric efficiency, structural simplicity, and manufacturing compatibility are critical.

## Conclusion

We demonstrate a vapor-phase deposition strategy that enables conformal, composition-tunable carbon films capable of reshaping highly porous carbons into architectures that unlock sodium storage in otherwise inaccessible regimes. This approach provides a flexible route for surface engineering beyond the limitations of conventional hard-carbon synthesis. More generally, the ability to tailor heteroatom chemistry and interfacial reactivity through controlled vapor-phase growth opens opportunities for advanced electrochemical and catalytic interfaces. A key advantage of this method is its flexibility and mild reaction conditions: the reaction-limited process does not require vacuum conditions, operates at moderate temperatures, and relies on widely available chemical precursors. From a technological perspective, the vapor-phase nature of the process is potentially compatible with larger-scale production, as analogous gas-phase carbon formation and deposition approaches are already implemented at large scale, and the two-zone configuration used here can, in principle, be translated to (semi)continuous reactor systems such as rotary kilns, fluidized-bed reactors, and roll-to-roll systems.

## Experimental section

### Material synthesis

Carbon films were deposited onto various substrates, including SiO_2_ wafers (MicroChemicals), activated carbon fiber (ACF, Kynol-ACC-5092-20), activated carbon powder (ACP, DARCO), carbide-derived carbon (CDC), KetjenBlack (KB, EC-600JD), activated microwave-expanded graphite oxide (aMEGO), coconut-shell-based porous carbon (CSBC), wood-based porous carbon (WBC), CMK-3, and hard carbon (Kuraray). The depositions were performed using a two-zone CVD system (GROW-2S-OS, planarTECH) equipped with a 2-inch quartz tube. The substrates (*ca.* 300 mg) were placed in Zone 2 inside a quartz boat, while the precursors (*ca.* 4 g) for carbon films, thiophene-2-carbaldehyde, 1*H*-pyrrole-2-carbaldehyde, and 5-(hydroxymethyl)furan-2-carbaldehyde (Sigma-Aldrich), were loaded in the center of Zone 1. Prior to heating, the reactor chamber was evacuated to a base pressure of approximately 10^−4^ Torr and subsequently purged with nitrogen to remove residual oxygen. The system was then backfilled with nitrogen to atmospheric pressure (760 Torr) before initiating the temperature ramp. Zone 2 was then heated to 800 °C at a rate of 10 °C min^−1^, followed by heating Zone 1 to 300 °C to initiate precursor vaporization. A steady nitrogen flow (20 sccm) transported the precursor vapor into Zone 2, where it thermally condensed on the substrate surfaces. The deposition was maintained for 240 min, followed by natural cooling to room temperature. The resulting samples were designated as Cfilm_HMF_/X, Cfilm_THP_/X, and Cfilm_PYR_/X (where X is the substrate).

### General electrochemical measurements

Electrochemical measurements were performed using three-electrode Swagelok-type cells connected to a Biologic MPG-2 workstation (France). Cell assembly was conducted in an argon-filled glovebox (MBRAUN, Germany) with O_2_ and H_2_O levels maintained below 0.1 ppm. Sodium metal (99.5%, Sigma-Aldrich) served as both counter and reference electrode, while a glass fiber membrane (Whatman GF/C) was employed as the separator. The electrolyte was 1M NaPF_6_ in EC/EMC (3 : 7 v, 200 µl), supplied by E-Lyte GmbH (Germany). The active material loading was approximately 1.5 mg cm^-2^ for all electrodes to ensure consistent comparison. GCD measurements were carried out at room temperature in the potential window of 0-3.0 V (*vs.* Na^+^/Na) after a three-hour equilibration period. CV scans were performed at scan rates of 0.05–2.0 mV s^−1^ between 0–2.5/3.0 V (*vs.* Na^+^/Na). EIS measurements were conducted using a 10 mV AC perturbation over a frequency range of 0.1 Hz to 20 kHz. The DRT spectra were extracted and analyzed using a custom-built MATLAB script. Sodium-ion diffusion coefficients were determined *via* the GITT, employing current pulses of 30 mA g^−1^ for 600 s followed by relaxation steps of 3600 s. Diffusion coefficients were obtained through data fitting using a custom script developed in RStudio. The sheet resistance of the carbon films was measured using a four-point probe setup (Ossila, UK).

### Physicochemical characterizations

The crystallinity of the samples was analyzed by GIXRD using a Rigaku SmartLab diffractometer (Japan). SAXS/WAXS measurements were performed at the µSpot beamline of BESSY-II (Helmholtz-Zentrum Berlin, Germany) using a monochromatic X-ray beam (18.0 keV, 30 µm) defined by pinhole collimation. The sample transmission was measured *via* the X-ray fluorescence signal collected by a RAYSPEC Sirius SD-E65133-BE-INC detector with an 8 µm beryllium window placed in front of a lead beam stop, while scattered intensities were recorded using a Dectris Eiger 9M detector. The incident beam intensity was monitored using an ionization chamber, and the recorded values were used to normalize the scattering signal. The scattering *q*-range was calibrated using silver behenate, and the intensities were normalized against glassy carbon (NIST SRM3600). Data reduction and processing were performed using in-house Python software built on the pyFAI library. X-ray total scattering experiments were conducted at the Powder Diffraction and Total Scattering Beamline P02.1 of PETRA III (Hamburg, Germany).^55^ The experiment was conducted at an X-ray wavelength of *λ* = 0.207351 Å using a Varex XRD 4343CT area detector (pixel size 150 × 150 µm^2^; pixel area 2880 × 2880 pixels). Data were collected with the direct beam centered in the detector, acquiring full Debye–Scherrer rings. Each measurement was obtained with a total exposure time of 900 s by merging 60 s scans to ensure valid dark measurements and the highest possible data quality. The acquired 2D patterns were azimuthally integrated using the PyFAI software package.^56^ Azimuthal integration was calibrated using a Si standard (99.9%, APS 1–5 micron, Alfa Aesar) mounted between sheets of Kapton, resulting in a refined sample-to-detector distance of 228.77 mm. When integrating the 2D patterns, a mask covering the 10 outermost pixels, beamstop, beamstop arm, and dead pixels was applied. The resulting 1D powder patterns underwent background subtraction and normalization, then were Fourier-transformed to obtain the pair distribution function with the PDFgetX3 algorithm in xPDFsuite. The PDFs were obtained using *q*_min_ = 0.5 Å^−1^, *q*_max_ = 22.0 Å^−1^, *q*_max-inst_ = 22.0 Å^−1^, and *r*_poly_ of 0.88 Å. XPS measurements were carried out with an Axis Supra+ (Kratos Analytical, UK) with monochromatized Al K_α_ radiation used for excitation (15 kV, typical 20 mA). Work function measurements were conducted using PESA with a Riken AC-2 spectrometer (Japan). Raman spectra were recorded on a WITec Alpha 300R confocal Raman microscope (Germany) using a 532 nm laser at 10 mW with a 20 s integration time. Data analysis was performed in WITec Project FIVE 5.2, and the D and G bands were fitted using a PseudoVoigt function. TGA-MS was conducted on a NETZSCH TG-209 Libra (Germany) under a helium atmosphere at a heating rate of 2.5 K min^−1^. Gas physisorption measurements were performed on a Quantachrome Quadrasorb SI analyzer (Austria) at 273 K for CO_2_ and 77 K for N_2_ and Ar after overnight degassing. SEM images were obtained on a Zeiss LEO 1550-Gemini microscope (Germany) at acceleration voltages of 3–10 kV, with EDX spectra collected using an Oxford Instruments X-MAX 80 mm^2^ detector (UK). HRTEM imaging was performed on a double aberration-corrected JEOL JEM ARM200F (Japan) equipped with a cold field emission gun operating at 80 kV with an extraction voltage of 10 µA. Images were acquired with a Oneview (4k × 4k) camera. Data processing was conducted using Gatan Microscopy Suite version 3.4. Ultramicrotome sectioning was conducted using a Leica ultramicrotome (Germany) equipped with a Diatome Ultra 35° diamond knife.

## Author contributions

E.O. Eren conceived and designed the work, performed the experiments, and wrote the original manuscript. E. Senokos and T. Horner contributed to materials characterization and data discussion. L. Cancellara conducted TEM measurements. E. Scoppola conducted SAXS/WAXS measurements. J. Kim conducted XPS/PESA measurements. M. Johansen conducted PDF analysis. K. Ge, B. Daffos, P.-L. Taberna, P. Simon contributed to electrochemical characterization and data discussion. P. Giusto contributed to the design and supervision of the study. All authors discussed the results and agreed on the final version of the manuscript.

## Conflicts of interest

The authors disclose that a patent application covering aspects of the vapor-phase carbon film deposition method reported in this study has been filed. No other competing interests are declared.

## Supplementary Material

EE-019-D6EE01852A-s001

## Data Availability

The data supporting this article have been included as part of the Supplementary information (SI). Supplementary information is available. See DOI: https://doi.org/10.1039/d6ee01852a.
