# Supplementary material for: CVD-grown tunable carbon films for high-performance sodium storage
Source: Energy Environ Sci. 2026 Jun 12;19(14):4603–15. doi: 10.1039/d6ee01852a (PMC13280718; doi:10.1039/d6ee01852a)
Supplement: EE-019-D6EE01852A-s001 [file EE-019-D6EE01852A-s001.pdf]

Supplementary Information (SI) for Energy & Environmental Science.
This journal is © The Royal Society of Chemistry 2026

Supplementary Information

CVD-grown tunable carbon films for high-performance sodium storage

Enis Oğuzhan Eren,[a] Evgeny Senokos,[a] Tim Horner,[a] Leonardo Cancellara,[a] Ernesto Scoppola,[b] Jiyong Kim,[c] Morten Johansen,[d] Kangkang Ge,[e,f] Barbara Daffos,[e,f] Pierre-Louis Taberna,[e,f] Patrice Simon,[e,f] and Paolo Giusto,[a*]*

[a]*Department of Colloid Chemistry, Max Planck Institute of Colloids and Interfaces, 14476 Potsdam, Germany*

[b]*Department of Biomaterials, Max Planck Institute of Colloids and Interfaces, 14476 Potsdam, Germany*

[c]*Functional Materials and Devices, Fraunhofer Institute for Applied Polymer Research IAP, 14476 Potsdam, Germany*

[d]*Deutsches Elektronen-Synchrotron DESY, Notkestr. 85, 22607 Hamburg, Germany*

[e]*Université de Toulouse, CIRIMAT UMR CNRS 5085, 118 Route de Narbonne, 31062 Toulouse, France*

[f]*Réseau sur le Stockage Electrochimique de l'Energie (RS2E), Rue Beaudelocque, 80000 Amiens, France*

[*]*e-mail: enis.eren@mpikg.mpg.de; paolo.giusto@mpikg.mpg.de*

Contents

Supplementary Note 1. Volatilization and decomposition pathways of heterocyclic aldehyde precursors 3
Fig. S1....4
Supplementary Note 2. Proof-of-concept: Versatile deposition of carbon film on Cu foil....5
Fig. S2....6
Supplementary Note 3. Morphological characterization of carbon films on SiO_2 substrates....7
Fig. S3....8
Supplementary Note 4. Structural ordering and electronic properties of carbon films 9
Fig. S4....10
Supplementary Note 5. Structural integration of carbon films with activated carbon fibers 11
Fig. S5....12
Fig. S6....13
Fig. S7....14
Supplementary Note 6. Micropore confinement in Cfilm/ACF revealed by CO_2 and N_2 sorption....15
Fig. S8....16
Supplementary Note 7. Precursor and thickness effects on Cfilm/ACF structure probed by SAXS/WAXS 17
Fig. S9....20
Fig. S10....21
Supplementary Note 8. Surface chemistry of Cfilms revealed by XPS 22
Fig. S11....23
Supplementary Note 9. Sheet resistance and electrical conductivity of Cfilm/SiO_2 and Cfilm/ACF samples 24
Table S1....24
Supplementary Note 10. Electrochemical kinetics from CV and *b*-value analysis....25
Fig. S12....26
Supplementary Note 11. Morphological and chemical analysis of the SEI via SEM and XPS 27
Fig. S13....30
Fig. S14....31
Fig. S15....32
Fig. S16....33
Supplementary Note 12. *In-situ* EIS of interfacial processes in pristine and Cfilm/ACF electrodes....34
Table S2....36
Fig. S17....37
Supplementary Note 13. DRT analysis of charge-transfer and diffusion processes during sodiation 38
Fig. S18....40
Supplementary Note 14. GITT Analysis and Mechanistic Insights 41
Fig. S19....44
Fig. S20....45
Ex-situ PDF analysis....45
Fig. S21....47
Supplementary Note 15. Effect of deposition temperature on structure and electrochemical performance 48
Fig. S22....50
Supplementary Note 16. Extension of the $Cfilm_{PYR}$ to different porous carbons....51
Fig. S23....53
Fig. S24....54
Fig. S25....55
Fig. S26....56
Supplementary Note 17. Rate capability and cycling stability assessment of Cfilm-coated electrodes....57
Fig. S27....60
Fig. S28....61
Fig. S29....62
Supplementary Note 18. Benchmarking plateau capacities of $Cfilm_{PYR}$-coated carbons....63
Table S3....64
Fig. S30....65
Supplementary Note 19. Full-cell evaluation of $Cfilm_{PYR}$/ACF....66
Fig. S31....67
References....68

Supplementary Note 1. Volatilization and decomposition pathways of heterocyclic aldehyde precursors

To gain insight into the volatilization and thermal decomposition behavior of the heterocyclic aldehyde precursors, thermogravimetric analysis coupled with mass spectrometry (TGA-MS) was conducted (**Fig. S1**). Thiophene-2-carbaldehyde (**Fig. S1a**) and 1*H*-pyrrole-2-carbaldehyde (**Fig. S1b**) both exhibit sharp mass losses below 100 °C.

Thiophene-2-carbaldehyde shows distinct fragment peaks during volatilization (**Fig. S1d**), including a strong m/z 111 signal corresponding to $C_5H_3OS^+$, which represents a stabilized fragment closely related to the parent molecule. Additional fragments include CO_2^+ (m/z 44), CO^+ (m/z 28), and light hydrocarbon fragments (m/z 26-39), reflecting aldehyde decomposition and thiophene ring cleavage. These findings confirm that the majority of thiophene aldehyde is transported in the vapor phase, with sulfur largely preserved in higher-mass fragments rather than SO_x.

1*H*-pyrrole-2-carbaldehyde follows a similar pathway (**Fig. S1e**), with clear CO_2^+ (m/z 44) and CO^+ (m/z 28) signals alongside nitrogen-containing species such as NO^+ (m/z 30) and NH_3^+ (m/z 17). These fragments indicate that the pyrrole ring decomposes while still retaining nitrogen functionalities, thereby facilitating their incorporation into the resulting carbon film. Together, the TGA-MS analysis confirms that both thiophene and pyrrole aldehydes volatilize cleanly, providing a continuous supply of vapor-phase precursor molecules. Their decomposition produces well-defined heteroatom-containing fragments that directly rationalize the sulfur and nitrogen signatures in the corresponding carbon films.

In contrast, 5-(hydroxymethyl)furan-2-carbaldehyde (HMF) reveals a different behavior (**Fig. S1c, f**). The TGA profile shows a broad decomposition window extending up to 250 °C, reflecting reduced volatility and a tendency toward thermal polymerization.[1] Furthermore, no distinct low- and high-mass fragments are detected in the MS spectra under identical conditions. We attribute this absence of volatile fragment signals to the multifunctional nature of HMF, which promotes condensation and cross-linking reactions upon heating, as observed previously.[1] As a result, the decomposition pathway likely produces oligomeric or polymeric intermediates that either condense before reaching the MS detector or fail to volatilize at measurement conditions. Still, under CVD conditions, film formation from HMF was observed to be highly efficient and comparable to that of the other precursors, indicating that despite its reduced volatility, HMF can effectively contribute to uniform carbon growth.

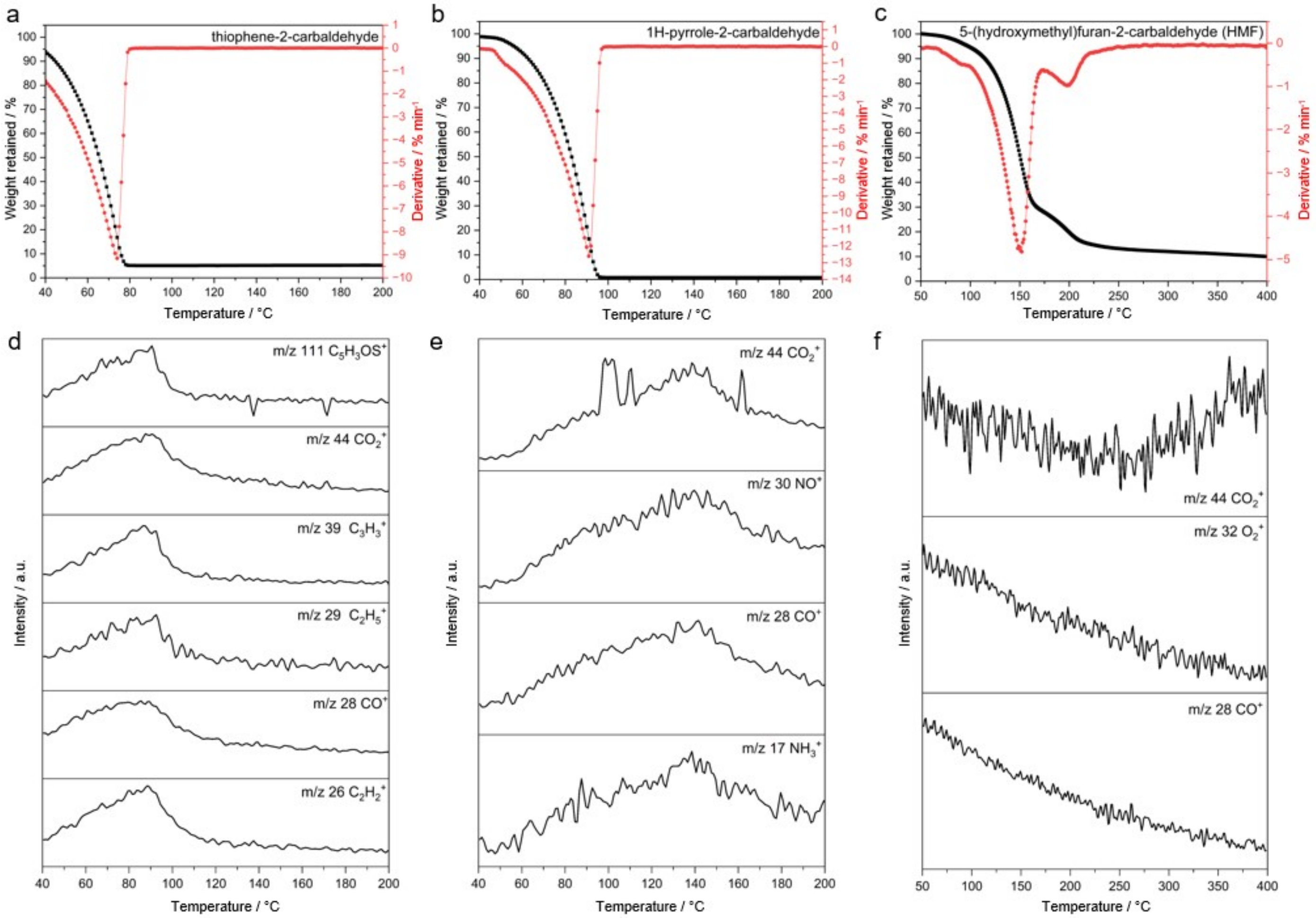

Fig. S1. TGA-MS analysis of heterocyclic precursors used for carbon film deposition. TGA (black, left axis) and DTG (red, right axis) profiles of **(a)** thiophene-2-carbaldehyde, **(b)** 1*H*-pyrrole-2-carbaldehyde, and **(c)** 5-(hydroxymethyl) furan-2-carbaldehyde (HMF), showing distinct volatilization behaviors. The sharp mass losses of thiophene and pyrrole aldehydes occur at lower temperatures (50-100 °C), while HMF exhibits a broader and higher-temperature decomposition range (100-250 °C). **(d, e, f)** Corresponding MS showing the evolution of major decomposition products as a function of temperature for each precursor. **(d)** thiophene-2-carbaldehyde, **(e)** 1*H*-pyrrole-2-carbaldehyde, and **(f)** 5-(hydroxymethyl)furan-2-carbaldehyde.

Supplementary Note 2. Proof-of-concept: Versatile deposition of carbon film on Cu foil

To evaluate the versatility of the CVD approach, we extended carbon film deposition to a metallic substrate. Using HMF as the precursor and copper foils as substrates, conformal carbon films were successfully obtained (**Fig. S2a**). The SEM image demonstrates complete coverage of the Cu surface by a continuous film, while the inset optical image shows that the resulting Cfilm/Cu remains mechanically flexible.

Elemental mapping (**Fig. S2b**) confirms the uniform distribution of carbon on top of the copper substrate, with oxygen signals. The smooth and continuous coverage is indicative of efficient precursor utilization and stable film growth on metallic surfaces. Optical microscopy of the Cfilm/Cu interface (**Fig. S2c**) reveals a wrinkled morphology, which is attributed to thermal expansion mismatch between the carbon film and copper during the thermal treatment.

The Raman spectrum (**Fig. S2d**) displays the characteristic *D*, *G*, and 2*D* bands, with an I_{D1}/I_G ratio of 0.81, consistent with the $Cfilm_{HMF}$ reported on a SiO_2 substrate in the main manuscript. Raman mapping further confirms lateral homogeneity across the Cu surface.

This proof-of-concept deposition on copper illustrates the adaptability of the CVD approach to a variety of substrates beyond inert oxides. By demonstrating compatibility with a metal foil, the platform paves the way for applications in electrochemical devices, such as flexible electrodes or catalyst supports. The wrinkled yet continuous film morphology could also be advantageous in applications requiring flexibility.

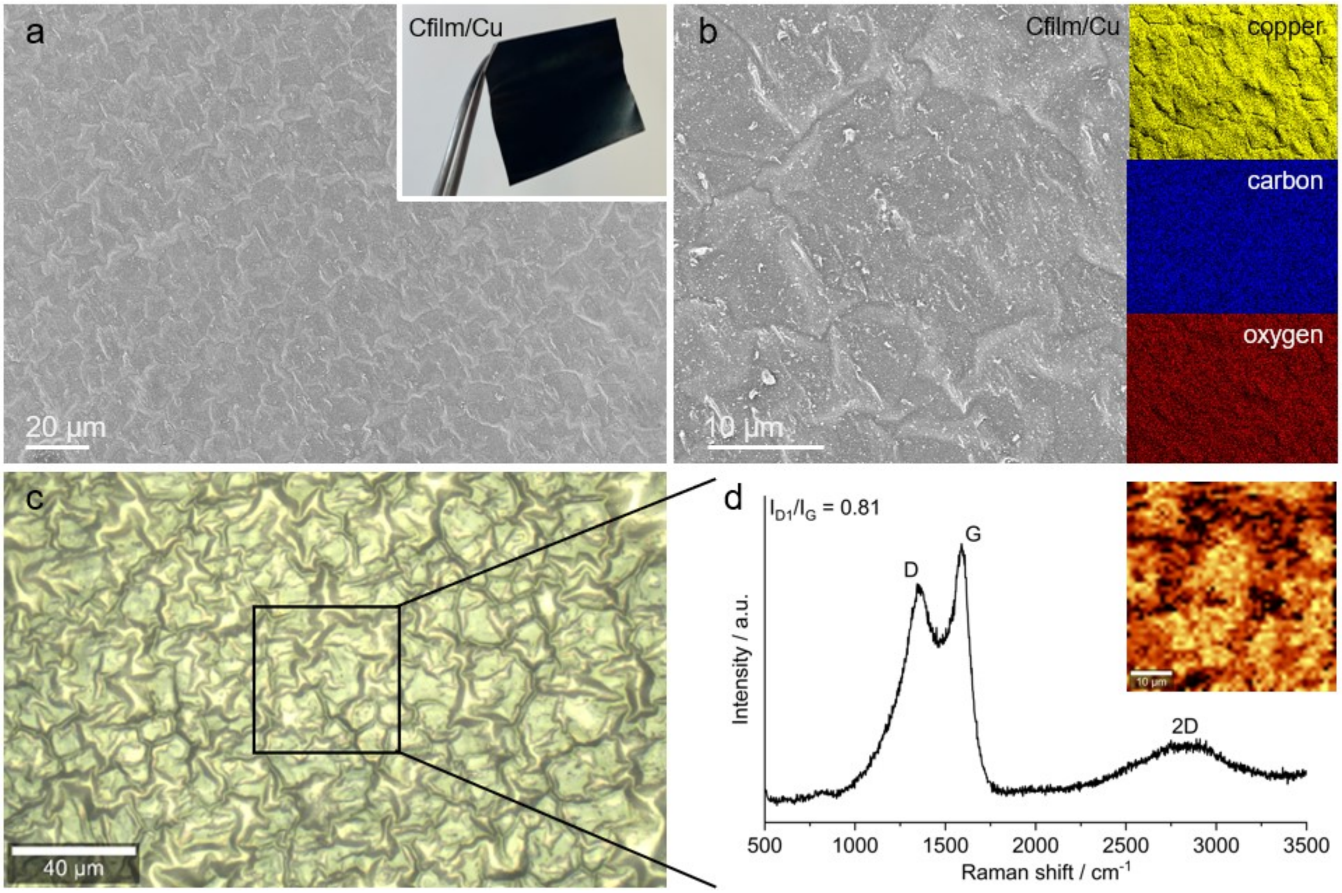

Fig. S2. Morphological and structural characterization of carbon film from 5-(hydroxymethyl)furan-2-carbaldehyde (HMF) deposited on Cu substrates via the CVD method. **(a)** SEM image of a Cfilm/Cu sample showing a continuous and uniform carbon film; inset: optical image of a flexible Cfilm/Cu sheet. **(b)** SEM image of the Cfilm/Cu surface with corresponding EDS elemental maps confirming the uniform distribution of copper (from substrate), carbon, and oxygen. **(c)** Optical microscopy image of the Cfilm/Cu surface, showing characteristic wrinkled morphology arising from thermal expansion mismatch between the film and copper substrate. **(d)** Raman spectrum of the Cfilm/Cu film shows prominent *D*, *G*, and 2*D* bands, with an I_{D1}/I_G ratio of 0.81.

Supplementary Note 3. Morphological characterization of carbon films on SiO_2 substrate

The carbon films were deposited on SiO_2 wafers, selected here as an inert and thermally stable substrate for surface inspection (**Fig. S3a**).

The scanning electron microscopy (SEM) analysis (**Fig. S3b**) reveals smooth, conformal coatings with a high degree of homogeneity, regardless of the precursor. The energy-dispersive X-ray spectroscopy (EDS) elemental mapping confirms the incorporation of heteroatoms inherited from the precursor chemistry. Specifically, $Cfilm_{THP}$ shows sulfur and oxygen retention, $Cfilm_{PYR}$ contains nitrogen and oxygen, and $Cfilm_{HMF}$ retains only oxygen. These signals are uniformly distributed across the mapped area, ruling out phase separation or localized inhomogeneities of specific elements.

The cross-sectional SEM images (**Fig. S3c**) further highlight the conformality of the Cfilms, with thicknesses ranging between a few hundred nanometers and a micron scale, mainly depending on the precursor amount provided. Importantly, the interface between the carbon film and the underlying SiO_2 appears well integrated, without visible delamination or interfacial gaps. In the right panel, a physically detached film is shown. This detached segment retains its flat morphology and internal cohesion, suggesting that the film is not only continuous but also sufficiently robust to exist as a freestanding material. This observation is essential, as it points toward the potential applicability of these carbon films in layered assemblies, as semi-flexible conductive films in many systems.

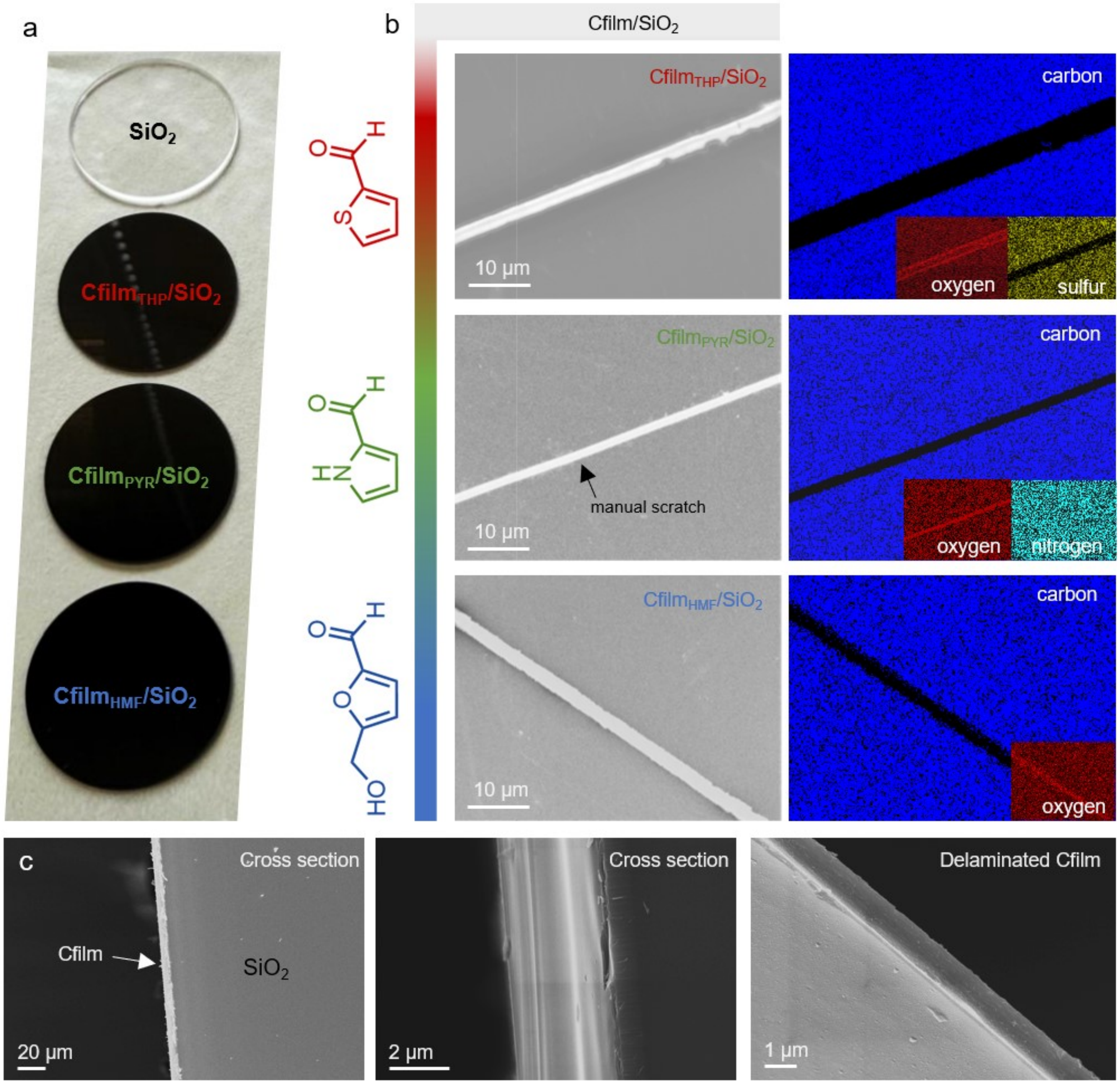

Fig. S3. Synthesis and morphological characterization of Cfilms derived from heterocyclic aldehyde precursors on SiO_2 substrates. **(a)** Optical images of bare SiO_2 and carbon-coated SiO_2 substrates prepared using three different precursors: thiophene-2-carbaldehyde ($Cfilm_{THP}$), 1*H*-pyrrole-2-carbaldehyde ($Cfilm_{PYR}$), and 5-(hydroxymethyl)furan-2-carbaldehyde ($Cfilm_{HMF}$). **(b)** SEM images and corresponding EDS elemental maps of Cfilm/SiO_2 films, showing uniform and conformal film coverage. **(c)** Cross-sectional SEM images of Cfilm/SiO_2 structures prepared, revealing uniform carbon film thickness and intimate substrate-film interfaces. The rightmost image shows a delaminated carbon film.

Supplementary Note 4. Structural ordering and electronic properties of carbon films

The structural order of the carbon films was first assessed by Raman spectroscopy (**Fig. S4a-c**). All samples exhibit the disorder-induced *D* band (~1347 cm^{-1}), the in-plane graphitic *G* band (~1575 cm^{-1}), and a broader 2*D* feature, consistent with turbostratic carbons.[2, 3, 4] The I_{D1}/I_G ratios reported in the main manuscript (**Fig. 1e**) are 0.87 for $Cfilm_{THP}$, 0.77 for $Cfilm_{PYR}$, and 0.81 for $Cfilm_{HMF}$, confirming precursor-dependent differences in defect density and ordering. Grazing incidence X-ray diffraction (GIXRD) patterns (**Fig. S4d-g**) complement this analysis. The bare SiO_2 substrate displays only a broad amorphous peak at 21.5°, while carbon-coated samples reveal (002) reflections at 25.7 and 26.0°. Bragg's law (λ = 1.5418 Å) yields interlayer distances of 3.47 Å ($Cfilm_{THP}$), 3.47 Å ($Cfilm_{HMF}$), and 3.43 Å ($Cfilm_{PYR}$). These *d*-spacings, compared to crystalline graphite (3.35 Å), reflect the turbostratic stacking and slight structural disorder. Full-width at half-maximum values (4.4-4.7°) indicate small coherent stacking domains, further supporting the presence of pseudo-graphitic nanoregions. Still, when compared to non-graphitizing (hard) carbons, these findings suggest that the films possess a greater tendency toward structural ordering.[5] The relatively sharp (002) reflections, moderate interlayer spacings (3.43-3.47 Å), and balanced I_{D1}/I_G ratios point to a partial evolution toward graphitic domains, in contrast to the highly disordered, micropore-rich frameworks of hard carbons. Such features are more consistent with soft carbons, which are known to undergo graphitization under high temperatures.[5] Accordingly, the heterocyclic precursor-derived Cfilms investigated here may be regarded as soft-carbon-type materials.

Electronic properties were investigated by photoelectron spectroscopy in air (PESA-AC2), a contactless technique particularly suitable for films on insulating substrates (**Fig. S4h-j**).[6] The work functions (W_f) obtained are 4.84 eV for $Cfilm_{HMF}$, 4.97 eV for $Cfilm_{PYR}$, and 4.88 eV for $Cfilm_{THP}$. These values are consistent with moderately doped carbons, where heteroatom incorporation shifts the Fermi level position.[7] Nitrogen incorporation in $Cfilm_{PYR}$ increases the W_f, reflecting deeper Fermi levels.

Importantly, these work function variations have direct electrochemical relevance. A higher W_f corresponds to a lower Fermi level, which influences its alignment with the electrolyte's lowest unoccupied molecular orbital (LUMO). This shift endows the carbon surface with a more noble electronic character, meaning it is energetically less prone to donate electrons, thereby potentially suppressing the onset of electrolyte reduction. Thus, precursor-derived differences in electronic structure, as revealed by PESA, provide a rational link to the later electrochemical behavior, including variations in SEI formation and sodium storage kinetics.

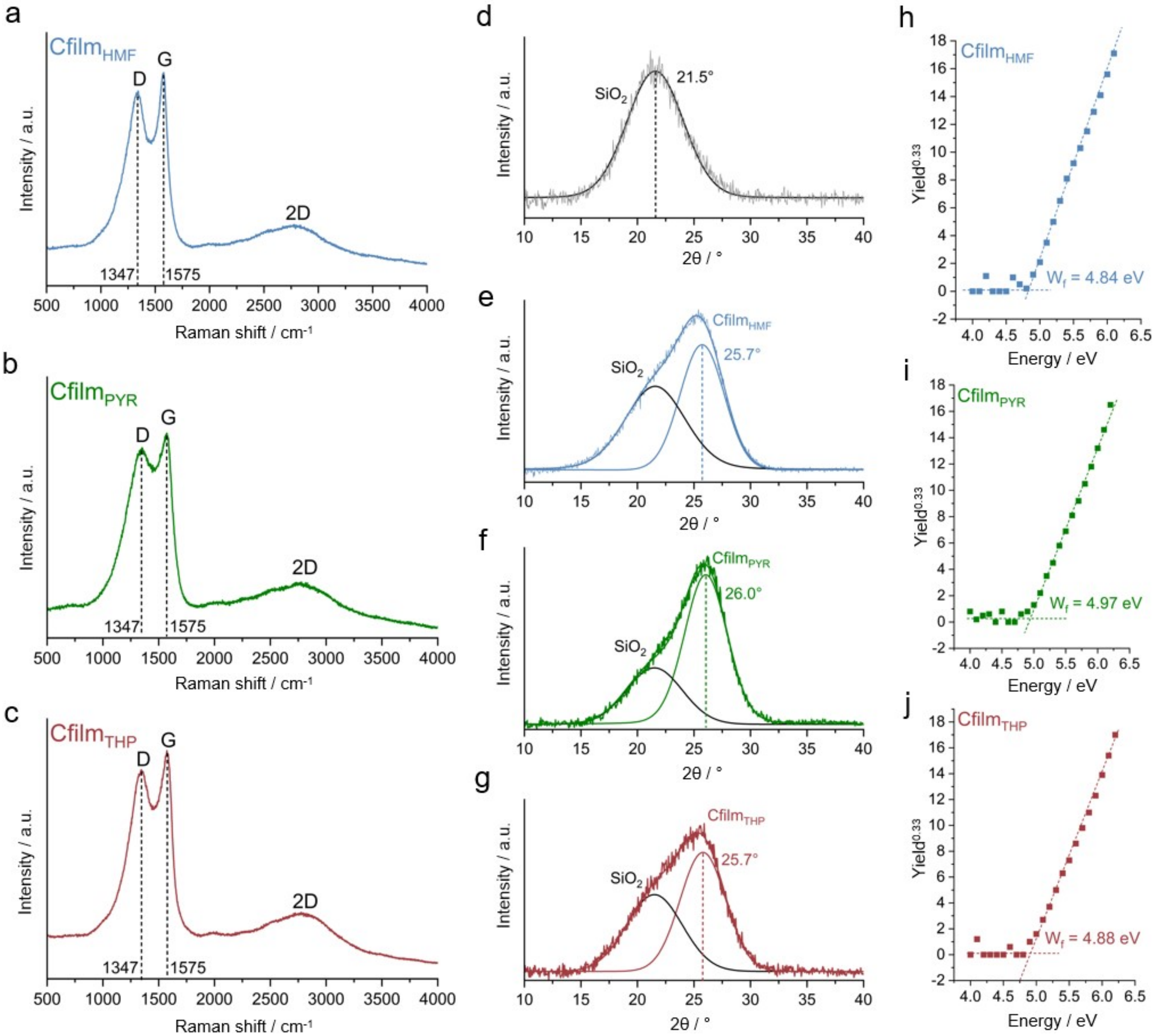

Fig. S4. Spectroscopic and structural analysis of carbon films derived from different heterocyclic precursors. **(a-c)** Raman spectra of $Cfilm_{HMF}$ (blue), $Cfilm_{PYR}$ (green), and $Cfilm_{THP}$ (red), showing characteristic *D*, *G*, and 2*D* bands. The *D* and *G* band positions (1347 cm^{-1} and 1575 cm^{-1}, respectively) are consistent across samples, with varying intensity ratios indicating differences in structural order. **(d-g)** GIXRD patterns of bare SiO_2 and carbon-coated SiO_2 substrates. Bare SiO_2 shows a broad amorphous hump centered at 21.5°, while carbon-coated samples show sharper (002) peaks at higher angles ($Cfilm_{HMF}$: 25.7°, $Cfilm_{PYR}$: 26.0°, $Cfilm_{THP}$: 25.7°). **(h-j)** Photoelectron yield spectra obtained by PESA measurements, used to determine the work functions (W_f) of each film. The extracted work function values are 4.84 eV ($Cfilm_{HMF}$), 4.97 eV ($Cfilm_{PYR}$), and 4.88 eV ($Cfilm_{THP}$), revealing precursor-dependent electronic properties.

Supplementary Note 5. Structural integration of carbon films with activated carbon fibers

SEM imaging at multiple magnifications (**Fig. S5**) shows that the overall fibrous macro-architecture of the pristine ACF scaffold is fully retained after Cfilm deposition. The inset optical image highlights a clear color change from black (pristine ACF) to dark gray (Cfilm/ACF), indicative of surface modification. At higher magnification, pristine ACF fibers exhibit a rough and irregular surface, characterized by inhomogeneities and processing artifacts rather than clearly resolved porosity. This irregular texture is consistent with the high density of oxygen-containing functional groups typically present in activated carbons, which contribute to the chemical heterogeneity.[8] In contrast, Cfilm/ACF fibers appear smoothened by the conformal carbon coating, producing a more uniform and continuous outer surface.

Cross-sectional SEM and TEM analyses (**Fig. S6**) provide direct evidence of the conformal Cfilm layer on ACF scaffolds. To obtain electron-transparent sections, the Cfilm/ACF samples were first coated with a thin Au protective layer by sputtering. This metallic overlayer was applied before ultramicrotomy to shield the outer Cfilm coating from mechanical damage during slicing. The samples were then embedded in epoxy resin and sectioned into electron-transparent thin slices (i.e., <100 nm thickness) using an ultramicrotome device, ensuring minimal deformation of both the delicate Cfilm coating and the porous ACF interior. The resulting cross sections clearly reveal a continuous Cfilm film tightly adhering to the fibrous ACF scaffold (**Fig. S6a-c**). The fracture and deformation lines due to sample preparation are sufficiently spread out to enable high-resolution TEM observations, in which the two domains can be clearly distinguished: the Cfilm layer shows a more ordered turbostratic arrangement (**Fig. S6d**), while the underlying ACF retains its highly disordered and microporous morphology (**Fig. S6e**).

In the Au-protected samples (**Fig. S6f-i**), the gold cap appears as the bright outermost layer in the TEM images and must not be mistaken for part of the Cfilm coating. Beneath this protective layer, both bright-field and dark-field TEM clearly identify the denser Cfilm shell surrounding the porous ACF core, with a sharp and intact interface between the two carbon phases. To further validate the thickness of the carbon films, annular dark-field scanning transmission electron microscopy (ADF-STEM) was employed (**Fig. S7**). Due to the higher density of the Cfilm layer relative to the ACF substrate, the carbon film appears with stronger contrast, enabling thickness estimation directly from the intensity gradient. The measured film thicknesses are approximately 11 ± 1 nm for $Cfilm_{THP}$, 36 ± 4 nm for $Cfilm_{HMF}$, and 71 ± 18 nm for $Cfilm_{PYR}$.

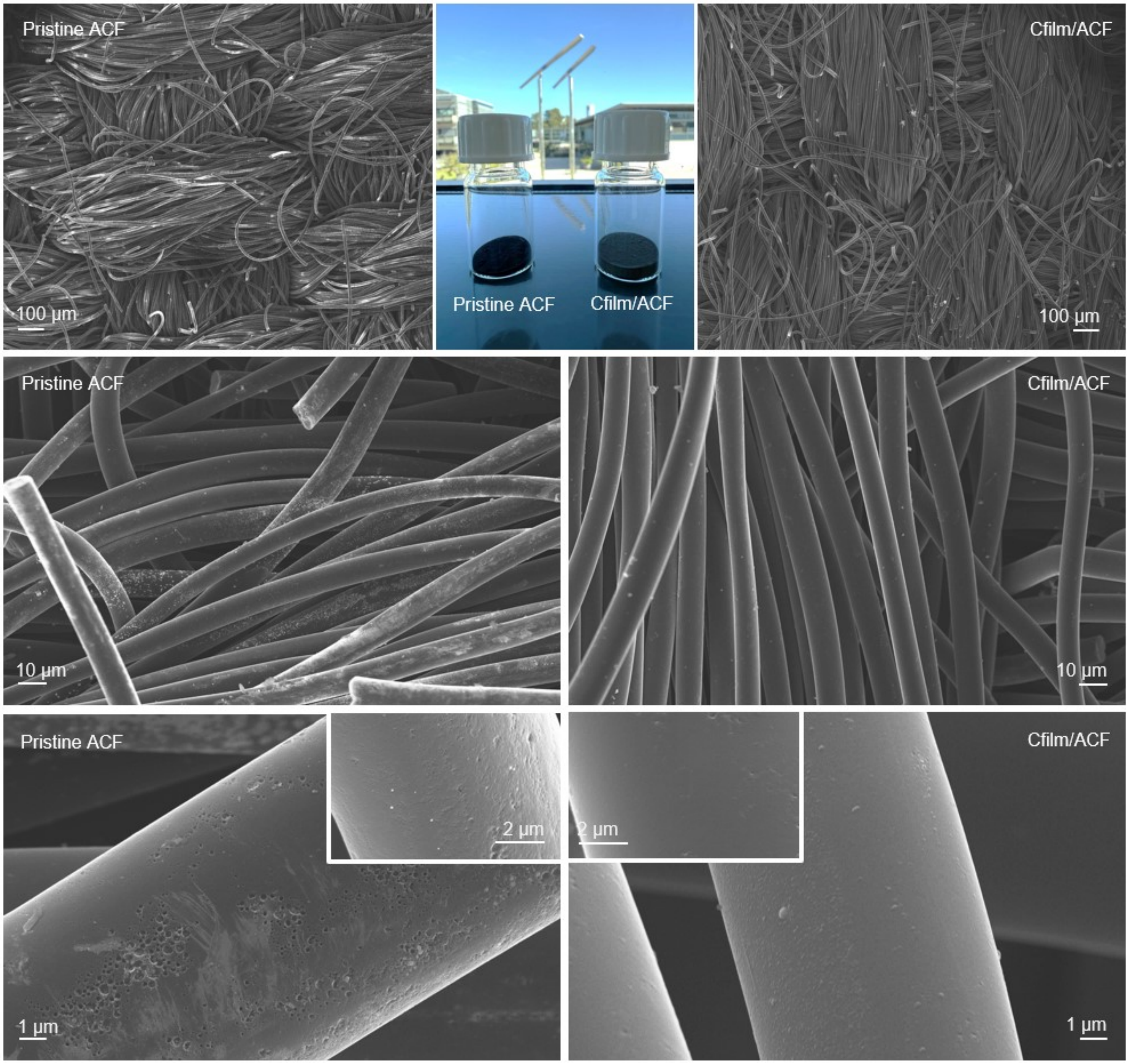

Fig. S5. Morphological comparison of pristine ACF and Cfilm/ACF. **Top row:** Low-magnification SEM images of pristine ACF and Cfilm/ACF reveal that the fibrous architecture is preserved after coating. Inset: Picture of pristine ACF and Cfilm/ACF powders, showing a distinct color change to dark gray for Cfilm/ACF due to the carbon film coating. **Middle row:** Higher-magnification SEM images show smoother fiber surfaces in Cfilm/ACF, in contrast to the rougher texture of pristine ACF. **Bottom row:** Close-up SEM images highlighting morphological differences between the pristine and coated fibers.

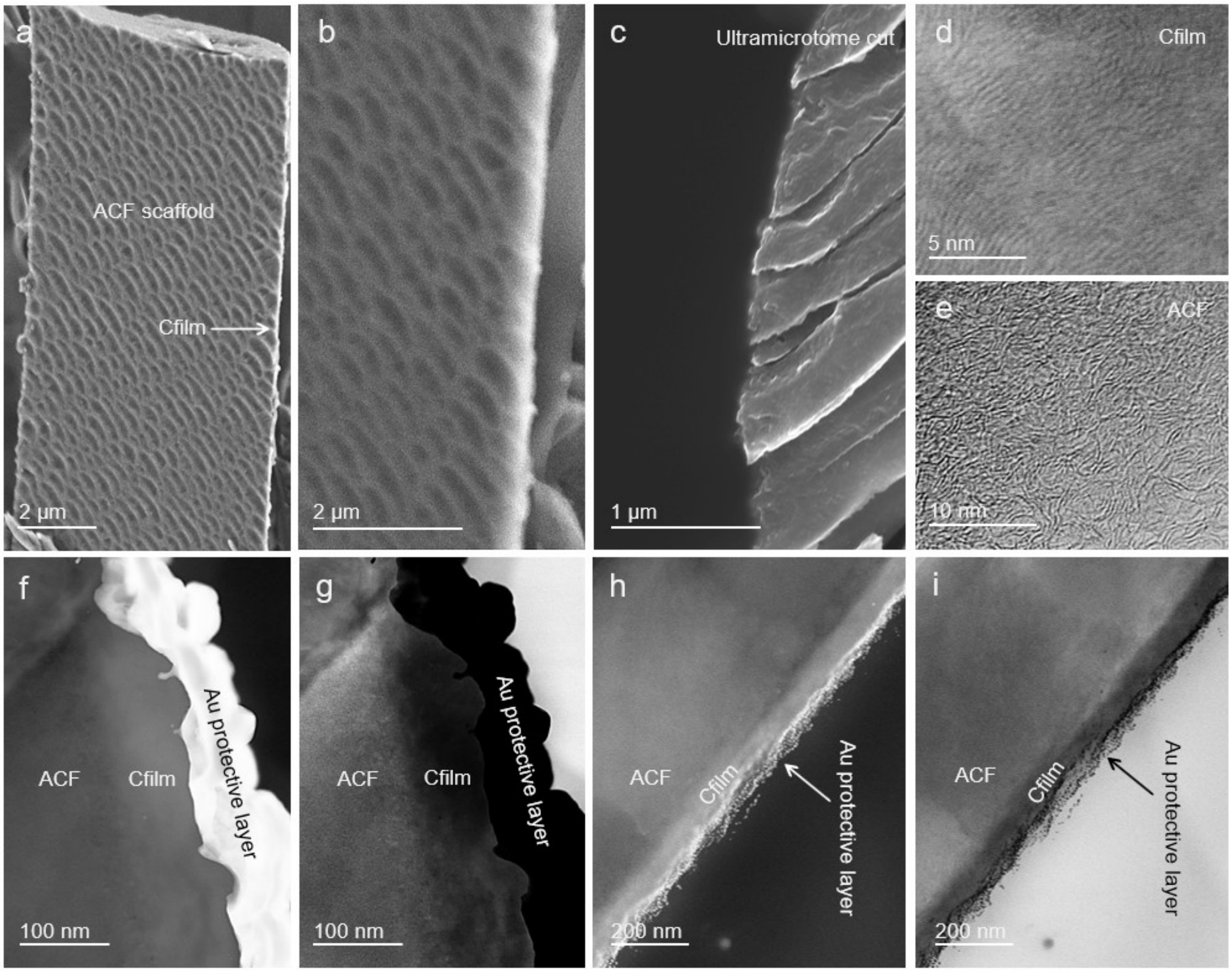

Fig. S6. Structural analysis of Cfilm/ACF materials using cross-sectional SEM and TEM. **(a-c)** SEM images of Cfilm/ACF prepared via ultramicrotomy, showing a conformal carbon film (Cfilm) coating over the porous ACF scaffold. HRTEM images of the **(d)** Cfilm and the underlying **(e)** ACF, respectively. The Cfilm displays a more ordered, layered structure, while the ACF retains a disordered, porous morphology. **(f, i)** Cross-sectional TEM images of Cfilm/ACF protected with a sputtered Au layer, which was deposited before ultramicrotomy to prevent surface damage during sectioning. Cross-sectional STEM images of Cfilm/ACF acquired in dark-field **(f, h)** and bright-field **(g, i)** modes. The difference in contrast between the two carbon phases is clearly visible, allowing a clear distinction between the denser Cfilm layer and the ACF substrate.

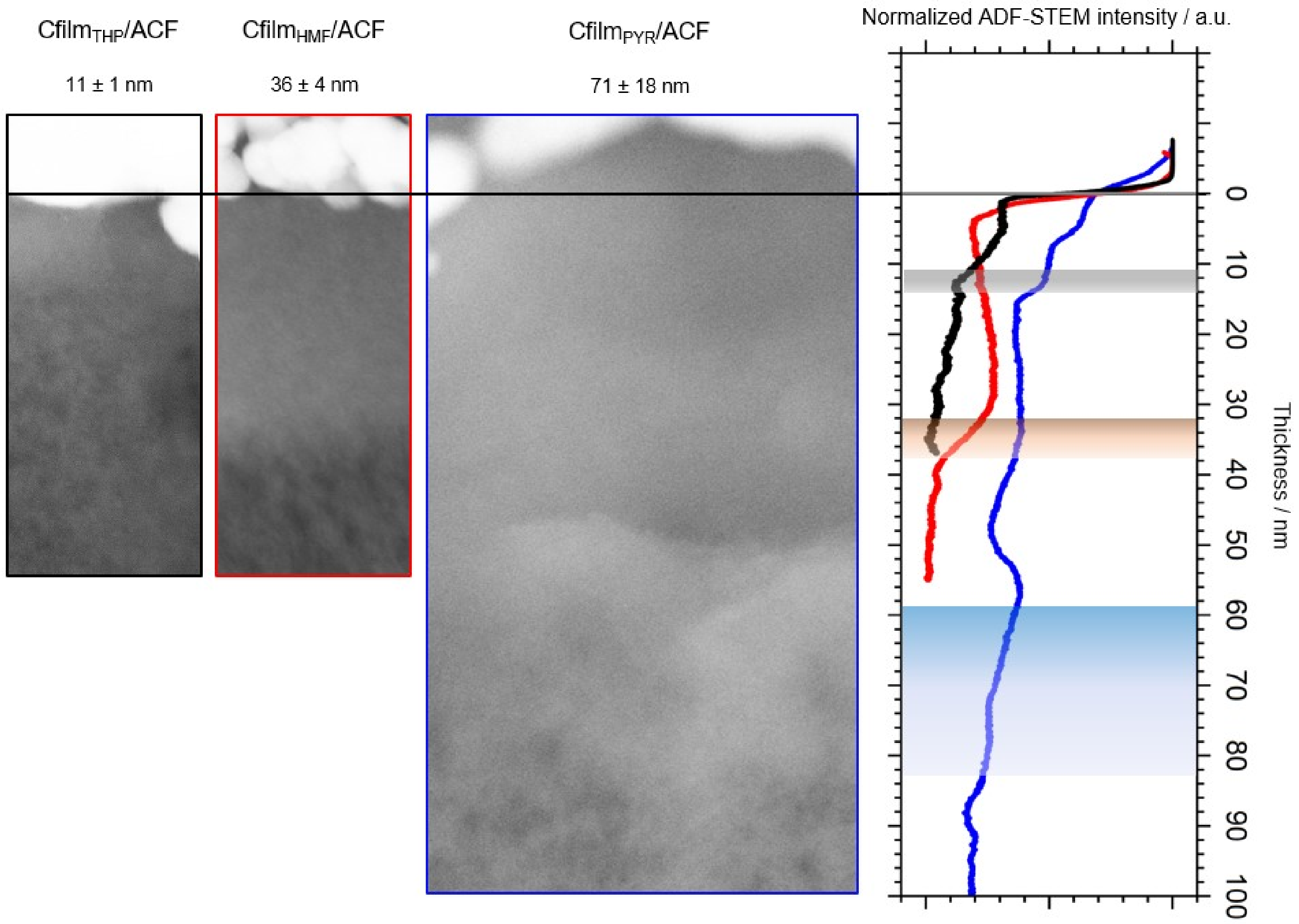

Fig. S7. ADF-STEM images of Cfilm$_{THP}$/ACF, Cfilm$_{HMF}$/ACF, and Cfilm$_{PYR}$/ACF, showing increasing carbon film thickness (11 ± 1 nm, 36 ± 4 nm, 71 ± 18 nm). Thickness was estimated from ADF contrast, as the denser carbon film appears brighter than the ACF. Right: Normalized Z-contrast intensity profiles, where shaded regions indicate the boundary between the carbon film and ACF substrate.

Supplementary Note 6. Micropore confinement in Cfilm/ACF revealed by CO_2 and N_2 sorption

To assess the effect of Cfilm coatings on the porosity of ACF substrates, we performed complementary CO_2 and N_2 physisorption measurements. CO_2 has a small kinetic diameter at the measurement conditions and higher diffusivity compared to N_2 at 77 K, allowing it to access ultramicropores (<1 nm) that N_2 often underestimates due to diffusion limitations.[9] Thus, CO_2 physisorption provides reliable information about micropores and pore size distributions in carbonaceous materials (**Fig. S8a**).

For pristine ACF, the CO_2 isotherm reveals a steep uptake at low relative pressures, indicative of a highly microporous structure, with a calculated SSA_{DFT} of 1361 $m^2 g^{-1}$. In contrast, Cfilm/ACF samples derived from different precursors exhibit reduced CO_2 uptake, with SSA_{DFT} values of 437, 403, and 83 $m^2 g^{-1}$ for $\mathrm{Cfilm_{HMF}}$/ACF, $\mathrm{Cfilm_{THP}}$/ACF, and $\mathrm{Cfilm_{PYR}}$/ACF, respectively. The decrease in CO_2-accessible surface area reflects effective micropore coverage and partial pore blocking by the conformal Cfilm layer. The corresponding pore size distributions (**Fig. S8b-e**) further illustrate this effect. While pristine ACF shows a broad distribution of micropores around 0.5-0.8 nm, Cfilm-coated samples exhibit suppressed or narrowed distributions, particularly below 0.7 nm, confirming that the Cfilm layer limits CO_2 penetration into the smallest micropores. The cumulative pore volume curves also demonstrate a significant reduction upon coating, consistent with surface confinement and micropore sealing.

To complement CO_2 sorption, N_2 adsorption-desorption isotherms were measured at 77 K (**Fig. S8f-g**). Pristine ACF exhibits substantial N_2 uptake at low relative pressures, yielding a high SSA_{BET} of 1630 $m^2 g^{-1}$. In contrast, all Cfilm/ACF samples show negligible N_2 uptake, indicating that the conformal Cfilm coating effectively blocks N_2 access to the porous network. This suppression is further reflected in the pore size distribution derived from N_2 sorption (**Fig. S8g**), which for pristine ACF shows a dominant micropore contribution below 1 nm. After the Cfilm deposition, however, N_2 uptake becomes negligible, indicating that these micropores are no longer accessible to N_2 at 77 K. The comparative analysis of specific surface areas (**Fig. S8h**) highlights this contrast. While pristine ACF shows very high surface areas from both CO_2 and N_2 physisorption, Cfilm/ACF samples exhibit values reduced by an order of magnitude, with near-zero SSA from N_2 sorption and only limited residual accessibility from CO_2. By restricting molecular access, the Cfilm coating regulates interfacial chemistry and prevents potential uncontrolled side reactions during electrochemical processes, such as extensive SEI formation.[10, 11] Among the samples, $\mathrm{Cfilm_{PYR}}$/ACF shows the thickest film in ADF-STEM (ca. 71 nm), which aligns with its lowest measured surface area in CO_2 sorption.

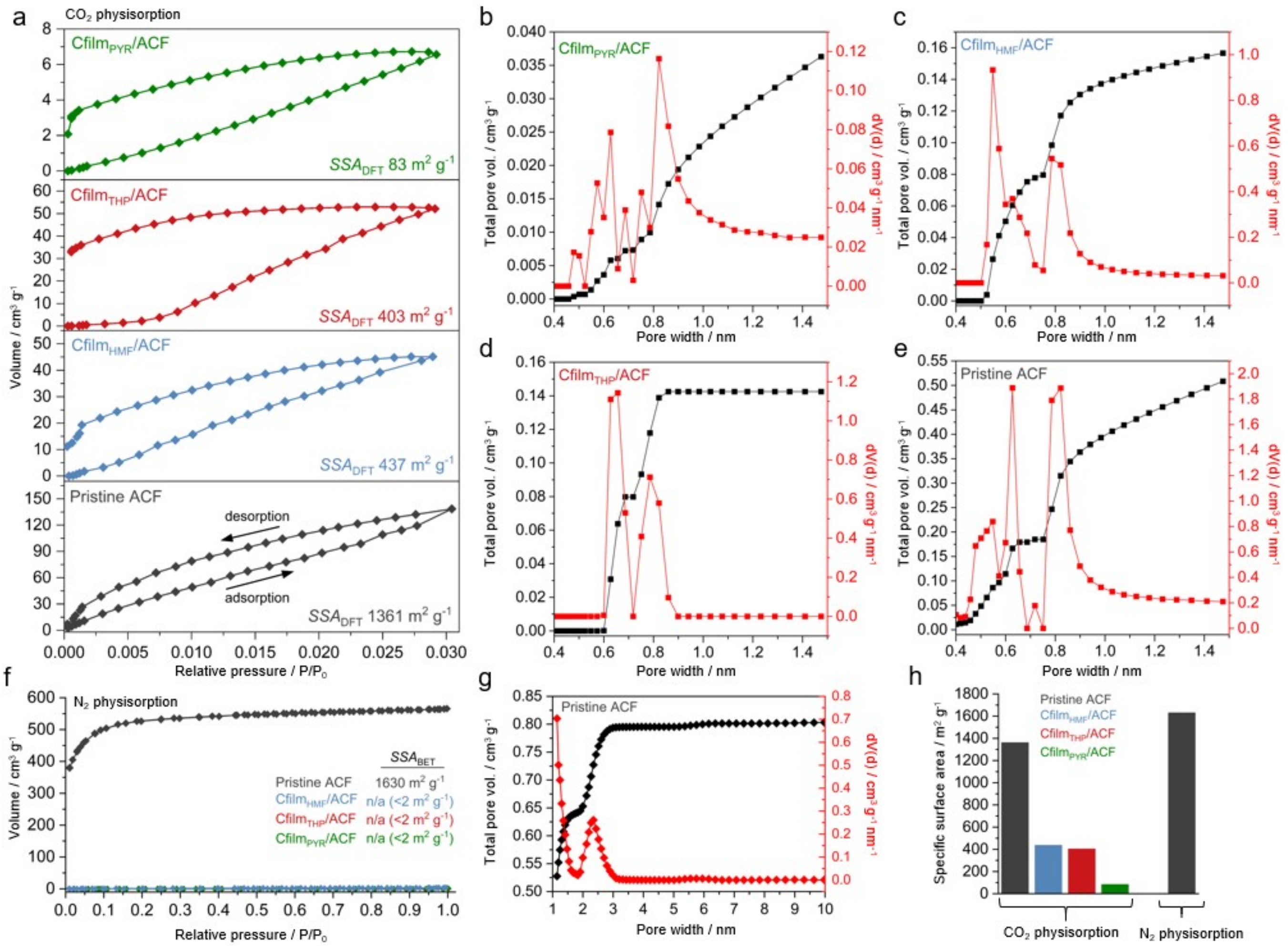

Fig. S8. Gas physisorption analyses of pristine ACF and Cfilm/ACF materials. **(a)** CO_2 physisorption isotherms at 273 K for pristine ACF and Cfilm/ACF samples derived from three different precursors, showing a progressive decrease in CO_2 uptake and SSA_{DFT} upon coating. **(b-e)** Pore size distributions and cumulative pore volumes derived from CO_2 physisorption data, showing suppression of micropores following Cfilm deposition for **(b)** $Cfilm_{PYR}$/ACF, **(c)** $Cfilm_{HMF}$/ACF, **(d)** $Cfilm_{THP}$/ACF, and **(e)** pristine ACF. **(f)** N_2 physisorption isotherms at 77 K, showing significant adsorption only for pristine ACF, while all Cfilm/ACF samples exhibit negligible uptake, indicating micropore confinement. **(g)** Cumulative pore volume and pore size distribution of pristine ACF from N_2 physisorption. **(h)** Bar graphs of specific surface areas calculated from both CO_2 and N_2 physisorption, confirming surface area suppression by the carbon film.

Supplementary Note 7. Precursor and thickness effects on Cfilm/ACF structure probed by SAXS/WAXS

Gas sorption measurements revealed strong suppression of the gas-accessible porosity in Cfilm/ACF samples following carbon film deposition. However, physisorption probes only accessible pores and cannot resolve the structural organization of the carbon network itself.[12, 13] To address this, we employed synchrotron-based small- and wide-angle X-ray scattering (SAXS/WAXS), which provides quantitative insight into pore correlations, structural coherence, and ordering independent of gas accessibility. The SAXS/WAXS data were analyzed using a combination of scattering models to capture the hierarchical and hybrid nature of the samples (**Fig. S9a**):[13]

i. **Debye-Bueche (DB) model** describes scattering from disordered two-phase systems with random heterogeneities, appropriate for the highly porous ACF scaffold:

$$I_{DB}(q) = \frac{8\pi(1-\phi)(\Delta\rho)^2\xi^3}{(1+\xi^2q^2)^2} \tag{1}$$

where ϕ is the porosity fraction, $\Delta\rho$ the scattering length density contrast, and ξ the correlation length describing the average size of density fluctuations

ii. **Teubner-Strey (TS) model** is used for bicontinuous two-phase systems, and it captures quasi-periodic correlations that appear in disordered carbons. The reduced correlation function is given as;

$$\gamma(q) = \frac{\sin\left(\frac{2\pi r}{d}\right)}{2\pi r/d} \exp\left(-\frac{r}{\xi}\right) \tag{2}$$

with corresponding intensity:

$$I_{TS}(q) = \frac{8\pi\phi(1-\phi)(\Delta\rho)^2\xi^3}{\left(1+\left(\frac{2\pi\xi}{d}\right)^2\right)^2 + \left(1-\left(\frac{2\pi\xi}{d}\right)^2\right)\xi^2q^2 + \xi^4q^4} \tag{3}$$

where d is the repeat distance between domains and ξ the correlation length. In the SAXS region, the TS function describes pore correlations, while in the WAXS region, it captures turbostratic ordering of graphitic layers. Using two separate TS functions thus allows separation of pore-scale correlations from interlayer stacking.

iii. **Power-law** describes the low-q scattering from large-scale interfaces:

$$I(q) \propto q^{-n} \tag{4}$$

For porous carbons with sharp pore-solid boundaries, the Porod slope was fixed at $n = 4$, consistent with classical Porod's behavior for disordered carbons.

Both normalized and non-normalized Kratky plots of the scattering profiles were analyzed. The normalized fits, shown in the main manuscript, emphasize relative differences in ordering between samples, while the non-normalized fits presented here (**Figs. S9b-d**) reveal the intensity variations and thereby reflect the extent of structural suppression or enhancement following Cfilm deposition. Together, these complementary representations provide a complete picture of how the coating modifies the ACF scaffold.

The extracted parameters (**Fig. 2g, main**) highlight a pronounced structural transformation. In pristine ACF, the apparent pore size is calculated to be around 0.83 nm. After Cfilm deposition, the pore size contracts to 0.57-0.68 nm, an effect that arises not only from confinement at the external pore mouths but also from partial coverage or filling of internal pores. In this way, both true narrowing and effective blocking of voids contribute to the apparent size reduction. Interestingly, the non-normalized SAXS data reveal an increase in pore scattering intensity after coating for the $\mathrm{Cfilm_{PYR}}$ sample, which can be rationalized as the formation of additional micropore contrast when larger voids are only partially sealed, effectively transforming them into smaller pores.

Alongside pore contraction, the domain spacing derived from TS fits expands from 2.7 nm in pristine ACF to nearly 3.6 nm in coated samples. This increase indicates that the carbon films reorganize the disordered pore framework into larger, more coherent domains, consistent with the smoothing of fiber surfaces. The correlation length follows the same trend, increasing from 0.55 nm to 0.63 nm after coating, which confirms that structural coherence is extended within the newly formed Cfilm shell. The WAXS analysis of the C(002) feature shows a contraction of the interlayer spacing from 0.398 nm in pristine ACF

to 0.379-0.385 nm in Cfilm/ACF, reflecting the emergence of turbostratic ordering. The extent of contraction depends on precursor chemistry: $Cfilm_{PYR}$/ACF achieves slightly tighter stacking than $Cfilm_{HMF}$/ACF and $Cfilm_{THP}$/ACF.

To further isolate the role of coating extent, a thickness study was conducted using HMF as a model precursor (**Fig. S10**). Normalized Kratky fits of DB and TS models (**Fig. S10a-c**) demonstrated that structural changes are obvious. Correlation length increased steadily with thickness, indicating extended structural coherence in the thickest coatings (**Fig. S10d**). Domain spacing likewise grew from 2.7 nm to 3.5 nm, reflecting the evolution of larger Cfilm domains (**Fig. S10e**). The interlayer spacing decreased from 0.398 nm in pristine ACF to 0.385 nm in thick Cfilm/ACF (**Fig. S10f**), while the DB pore size decreased to 0.64 nm, highlighting the combined effects of confinement and pore filling (**Fig. S10g**).

Overall, the precursor- and thickness-dependent SAXS/WAXS highlight two complementary ways in which the Cfilm structure can be tuned. The choice of precursor sets the starting chemistry and baseline order of the carbon film. For example, the heteroatoms can reshape the electronic properties. The coating thickness then determines the extent of structural evolution, with thicker films suppressing microporosity and enhancing ordering. Importantly, neither parameter alone is sufficient: for practical applications, an optimum precursor chemistry and an optimum thickness are needed. Too thin a film may leave the porous scaffold uncovered and inhomogeneous, while overly thick coatings may eliminate useful porosity, especially required for diffusion-controlled ion storage mechanisms.

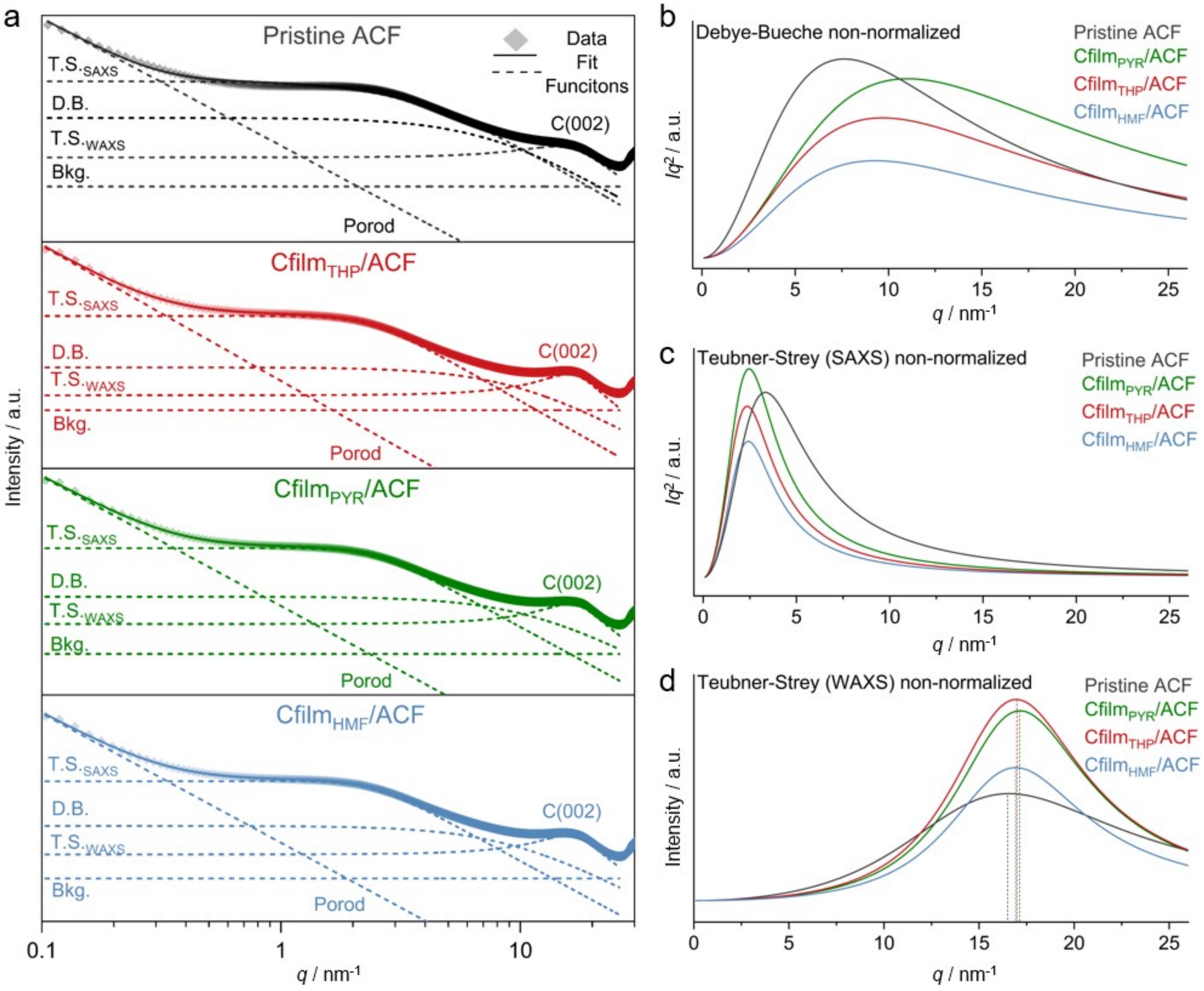

Fig. S9. Structural analysis of pristine ACF and Cfilm/ACF via SAXS and WAXS. **(a)** Representative SAXS and WAXS profiles fitted using a combination of Power Law (Porod region), Teubner-Strey (T.S.), Debye-Bueche (D.B.), and background functions. **(b)** Non-normalized Debye-Bueche fits (as Kratky plot) of the SAXS profiles, highlighting changes in the microporous structure upon film deposition. **(c)** Non-normalized Teubner-Strey fits (as Kratky plot) in the SAXS region are used to extract correlation lengths and domain spacings. **(d)** Non-normalized Teubner-Strey fits (as Kratky plot) in the WAXS region showing peak shifts toward higher q-values in Cfilm/ACF samples, indicating a reduction in the interlayer spacing of the C(002) planes due to the carbon film coating.

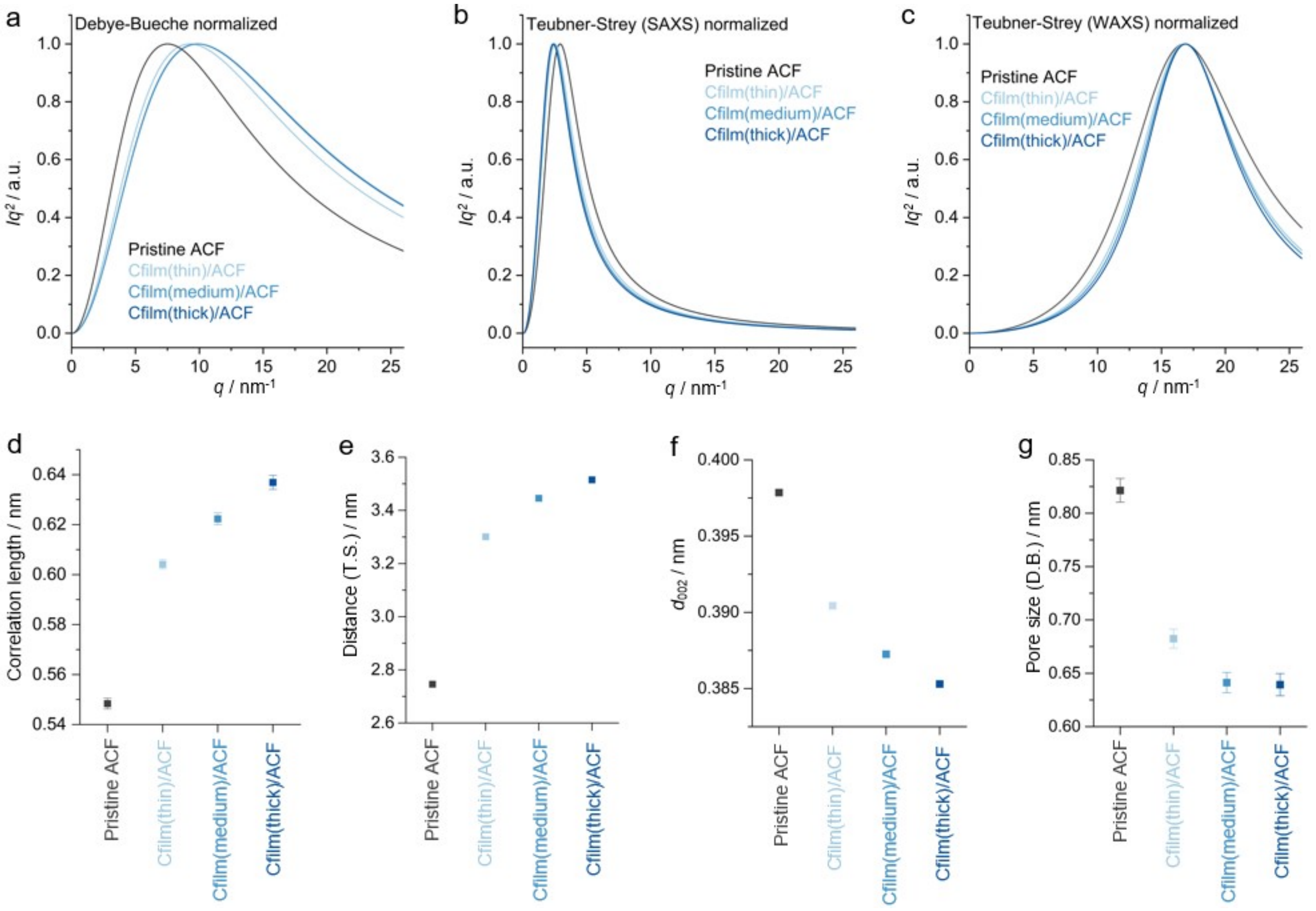

Fig. S10. Effect of carbon film thickness on the structural properties, using 5-(hydroxymethyl)furan-2-carbaldehyde (HMF) as the model precursor. **(a-c)** Normalized Kratky plots of **(a)** Debye-Bueche, **(b)** Teubner-Strey (SAXS), and **(c)** Teubner-Strey (WAXS) model fits, showing systematic changes in structure with increasing Cfilm thickness (thin, medium, thick). **(d-g)** Quantitative structural parameters extracted from SAXS/WAXS model fits, including **(d)** correlation length, **(e)** domain spacing (T.S.), **(f)** interlayer spacing (d_{002}), and **(g)** Debye-Bueche pore size.

Supplementary Note 8. Surface chemistry of Cfilms revealed by XPS

X-ray photoelectron spectroscopy (XPS) was used to probe the surface chemistry of pristine ACF and Cfilm/ACF samples, revealing how precursor chemistry shapes the elemental composition and functional groups of the deposited films. The survey spectra (**Fig. S11a-d**) show dominant C1s and O1s peaks across all samples, along with precursor-specific heteroatom signatures: $Cfilm_{THP}$/ACF exhibits S2p contributions, $Cfilm_{PYR}$/ACF contains N1s features, while $Cfilm_{HMF}$/ACF shows only carbon and oxygen signals. The elemental composition analysis (**Fig. 2h, main**) confirms that pristine ACF already possesses significant oxygen content, originating from surface groups generated during its activation. Importantly, since XPS is a highly surface-sensitive technique and the carbon film thickness is more than 10 nm, the signals obtained for Cfilm/ACF samples originate exclusively from the deposited carbon film rather than the underlying ACF scaffold. The detailed deconvolution of S2p for $Cfilm_{THP}$/ACF and N1s for $Cfilm_{PYR}$/ACF is provided in the main manuscript (**Fig. 2i, j**).

C1s spectra (**Fig. S11e**) provide further insight into bonding environments. In principle, well-ordered graphitic carbon exhibits an asymmetric C1s line shape due to its delocalized π-electron system, and under such conditions, it is common to distinguish sp^2 (asymmetric) and sp^3 (symmetric) carbon components. However, for graphitic-like or highly disordered carbons with a relatively low degree of long-range order, reliable separation of sp^2 and sp^3 contributions becomes challenging. Consequently, sp^2 and sp^3 carbon were treated as a single C-C/C=C component under these conditions. All samples exhibit a dominant sp^2 C-C/C=C component at 284.6 eV. Other contributions appear at higher binding energies, including C-O (ca. 285.9 eV), C=O (ca. 287.1 eV), O-C=O (ca. 288.4 eV), and π-π^* (ca. 290.5 eV). The O1s spectra (**Fig. S11f**) corroborate these findings.

These results show that pristine ACF already has a strongly oxygenated surface. As a result, each film develops a distinct surface character, with sulfur-, nitrogen-, or oxygen-rich groups expressed in different ways. This chemical tunability is important because it can directly affect material properties such as electrochemical stability. This difference has previously been confirmed by the work function values.

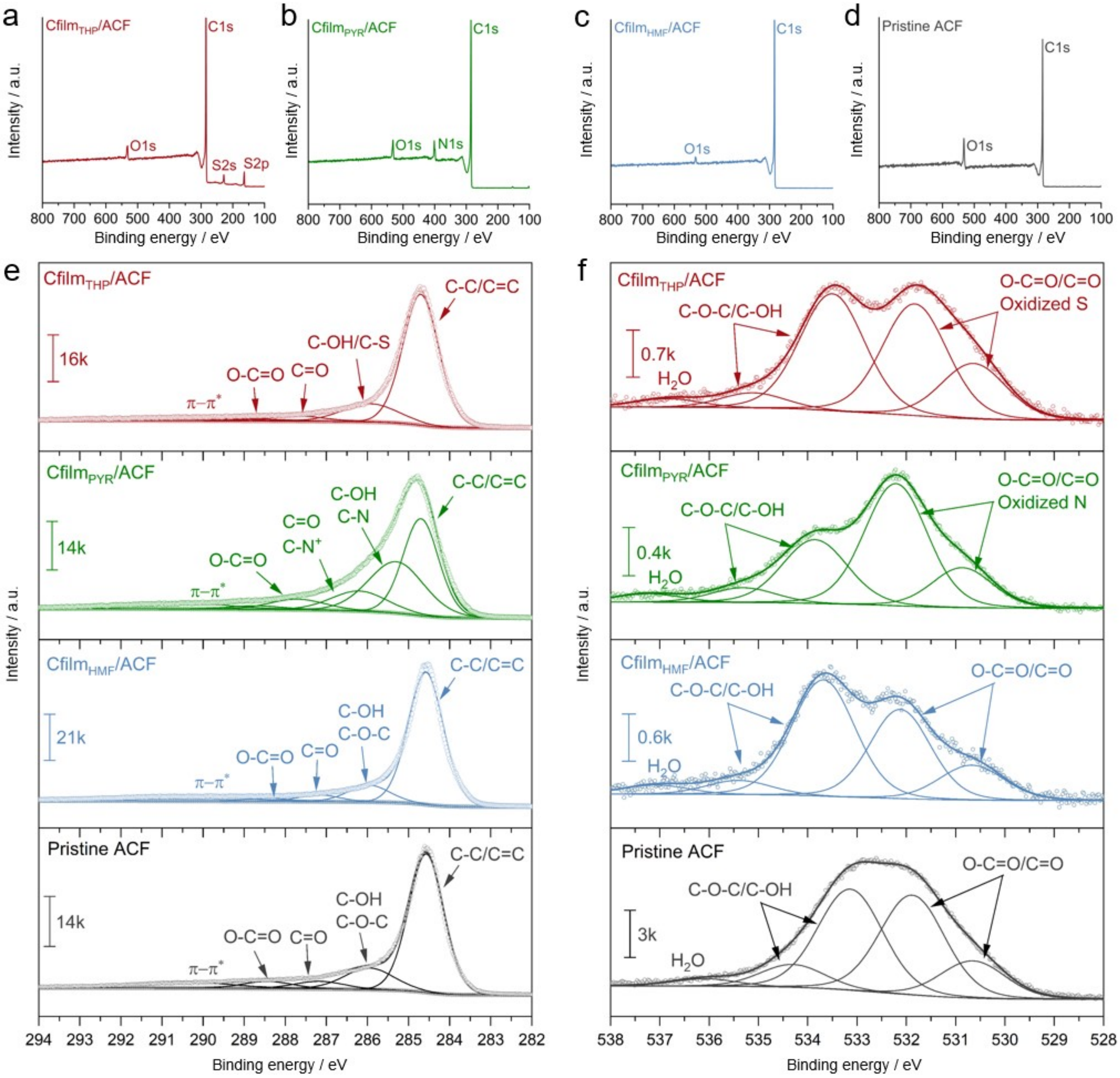

Fig. S11. XPS analysis of pristine ACF and Cfilm/ACF samples. **(a-d)** Survey spectra for Cfilm$_{THP}$/ACF (red), Cfilm$_{PYR}$/ACF (green), Cfilm$_{HMF}$/ACF (blue), and pristine ACF (black) showing the presence of C1s, O1s, and heteroatoms (S2p for Cfilm$_{THP}$/ACF, N1s for Cfilm$_{PYR}$/ACF). **(e)** High-resolution C1s spectra reveal differences in surface functionalities across the samples, with fitted components. **(f)** High-resolution O1s spectra showing varying degrees of oxidation. Functional group differences reflect precursor identity and carbon film composition.

Supplementary Note 9. Sheet resistance and electrical conductivity of Cfilm/SiO_2 and Cfilm/ACF samples

Sheet resistance and bulk conductivity of the Cfilms and Cfilm/ACF composites were evaluated using a four-point probe method (**Table S1**). On planar SiO_2 wafers, the $Cfilm_{PYR}$ film exhibited the lowest sheet resistance, followed by $Cfilm_{HMF}$ and $Cfilm_{THP}$, indicating that the heteroatom chemistry significantly affects the electronic conductivity. A similar trend was observed for Cfilm/ACF samples, where the bulk conductivity of $Cfilm_{PYR}$/ACF greatly exceeds that of $Cfilm_{HMF}$/ACF, $Cfilm_{THP}$/ACF, and pristine ACF. These results demonstrate that the Cfilm coating not only significantly enhances the electrical conductivity of the ACF scaffold but also enables tunability through precursor chemistry.

Table S1. Sheet resistance of Cfilms on SiO_2 and bulk conductivity of Cfilm/ACF samples.

**Cfilm/SiO_2 samples**	**Sheet resistance / Ohm sq^{-1}**
$Cfilm_{HMF}$	650
$Cfilm_{THP}$	1150
$Cfilm_{PYR}$	540
**Cfilm/ACF samples**	**Conductivity / kS m^{-1}**
Pristine ACF	0.25
$Cfilm_{HMF}$/ACF	1.5
$Cfilm_{THP}$/ACF	1.2
$Cfilm_{PYR}$/ACF	3.3

Supplementary Note 10. Electrochemical kinetics from CV and *b*-value analysis

Cyclic voltammetry (CV) analysis provides insight into the charge storage mechanism of pristine ACF and Cfilm/ACF electrodes. CV curves were recorded at scan rates between 0.05 and 2.0 mV s^{-1} (**Fig. S12a-d**). To quantify the storage mechanism, the relationship between peak current (i) and scan rate (v) was analyzed according to the power law:

$$i = av^b \quad (5)$$

where a is a constant and b is the slope obtained from the log(i)-log(v) plots (insets of **Fig. S12**). A b-value of 1 indicates surface-controlled capacitive processes, while a value of 0.5 corresponds to semi-infinite diffusion-limited behavior, characteristic of diffusion-driven storage.[14] Intermediate values reflect a mixed contribution from both mechanisms.

Pristine ACF shows quasi-rectangular CV profiles but also exhibits pronounced redox features at low potentials, indicating that pseudocapacitive contributions from surface functional groups may coexist with double-layer adsorption. The extracted b-value of 0.88 supports a surface-controlled mechanism, though the presence of redox peaks suggests an additional faradaic contribution.

In contrast, all Cfilm/ACF electrodes ($Cfilm_{HMF}$/ACF, $Cfilm_{PYR}$/ACF, and $Cfilm_{THP}$/ACF) exhibit CV shapes with suppressed current response, reflecting the reduced accessible surface area after Cfilm deposition. Their b-values, ranging from 0.50 to 0.53, are much closer to 0.5, indicating that sodium storage becomes strongly diffusion-limited. This transition arises from the conformal Cfilm coating, which confines porosity and alters ion transport pathways within the ACF scaffold.

These findings are consistent with the gas sorption and SAXS/WAXS results, both of which demonstrated suppression of micropores and narrowing of pore size after Cfilm deposition. Together, the CV analysis confirms that pristine ACF behaves as a capacitive material with additional pseudocapacitive contributions, while Cfilm/ACF hybrids transition toward a diffusion-controlled storage regime dictated by the carbon film.

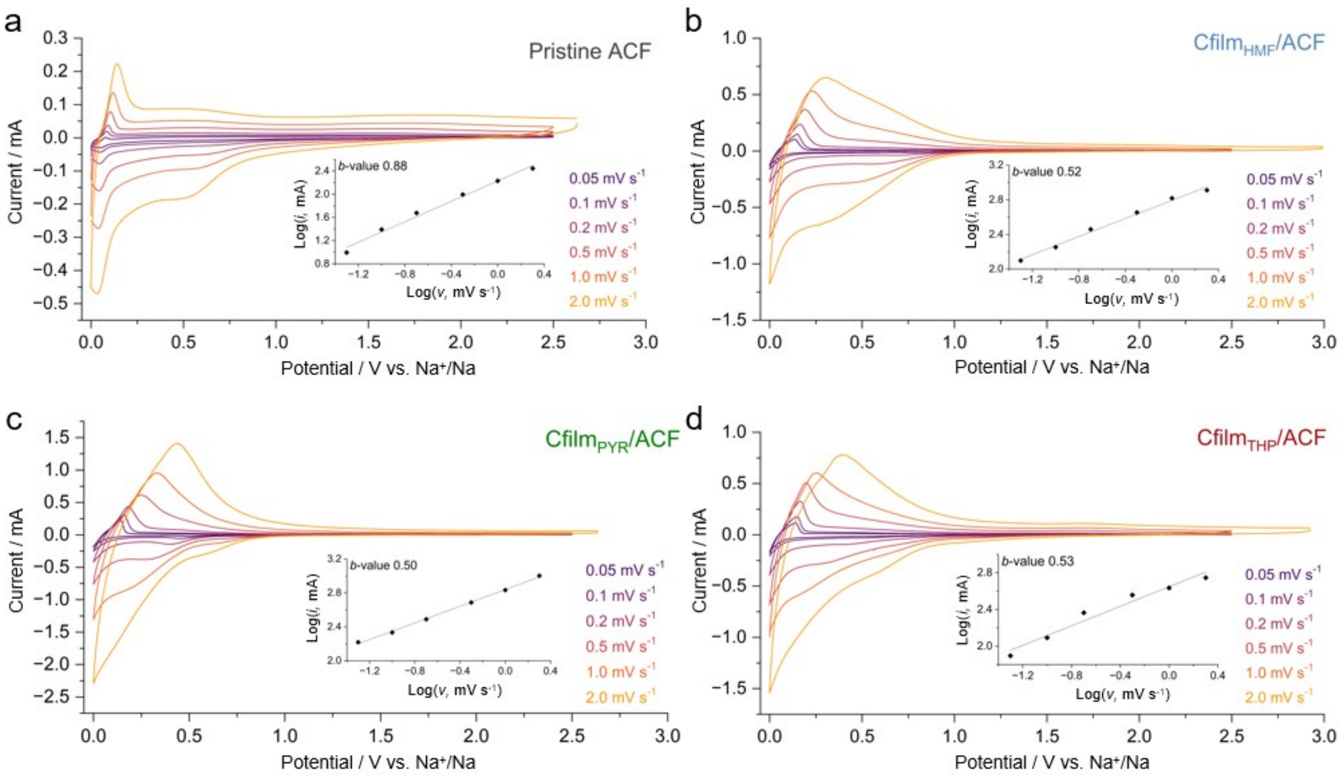

Fig. S12. CV analysis of pristine ACF and Cfilm/ACF electrodes at various scan rates (0.05-2.0 mV s^{-1}) in half-cells. CV curves for **(a)** pristine ACF, **(b)** Cfilm$_{HMF}$/ACF, **(c)** Cfilm$_{PYR}$/ACF, and **(d)** Cfilm$_{THP}$/ACF. The corresponding insets show the log(i)-log(v) plots used to extract the b-values, which help distinguish between surface- and diffusion-controlled charge storage behavior.

Supplementary Note 11. Morphological and chemical analysis of the SEI via SEM and XPS

To gain insight into the morphological and chemical nature of the SEI, SEM and XPS depth-profiling were performed on pristine ACF and the Cfilm/ACF samples. The electrodes investigated here are self-standing (woven-ACF), eliminating any contributions from binders or conductive carbon additives and thus enabling a reliable assessment of the SEI properties. The electrodes were recovered after the initial cycle, carefully disassembled in a glovebox, and washed multiple times with DEC to remove residual electrolyte species before being transferred to the SEM and XPS facilities using a sealed transfer setup.

SEM images (**Fig. S13**), including both low- and high-magnification views, reveal pronounced differences in SEI morphology. The pristine ACF exhibits a distinct interphase morphology characterized by branched porous structures that entirely cover the surface and appear to grow in a dendritic manner. This behavior can be associated with the reactive and open porous structure of ACF, which promotes aggressive electrolyte decomposition and interphase growth. In contrast, the Cfilm/ACF electrodes reveal significantly different morphologies. The $Cfilm_{PYR}$/ACF surface is uniformly covered by a continuous layer, with no evidence of the dendritic or flake-like features observed for pristine ACF. This homogeneous layer suggests more controlled interfacial reactions and a stabilized SEI formation. Minor contrast variations in localized regions may indicate compositional heterogeneity, as found in a mosaic-type SEI. For the $Cfilm_{HMF}$/ACF, the mosaic-like morphology here is much more pronounced. The surface consists of interconnected nanoparticles embedded within a continuous, polymer-like matrix. This dual-phase structure could refer to the coexistence of inorganic/organic SEI components, where the particulate domains correspond to inorganic species, while the surrounding matrix represents organic decomposition products. The $Cfilm_{THP}$/ACF sample shows a similar morphology but more heterogeneity compared to the $Cfilm_{HMF}$/ACF. The SEI appears less uniform, with irregularly distributed domains and a more fragmented surface structure. This observation correlates with its lower ICE. A possible explanation lies in the high concentration of sulfur- and oxygen-containing functional groups within the $Cfilm_{THP}$. In particular, sulfur sites are known to be highly sodiophilic and can promote additional sodium adsorption at higher potentials (~1.0 V), as evidenced by the SEI_i cathodic peak in the CV profile and corroborated by DFT calculations from our previous study.[15] Such enhanced sodium affinity can further catalyze electrolyte decomposition, leading to excessive and less controlled SEI growth.

To further investigate the chemical states within the SEI, XPS depth profiling was performed. By combining sequential low-energy Ar^+ sputtering (500 eV), chemical information was obtained from

different depths across the interphase region.[16] In all samples, the F1s spectra (**Fig. S15**) are dominated by a peak at ~685 eV, corresponding to NaF, constituting a major component of the SEI. A second contribution at ~688.5 eV is assigned to C–F/P–F species, which are hard to separate at this stage.[17] For pristine ACF, depth profiling reveals three distinct fluorine-containing contributions instead of the two main components observed for the Cfilm/ACF samples. Alongside the dominant NaF peak and the broader C–F/P–F contribution, a smaller high-binding-energy feature assigned to $C–F_x$ species is also observed. This contribution is most pronounced at the outermost surface and gradually decreases with sputtering time, while the relative intensity of the C–F/P–F component increases. This behavior suggests that fluorinated organic species are primarily concentrated in the outer SEI layer, whereas fluorophosphate-related decomposition products originating from $NaPF_6$ are distributed deeper within the interphase. This behavior can be correlated with the more aggressive electrolyte decomposition occurring over a wider potential range, as evidenced by multiple cathodic features in the initial CV scan, features that are absent in the Cfilm/ACF electrodes. In contrast, the Cfilm/ACF electrodes exhibit dominant NaF contributions together with comparatively weaker C–F/P–F signals.

Consistent with its lower ICE and more heterogeneous SEI morphology observed by SEM, $Cfilm_{THP}$/ACF exhibits the most distinct interfacial composition among the coated systems. Here, the F1s signal of $Cfilm_{THP}$/ACF is significantly weaker compared to the other electrodes, indicating a lower fraction of fluorinated species (**Fig. S15d**). Although similar NaF features are still observed, the interphase chemistry is clearly dominated by sulfur-, carbon-, and sodium-rich decomposition products. The S2p spectra reveal the presence of inorganic and hybrid sulfur-containing species within the SEI (**Fig. S16b**). These features can originate from irreversible interfacial reactions during the initial cycle, where sulfur-containing functionalities of $Cfilm_{THP}$, including possible thiol- and thiocarbonyl-related groups, participate in electrolyte decomposition and interphase formation. Contributions associated with reduced S species together with oxidized sulfur species, are observed throughout the depth profiling. Since these features are absent in the fresh electrode, the results indicate active involvement of sulfur species in the interfacial reactions and suggest that sulfur-containing compounds are distributed throughout the bulk SEI, contributing to the lower ICE of $Cfilm_{THP}$/ACF.[18]

For $Cfilm_{PYR}$/ACF, the N1s signal remains relatively weak within the SEI region and becomes more pronounced only after longer sputtering (**Fig. S16a**). This indicates that nitrogen species are not significantly incorporated into the SEI but instead mainly originate from the underlying electrode. Such

behavior is consistent with the dominance of graphitic-N in the $Cfilm_{PYR}$, which are electronically stabilized and less prone to participate in parasitic interfacial reactions.

Further insight can be obtained from the C1s spectra (**Fig. S14**). In all electrodes, pronounced high-binding-energy contributions around ~289-291 eV are assigned to organic and inorganic carbonate species, including Na_2CO_3 and alkyl carbonate/dicarbonate-type decomposition products (i.e, sodium ethylene dicarbonate (NEDC),[19, 20, 21] which together with NaF constitute dominant components of SEIs formed in carbonate-based electrolytes. Additional contributions between ~285.5 and 288.5 eV originate from oxidized organic carbon species such as C–O, C=O, and O–C=O moieties formed during electrolyte decomposition. While these features are present in all samples, their relative intensities strongly depend on the surface chemistry of the electrode and the resulting interphase composition.

At the outermost surface, a distinct carbon signal from the underlying electrode is generally not observed due to the surface-sensitive nature of XPS. However, upon depth profiling, the gradual emergence of a C–C/C=C contribution around ~284.5 eV provides indirect information regarding the relative SEI thickness. This behavior is most pronounced for $Cfilm_{HMF}$/ACF, where the carbon contribution becomes visible after only a few sputtering cycles, indicating the formation of a relatively thin SEI layer. In contrast, for $Cfilm_{THP}$/ACF and especially $Cfilm_{PYR}$/ACF, the C–C/C=C signal emerges only at later sputtering stages, suggesting comparatively thicker interphases. In particular, $Cfilm_{PYR}$/ACF exhibits an extended SEI formation plateau around ~0.5 V during the initial sodiation process, which may promote prolonged electrolyte decomposition and consequently the formation of a thicker interphase. $Cfilm_{THP}$/ACF exhibits the strongest Na_2CO_3-related contribution relative to the C–C/C=C signal, suggesting that the inorganic fraction of the SEI is mainly composed of carbonate species together with sodium sulfides/sulfates and oxides. Although NaF is still detected, its relative contribution is significantly weaker. Considering the critical role of NaF in forming mechanically robust and ionically conductive SEIs, the reduced NaF content together with the dominance of sulfur-rich interphase may contribute to less favorable Na^+ transport and partially explain the comparatively inferior electrochemical performance observed for $Cfilm_{THP}$/ACF. Pristine ACF does not reveal the characteristic C–C/C=C features of the underlying electrode even after extended sputtering, indicating the presence of a substantially thicker interphase. Furthermore, unlike the coated electrodes where C–O-related species dominate the oxidized carbon contributions, pristine ACF exhibits a particularly strong C=O component together with pronounced $C–F_x$ features, which are also reflected in the F1s spectra. These observations indicate a chemically distinct interphase dominated by extensive electrolyte decomposition and fluorinated organic decomposition

products formed on the highly reactive open porous carbon surface. The resulting thick and heterogeneous SEI is consistent with the low ICE and the aggressive dendritic interphase morphology observed in SEM.

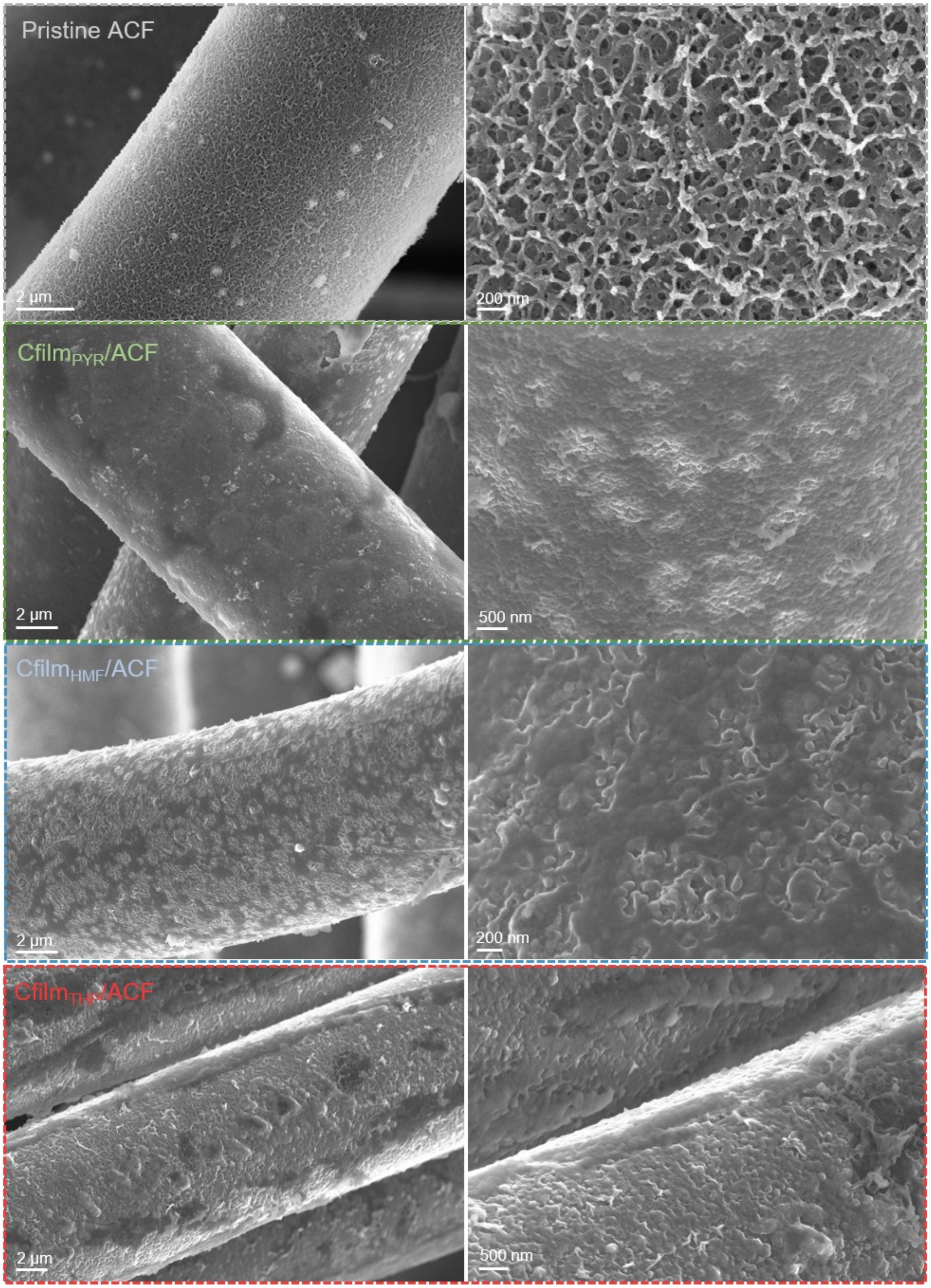

Fig. S13. SEM images of the pristine ACF and Cfilm$_{PYR}$/ACF, Cfilm$_{HMF}$/ACF, and Cfilm$_{THP}$/ACF after the initial cycle, showing the influence of carbon film chemistry on the morphology of the passivation layer.

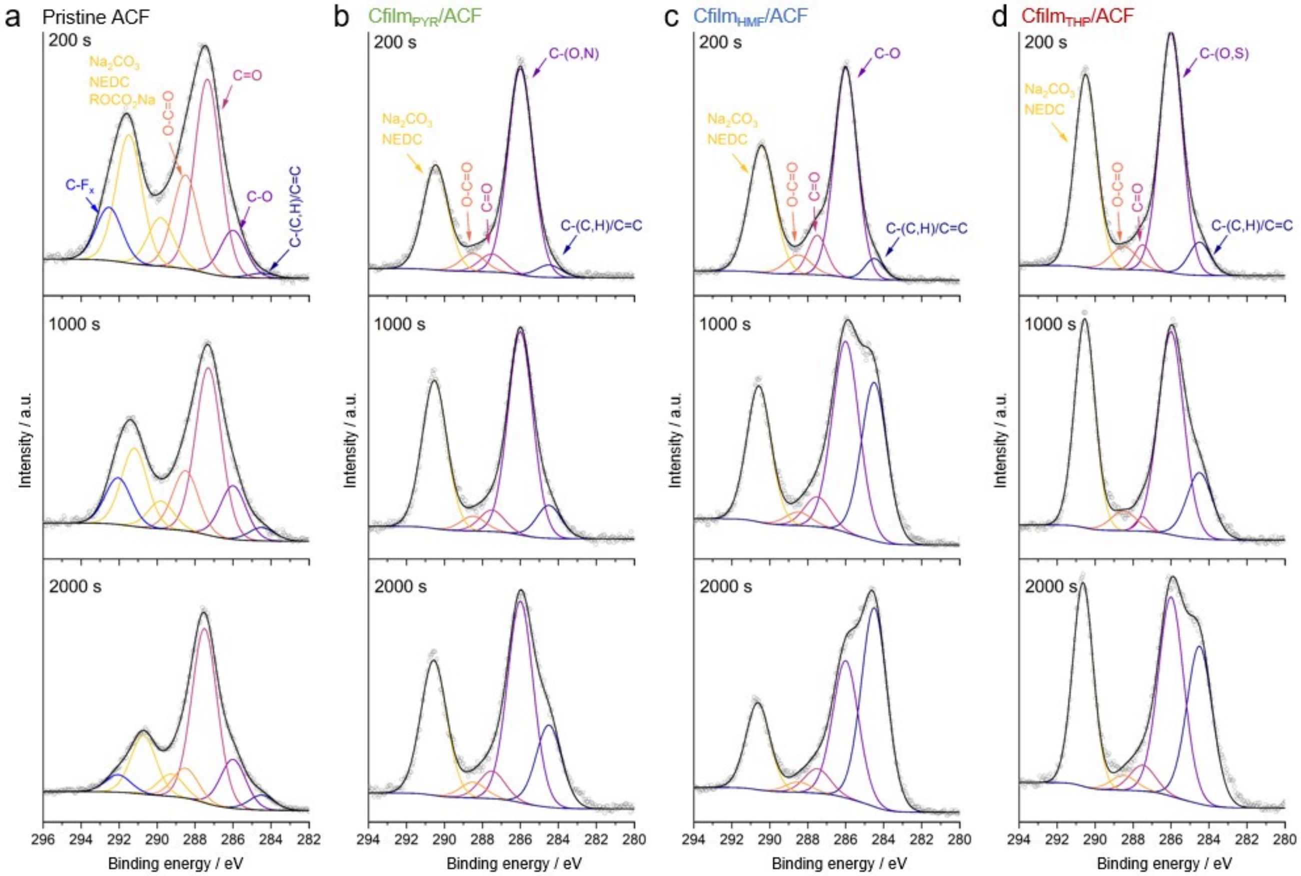

Fig. S14. C1s XPS depth profiling spectra of **(a)** pristine ACF, **(b)** Cfilm$_{PYR}$/ACF, **(c)** Cfilm$_{HMF}$/ACF, and **(d)** Cfilm$_{THP}$/ACF electrodes collected after different sputtering times. The spectra reveal differences in SEI composition and thickness depending on the surface chemistry of the carbon film, including variations in inorganic and organic carbonate species, fluorinated decomposition products, and the gradual emergence of the underlying carbon signal during depth profiling.

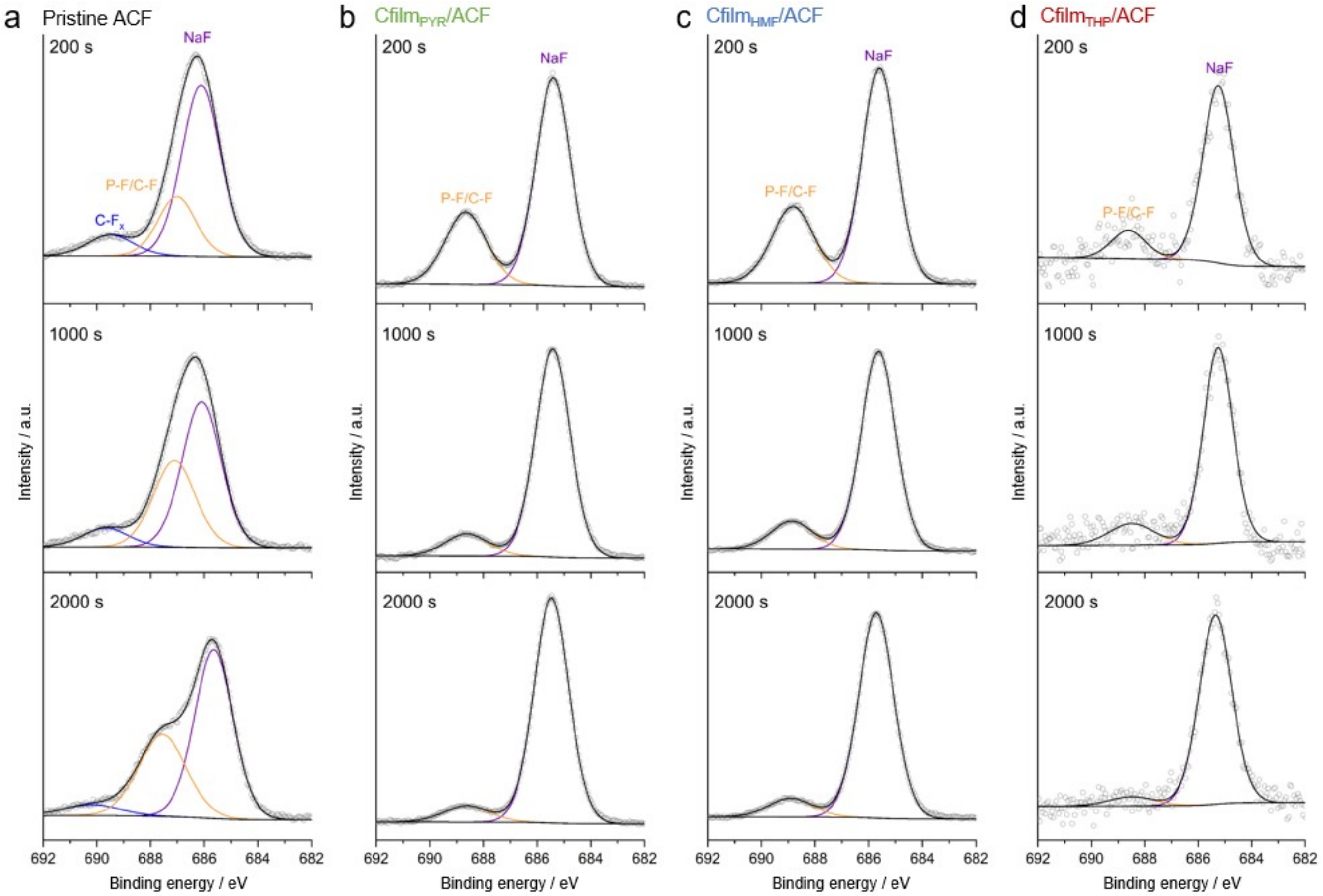

Fig. S15. F1s XPS depth profiling spectra of **(a)** pristine ACF, **(b)** Cfilm$_{PYR}$/ACF, **(c)** Cfilm$_{HMF}$/ACF, and **(d)** Cfilm$_{THP}$/ACF electrodes collected after different sputtering times. All electrodes show dominant NaF within the SEI, while pristine ACF additionally exhibits pronounced fluorophosphate contributions associated with extensive $NaPF_6$ decomposition.

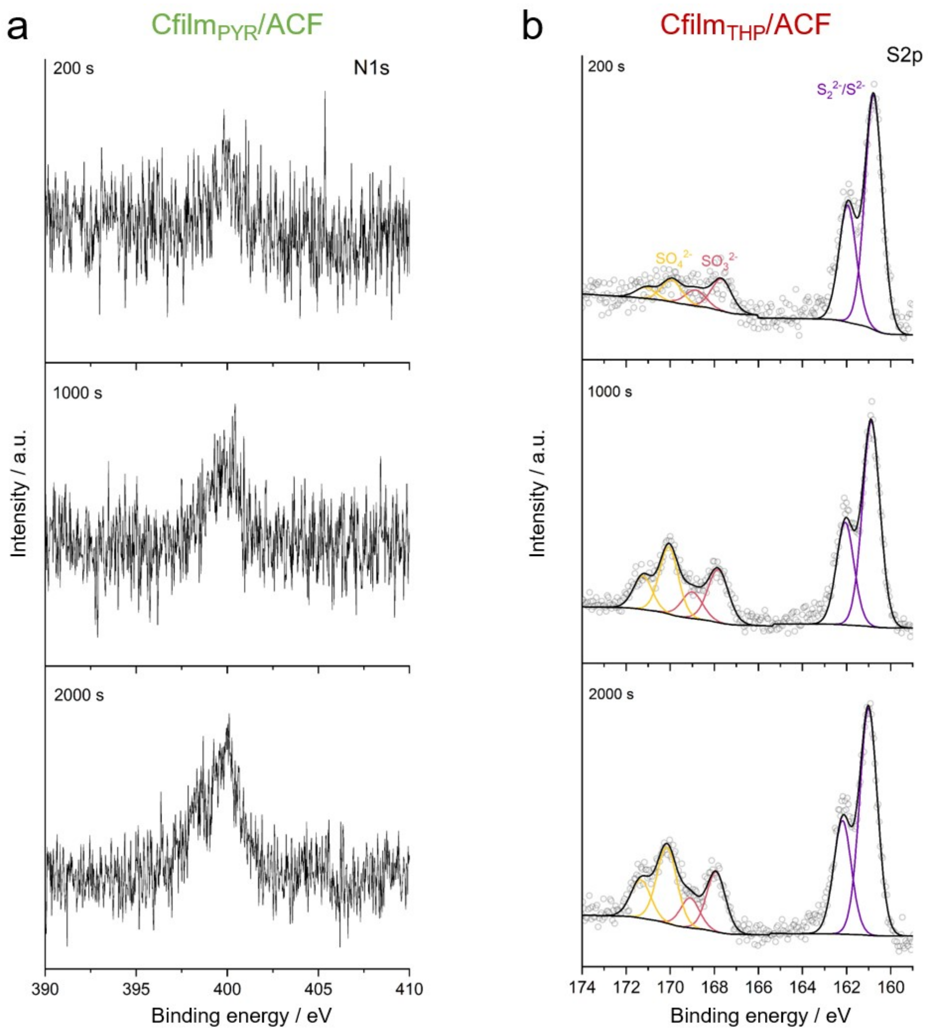

Fig. S16. (a) N1s and **(b)** S2p XPS depth profiling spectra of cycled $Cfilm_{PYR}$/ACF and $Cfilm_{THP}$/ACF electrodes collected after different sputtering times. $Cfilm_{PYR}$/ACF shows only weak N1s contributions, mainly originating from the underlying electrode, whereas $Cfilm_{THP}$/ACF exhibits pronounced sulfur-containing SEI species, confirming the active involvement of sulfur functionalities in interphase formation.

Supplementary Note 12. *In-situ* EIS of interfacial processes in pristine and Cfilm/ACF electrodes

To further probe the charge transfer and interfacial processes in pristine ACF and Cfilm/ACF electrodes, *in-situ* EIS measurements were carried out during the initial sodiation, with Nyquist plots recorded at selected potentials (**Fig. S17a-d**). These measurements provide insight into how Cfilms influence interfacial resistance and ion transport across different states of sodiation.[22, 23] The Nyquist plots were analyzed using an elliptical fitting approach, which provides a more realistic representation of the distorted semicircle features observed in porous carbons.[24] This method was chosen because the impedance response of such electrodes deviates from the idealized semicircle, reflecting distributed resistances and capacitances within the porous network. Importantly, we did not include a Warburg tail in the fitting, as the characteristic low-frequency 45° diffusion feature can deviate in these materials. This suppression arises because sodium-ion transport in such systems is dominated by rapid access to surface sites and confined pore regions, rather than long-range bulk diffusion. As a result, the impedance response is better captured by elliptical elements corresponding to charge-transfer and interfacial processes. The extracted fitting parameters are summarized in **Table S2**, based on the equivalent circuit model consisting of series resistance (R_s), charge-transfer resistance (R_{ct}), and a passivation resistance (R_p), the latter associated with SEI-related impedance. Across all systems, the R_s remains very similar, confirming good comparability between the different electrodes.

For pristine ACF (**Fig. S17a, Table S2**), the impedance spectra reveal distinct changes as a function of potential. At high potentials (3.0-2.0 V (vs. Na^+/Na, unless otherwise stated)), the Nyquist plots show relatively moderate semicircles, with R_{ct} values on the order of 110-135 Ohm cm^2. These values remain nearly unchanged between 3.0 and 2.0 V, suggesting that no major processes are taking place in this potential range. Instead, the processes active here are most likely associated with sluggish reactions involving oxygen-rich surface functional groups and the electrolyte accessible surface area. This interpretation is supported by the presence of broad features in the CV curves of pristine ACF (**Fig. 3d, main**), which are absent in the Cfilm/ACF electrodes. Since no second semicircle is observed, these processes do not correspond to a passivation layer but rather to parasitic side reactions characteristic of the oxygenated surfaces.

A more significant change appears at 1.0 V, where a double semicircle emerges due to the onset of passivation layer growth. At this potential, R_{ct} increases further, consistent with SEI formation beginning at the surface.[1] The growth of this resistive interphase increases the kinetic barrier, reflected in the larger

semicircle. Moving to 0.7 V, the impedance rises sharply. This dramatic increase corresponds directly to the SEI-related potentials around 0.7 and 0.5 V, observed also in the initial CV scan. At these potentials, the electrolyte decomposition driving SEI formation is kinetically hindered, far from equilibrium, and associated with very low exchange current density. At the same time, the developing SEI blocks active surface sites and forms an electronically insulating barrier, further restricting charge transfer. By 0.5 V, although SEI growth continues, R_{ct} decreases slightly compared to the extreme peak at 0.7 V. This behavior reflects the effect of increasing cathodic polarization, whereby the R_{ct} decreases. In this regime, sodium ions are accommodated within the carbon scaffold, and the established SEI allows for improved charge transfer. The decrease in R_p is potential-driven and reflects reduced interfacial resistance.

Switching to the Cfilm/ACF (**Fig. S17b-d, Table S2**), the general behavior observed in pristine ACF is maintained, although with precursor-dependent modifications. Since there is no major electrochemically driven process between 3 and 1 V, the impedance at 2 V was excluded for the Cfilm samples. At 3 and 1 V, the impedance response is broadly similar across all Cfilms, indicating relatively inert behavior in this potential region. As the potential decreases toward 0.7 V and subsequently 0.5 V, where SEI formation begins, a second semicircle appears in the Nyquist plots. This feature is associated with interphase formation. With further sodiation, the SEI continues to stabilize, and R_{ct} gradually decreases due to cathodic polarization. As discussed above, this relaxation reflects the transition from a kinetically hindered interphase to a stabilized SEI that permits ion transport. At lower potentials, sodium insertion into the porous scaffold further improves ionic pathways, reducing both R_p and R_{ct} in a manner consistent with the behavior already described for pristine ACF.

One interesting feature at 0 V is the slight increase in impedance observed for the Cfilm/ACF samples. This rise is mainly linked to the R_p, rather than the R_{ct}, which actually decreases due to polarization. A possible explanation lies in electrolyte depletion within confined pore regions. As sodium fills the micropores and forms clusters at near-0 V, the local electrolyte environment becomes depleted in free solvent and mobile ions. This reduces ionic conductivity across the already-formed SEI and within the interfacial region, making ion transport less efficient. Importantly, this effect does not indicate further SEI thickening; rather, it reflects a potential-dependent change in interfacial permeability.

Table S2. Equivalent circuit fitting parameters extracted from *in-situ* EIS.

	**Pristine ACF**	**$Cfilm_{HMF}$/ACF**	**$Cfilm_{PYR}$/ACF**	**$Cfilm_{THP}$/ACF**
Potential (V vs. Na^+/Na)	R_s - R_{ct} - R_p (Ohm cm^2)			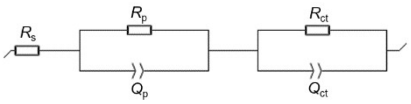
**3.0**	1.2 - 135.0 - n/a	1.4 - 39.7 - n/a	1.5 - 141.3 - n/a	1.1 - 14.9 - n/a
**2.0**	1.2 - 110.5 - n/a	-	-	-
**1.0**	1.3 - 156.9 - 68.2	1.7 - 36.5 - n/a	1.6 - 137.0 - n/a	1.3 - 7.4 - n/a
**0.7**	1.0 - 3512.8 - 124.2	2.0 - 771.8 - 15.1	1.5 - 1288.8 - 99.2	1.3 - 137.9 - 3.8
**0.5**	1.2 - 1065.7 - 75.3	2.0 - 369.9 - 10.9	2.0 - 272.3 - 37.7	1.3 - 62.2 - 3.0
**0.2**	1.4 - 198.8 - 22.3	2.0 - 143.2 - 5.3	2.0 - 48.3 - 12.0	1.3 - 17.7 - 3.8
**0**	1.4 - 73.9 - 8.4	2.0 - 29.7 - 6.9	2.0 - 37.9 - 16.8	1.3 - 11.0 - 9.1

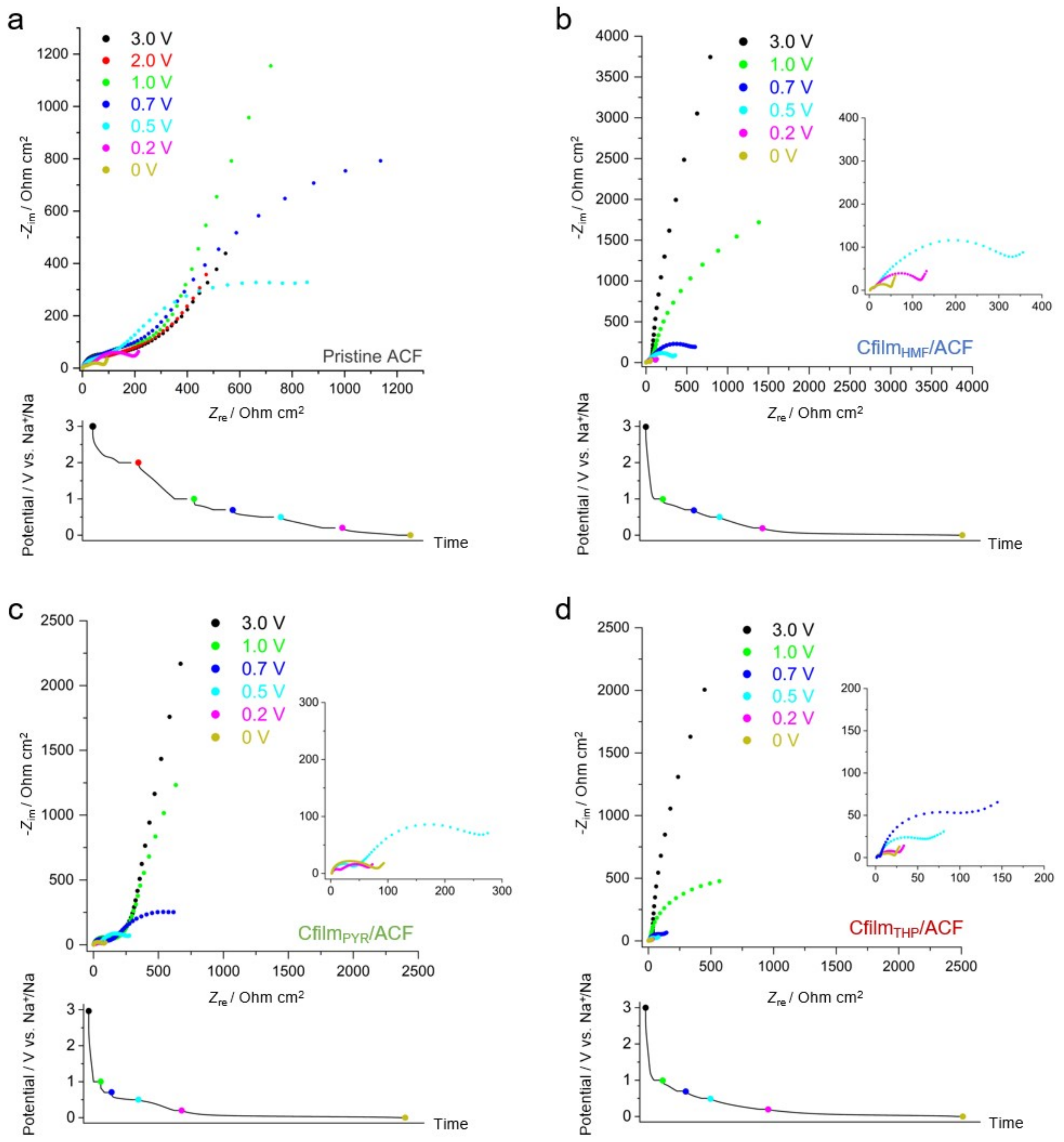

Fig. S17. *In-situ* EIS of pristine ACF and Cfilm/ACF electrodes during initial sodiation. **(a-d)** Nyquist plots recorded at selected potentials for **(a)** pristine ACF, **(b)** Cfilm$_{HMF}$/ACF, **(c)** Cfilm$_{PYR}$/ACF, and **(d)** Cfilm$_{THP}$/ACF. Insets provide close-up views of the low-impedance region at lower potentials. Lower panels show the corresponding discharge profiles, where a constant voltage was applied at each marked potential to ensure stable EIS acquisition.

Supplementary Note 13. DRT analysis of charge-transfer and diffusion processes during sodiation

To gain deeper insight into the electrochemical processes beyond what conventional EIS fitting provides, the distribution of relaxation times (DRT) method was applied to impedance spectra of the electrodes at various states of charge. The DRT approach works by deconvoluting the total impedance response into a continuous distribution of elementary relaxation processes, each characterized by its own time constant. This allows visualization of distinct electrochemical contributions, such as contact resistance, ion transport through the SEI, charge-transfer kinetics, and solid-state diffusion, without assuming a predefined equivalent circuit.[25, 26, 27]

Mathematically, the impedance is expressed as an integral of distributed Voigt elements over a logarithmic timescale, with Tikhonov regularization applied to solve the inherently ill-posed inversion problem. The resulting DRT spectrum consists of Gaussian-like peaks, each corresponding to a specific physical process. As established in literature, high-frequency peaks are typically associated with contact and electronic resistance, mid-frequency features correspond to an interfacial ion transport, and low-frequency peaks relate to charge-transfer and solid-state diffusion processes. Importantly, varying the state of charge (SOC) provides a way to identify the origin of each relaxation process, as their evolution reflects changes in ionic transport and interphase dynamics during cycling.[25, 26, 27]

Compared to conventional EIS, DRT provides interpretability for overlapping processes, particularly relevant for complex carbon-based electrodes where surface reactions, SEI growth, and sodium ion diffusion occur concurrently. In our system, this approach enables tracking how the Cfilm coating modulates each of these relaxation regions and helps decouple the electronic and ionic contributions arising from the carbon film and underlying ACF scaffold.

Fig. S18 presents the DRT spectra of pristine ACF and Cfilm/ACF electrodes obtained at selected potentials during the first discharge. In all cases, three distinct relaxation regions can be identified, corresponding to high-, medium-, and low-frequency processes. These are denoted as P_1 ($\tau > \approx 10^0$ s), P_2 ($\approx 10^{-2} < \tau < \approx 10^0$ s), and P_3 ($\approx 10^{-4} < \tau < \approx 10^{-2}$ s). The process P_1 is attributed to solid-state sodium diffusion within the carbon matrix, reflecting ion transport through the bulk of the porous network. The P_2 process, located at intermediate frequencies, corresponds to the charge-transfer resistance, governing the kinetics of sodiation and desodiation. The component P_3 is assigned to interfacial resistance phenomena, mainly

related to the passivation layer, where sodium ion desolvation and migration through the interphase potentially dominate.[26, 28]

To ensure that the observed relaxation processes originate from the carbon electrodes rather than from the sodium counter electrode, a symmetric Na-Na cell was assembled as a control (**Fig. S18a, inset**). In this system, only two peaks were detected in the high time constant region, primarily corresponding to contact and electronic resistance contributions. No distinguishable activity was observed in the medium or low-frequency ranges, confirming the absence of solid-state diffusion or charge-transfer processes in this symmetric configuration, as expected. Although the main peak appears close to the P_3 range, it is slightly offset (around 10^{-3} s) with a minor shoulder feature. Considering the substantially lower impedance values of the Na-Na cell compared to the carbon electrodes, its contribution to the spectra can be safely neglected.

At 3.0 V (vs. Na^+/Na, unless otherwise stated) (**Fig. S18a**), the DRT spectra are dominated by the P_1 process. No P_2 or P_3 contributions are observed, consistent with the absence of charge-transfer and passivation-related processes at this potential.

At 1.0 V (**Fig. S18b**), the overall impedance related to solid-state diffusion decreases compared to 3.0 V, indicating that ion transport becomes easier. The spectra of all samples appear relatively similar in shape and magnitude, though $Cfilm_{THP}$/ACF shows the lowest total intensity, suggesting that sulfur-containing sites might facilitate sodium-ion adsorption at an earlier stage. In this potential range, a distinct P_2 feature emerges, corresponding to charge-transfer resistance.

At 0.7 V (**Fig. S18c**), the solid-state diffusion peak P_1 for the Cfilm/ACF electrodes is smaller than for pristine ACF, indicating lower diffusion resistance in the coated systems at this stage. The charge-transfer peak P_2 starts to emerge for all samples; among the coated films, $Cfilm_{HMF}$/ACF shows the largest P_2. This potential marks the onset of SEI formation across all electrodes. By 0.5 V (**Fig. S18d**), P_2 and P_1 have comparable intensities, and charge-transfer processes become the dominant contribution. Across the coated electrodes, $Cfilm_{THP}$/ACF still maintains the lowest P_1 and P_2 intensities.

At 0.2 V (**Fig. S18e**), corresponding to the onset of the plateau region, SEI formation is already complete. This is the transition from surface-controlled storage to pore filling and solid-state diffusion-dominated behavior, consistent with the mechanism previously discussed in our earlier study.[29] In this regime, the intensity of P_2 decreases relative to P_1, indicating that ion transport through the carbon network becomes the rate-determining process.

At 0 V (**Fig. S18f**), when the electrodes reach full sodiation, the micropores are nearly saturated. The P_1 intensity at 0 V remains lowest for $Cfilm_{PYR}$/ACF and $Cfilm_{THP}$/ACF, whereas $Cfilm_{HMF}$/ACF and pristine ACF show comparable and higher P_1 values, reflecting slightly increased diffusion impedance.

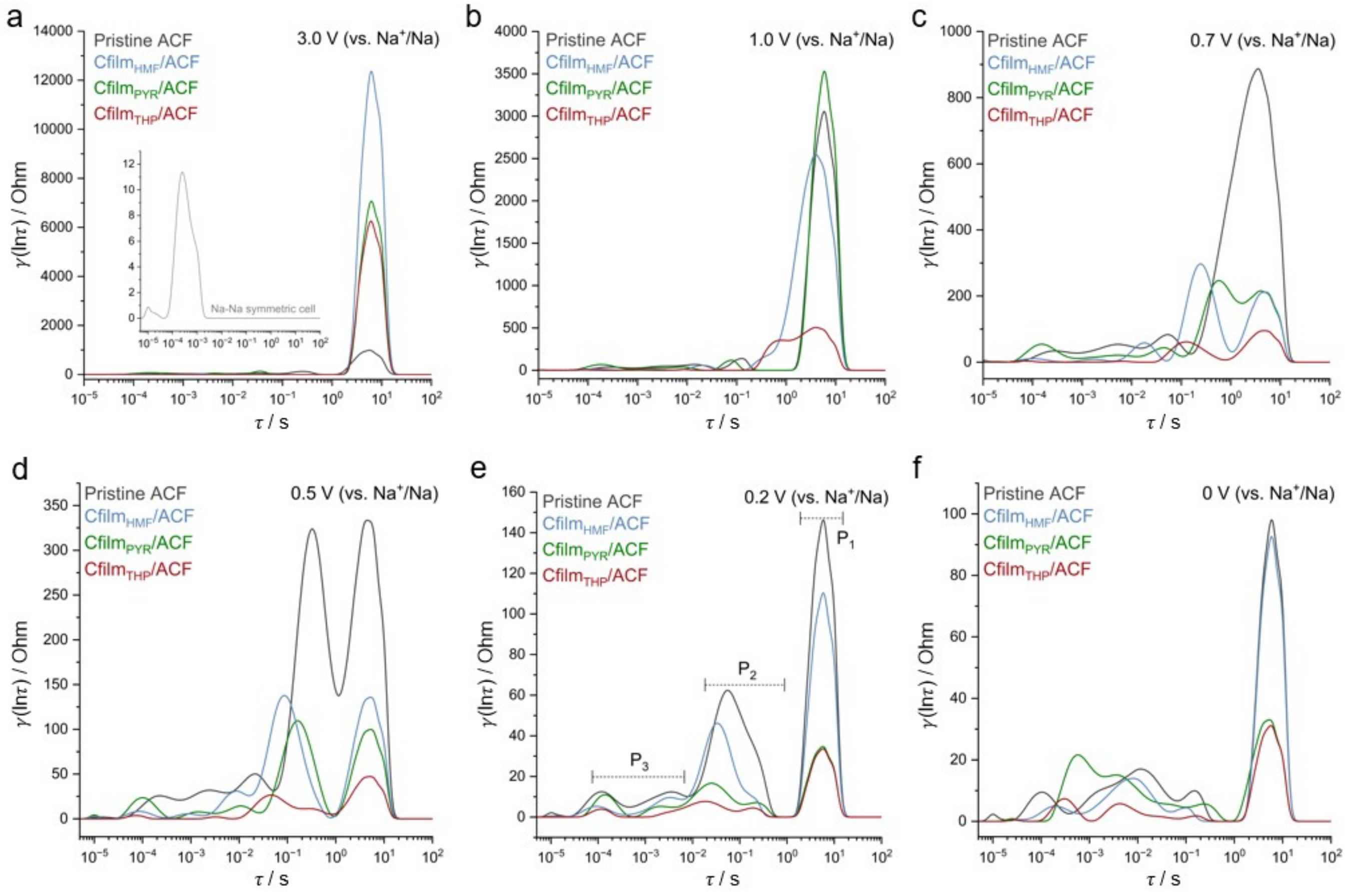

Fig. S18. DRT analysis of pristine ACF and Cfilm/ACF electrodes at different states of sodiation. **(a-f)** The impedance response is plotted as a function of relaxation time at various applied potentials from 3.0 V down to 0 V (vs. Na^+/Na). DRT spectra reveal distinct resistive contributions and relaxation processes as a function of potential. The inset in panel **(a)** shows the DRT response of a Na-Na symmetric cell.

Supplementary Note 14. GITT Analysis and Mechanistic Insights

The relative sodium-ion diffusion coefficients (D_{Na}) were calculated from galvanostatic intermittent titration technique (GITT) by applying current pulses (30 mA g^{-1}, 600 s), followed by a relaxation period of 3600 s, during which the potential was monitored until it approached a near-equilibrium value. This protocol minimizes contributions from ohmic resistance and kinetic overpotentials, ensuring that the voltage response reflects ion diffusion under near-equilibrium conditions. The voltage response during each pulse and subsequent relaxation was analyzed according to Fick's laws of diffusion. The effective diffusion coefficient was calculated using the first-order approximation of Weppner and Huggins, but with certain assumptions:[30, 31]

In its general form, the diffusion coefficient is given by:

$$D = \frac{4}{\pi}\left(\frac{iV_m}{z_A F S}\right)^2\left[\frac{dE}{d\delta}/\frac{dE}{d\sqrt{t}}\right]^2 \tag{6}$$

Where i is the applied current, V_m is the molar volume of the electrode, z_A is the charge number of sodium, F is the Faraday constant, and S is the electrode surface area. The terms $\frac{dE}{d\delta}$ and $\frac{dE}{d\sqrt{t}}$ represent the slope of the equilibrium potential vs. composition and the transient potential change during the pulse, respectively. For sufficiently small currents (e.g., C/10) and short time intervals (typically ≤10 min), the potential response during the pulse can be considered linear with respect to $\sqrt{t}$. Under these additional conditions, the expression can be simplified to the more practical form:[32, 33]

$$D = \frac{4}{\pi\tau}\left(\frac{m_B V_M}{M_B S}\right)^2\left(\frac{\Delta E_s}{\Delta E_\tau}\right)^2 \tag{7}$$

Where τ is the pulse duration, m_B and M_B correspond to the actual and molar mass of the active material, respectively, while V_M denotes the molar volume and S represents the electrode surface area. The parameters ΔE_s (the change in steady-state potential during a relaxation step) and ΔE_τ (the potential change during a constant-current pulse) are directly extracted from the GITT profiles of the electrodes.

It is worth noting that the geometric factor $\frac{m_B V_M}{M_B S}$ can be simplified to the average electrode thickness (L). This follows from the fact that $\frac{V_M}{M_B}$ corresponds to the specific volume of the active material, such that multiplication with the electrode mass and normalization by surface area yields a thickness term. Adopting this simplification is common in the analysis of planar electrodes, as it avoids the need for precise knowledge of molar volume and molar mass, while still capturing the physical dimension that governs diffusion length scales. Nevertheless, it is important to emphasize that the resulting diffusion coefficients should be regarded as effective values rather than absolute ones. The simplification assumes uniform electrode geometry, homogeneous composition, and ideal linear potential response, which do not perfectly describe real electrodes. As such, the absolute magnitude may deviate from the true microscopic diffusion coefficient. Therefore, the diffusion coefficients here are reported as relative diffusion coefficients, which, although not representing absolute values, enable reliable comparison across samples.

Fig. S19 compares the GITT behavior of pristine ACF and Cfilm/ACF electrodes derived from different precursors. The discharge-charge profiles (**Fig. S19a**) show distinct electrochemical responses upon Cfilm deposition. Pristine ACF shows smooth potential curves with relatively fast relaxation, while Cfilm-coated electrodes exhibit specific differences in polarization and relaxation dynamics. The schematic illustration (**Fig. S19b**) outlines the pulse-relaxation sequence, defining the key potential values used in diffusion coefficient calculations. The linear relationship between potential and the square root of time during each pulse (**Fig. S19c**) shows sufficient correlation (R^2 > 0.995 for all samples), confirming that the simplification of Eq. 6 to Eq. 7 is valid under our experimental conditions.

The resulting diffusion coefficients (**Fig. 4a, main**) highlight the impact of Cfilm deposition and precursor chemistry on sodium ion transport. During sodiation, $\text{Cfilm}_{\text{PYR}}$/ACF exhibits a small dip in D_{Na} around 0.7 V (vs. Na^+/Na, unless otherwise stated), which coincides with a minor plateau observed in its GCD curve, suggesting momentarily diffusion hindrance. Outside this feature, all Cfilm/ACF electrodes reveal diffusion coefficients in the range of 10^{-8}-10^{-9} cm^2 s^{-1}, while pristine ACF remains consistently lower. As

the potential approaches the plateau region, all Cfilm/ACF samples show a sharp decrease in D_{Na}, followed by a rapid recovery. This two-step response reflects the same mechanistic sequence previously described: the initial drop corresponds to electrostatic crowding as sodium begins to saturate pore entrances, while the subsequent rise is associated with the onset of quasi-metallic sodium clustering within confined micropores.[29, 34] These kinetic signatures are consistent with our earlier mechanistic study, where clustering related to the pore-filling was directly confirmed.[29]

During desodiation, the low-potential behavior mirrors sodiation. Starting near 0 V, the diffusion coefficient (D_{Na}) rises sharply as the quasi-metallic clusters formed in the plateau begin to deconstruct and transport pathways open, followed by a gradual decrease as the system exits the clustering regime and returns to surface-controlled transport. In the slope region (above about 0.2 V), $Cfilm_{PYR}$/ACF and $Cfilm_{THP}$/ACF maintain slightly higher D_{Na} than $Cfilm_{HMF}$/ACF, consistent with heteroatom-modified surfaces that could aid ion desolvation and transfer. Two subtle features are notable: (i) $Cfilm_{PYR}$/ACF shows a small dip near 0.7 V, mirroring the minor feature seen in sodiation and likely reflecting the same process; (ii) $Cfilm_{HMF}$/ACF exhibits an additional dip around 0.5 V that is absent in sodiation, pointing to a process slightly hinders sodium transport, for example the stronger binding or local trapping at oxygen functional groups are not neglected.

To further explore the effect of carbon film growth, a thickness-dependent study was conducted using HMF as the model precursor. HMF was deliberately selected because it yields oxygen-containing Cfilms without the additional complexity introduced by nitrogen or sulfur heteroatoms, which are known to strongly influence electronic properties. By excluding these heteroatom effects, the system remains chemically simpler, allowing the influence of film thickness on sodium-ion diffusion to be studied in isolation. The discharge-charge profiles (**Fig. S20a**) clearly show systematic differences in potential response with different Cfilm thickness. The corresponding linear fits of potential versus the square root of time (**Fig. S20b**) maintain excellent correlation coefficients ($R^2 > 0.995$ for all samples), validating once more the reliability of the GITT approximation across all thicknesses.

The calculated diffusion coefficients reveal a strong dependence on thickness. During sodiation (**Fig. S20c**), all Cfilm/ACF electrodes exhibit an initial sharp increase in D_{Na} at low potentials, followed by stabilization in the 10^{-10}-10^{-9} $cm^2\ s^{-1}$ range. Interestingly, the sharp decrease of Cfilm(thin)/ACF shows at low potentials, which is shifted slightly toward higher potentials compared to the other samples. This shift suggests that the saturation of available sites begins earlier in potential, which in practice may translate

into a reduced energy requirement for pore-filling. While this behavior is consistent with the thinner architecture offering less resistance to ion access, it also highlights a trade-off, as very thin coatings can lead to less uniform structure. During desodiation (**Fig. S20d**), the overall trends mirror sodiation, with diffusion slowing as clusters dissolve and recovering toward higher potentials. A clear thickness effect is again observed in the plateau region: the longer the plateau, the more pronounced the decrease in diffusion coefficient, reflecting the continuous process of pore-filling until the available pores are saturated.

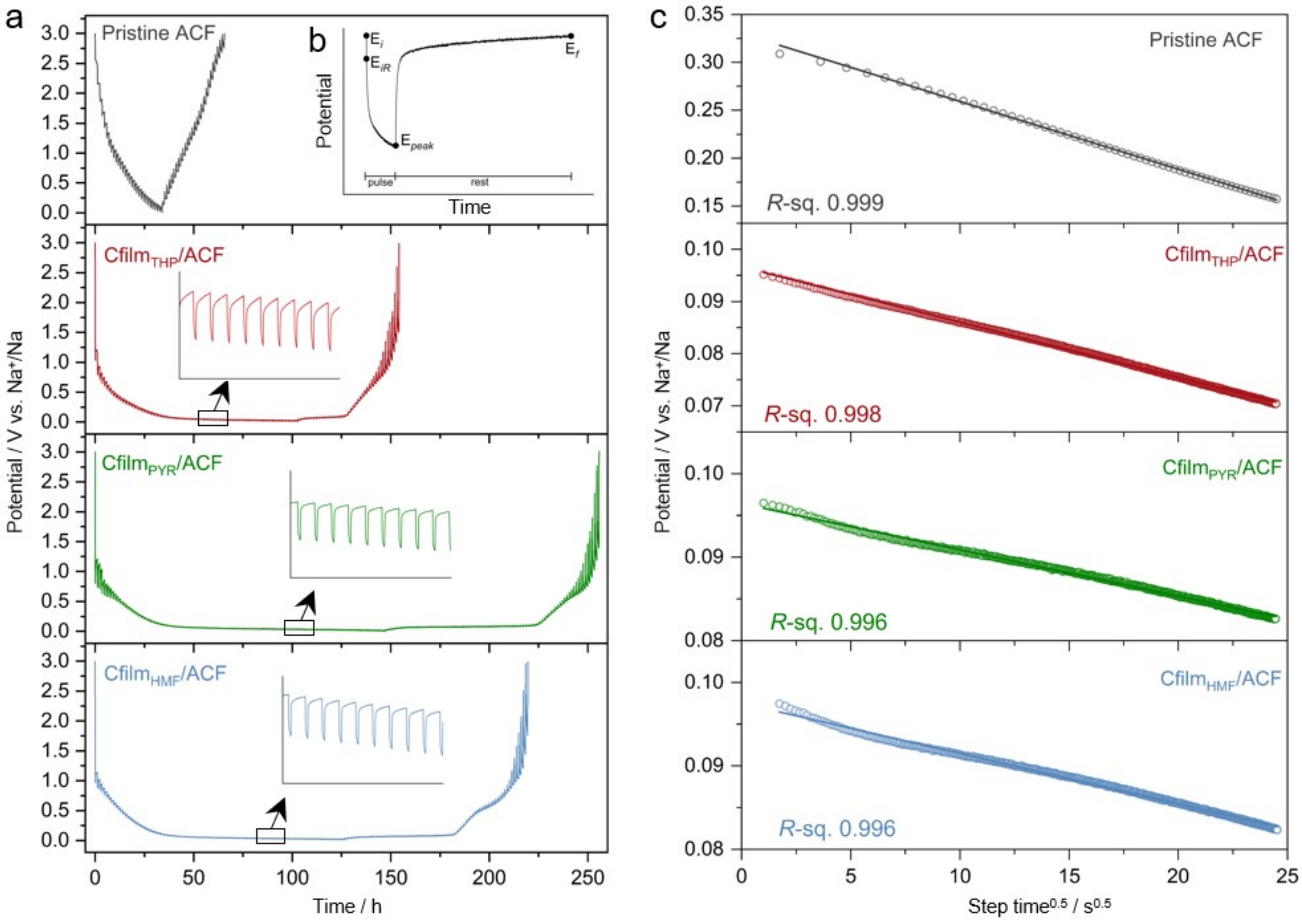

Fig. S19. GITT analysis of pristine ACF and Cfilm/ACF electrodes. **(a)** Full GITT discharge-charge profiles collected at a current of 30 mA g^{-1} using a pulse duration of 600 s followed by a relaxation period of 3600 s, showing distinct responses among the samples. **(b)** Schematic illustration of the GITT method, indicating key potential values. **(c)** Plots of potential vs. the square root of time during the pulse. Linear trends and high correlation coefficients are required for the simplification of the GITT model.

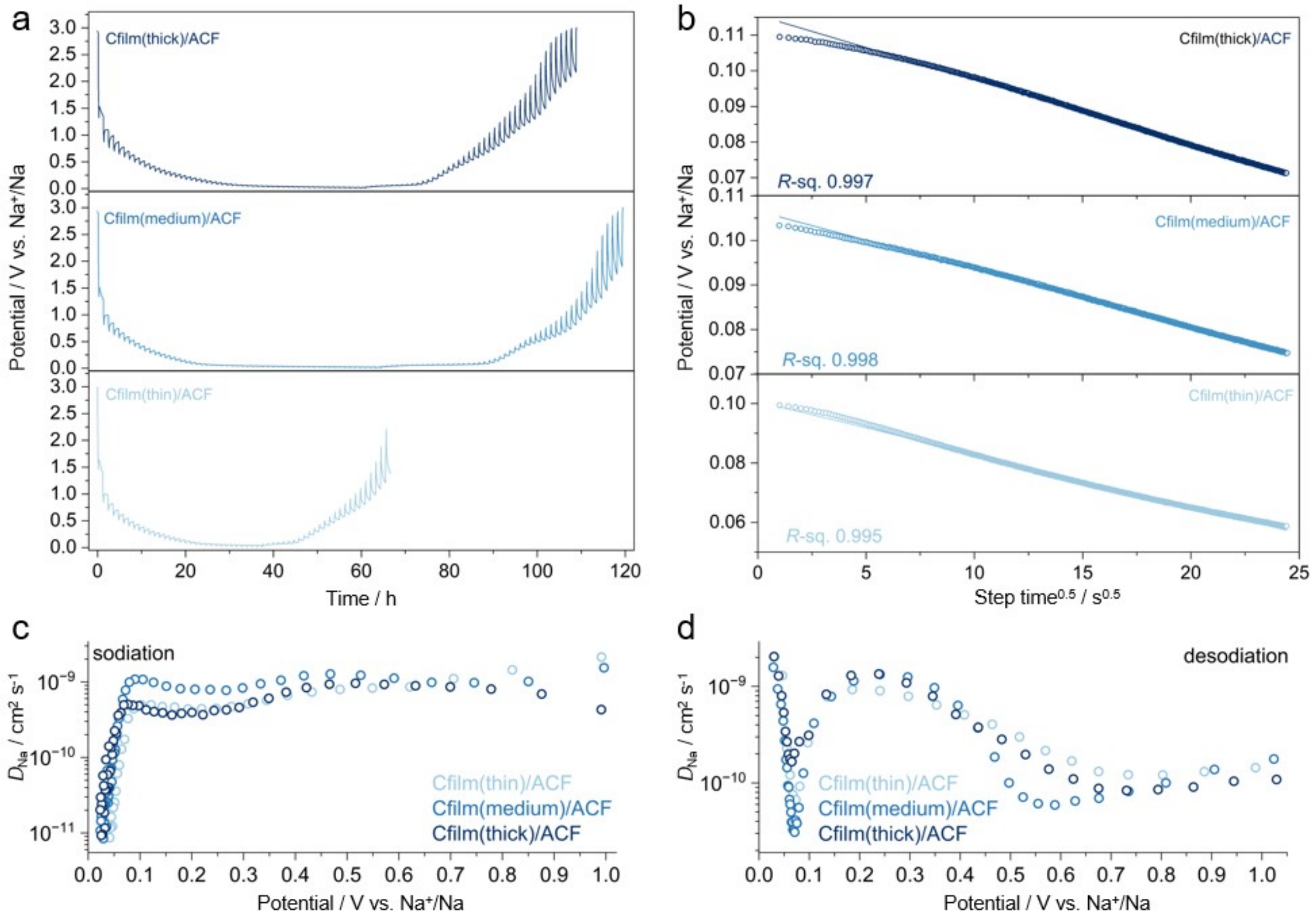

Fig. S20. GITT analysis of Cfilm/ACF electrodes with varying carbon film thicknesses. **(a)** Full GITT discharge-charge profiles collected at a current of 30 mA g^{-1} using a pulse duration of 600 s followed by a relaxation period of 3600 s. The profiles show systematic differences in potential response with different carbon film thickness. **(b)** Plots of potential vs. the square root of time during the pulse. Linear trends and high correlation coefficients are required for the simplification of the GITT model. **(c, d)** Calculated sodium-ion diffusion coefficients as a function of potential during **(c)** sodiation and **(d)** desodiation.

***Ex-situ* PDF analysis.** To further probe the sodium storage mechanism, *ex-situ* pair distribution function (PDF) analysis was performed using a synchrotron X-ray source for the $Cfilm_{PYR}$/ACF electrode. All samples were prepared inside a glovebox, sealed in Kapton bags, and transferred to the facility. **Fig. S21a** shows the scattering profile, I(*q*), together with the reduced structure function, F(*q*), obtained after background corrections. The transformation into F(*q*) enhances local structural correlations and enables the extraction of the real-space pair distribution function, G(*r*). The dry $Cfilm_{PYR}$/ACF electrode exhibits characteristic short-range structural correlations typical of porous carbons (**Fig. S21b**), in good agreement with previous reports on porous and hard carbon frameworks.[35, 36] The observed correlations mainly originate from local C–C atomic arrangements in the carbon materials' domains. To validate the *ex-situ* procedure, a control electrode exposed to electrolyte, washed (in DEC), and dried without electrochemical cycling was additionally measured (**Fig. S21**c). The PDF profile of the control electrode remains nearly identical to that of the dry electrode, indicating that residual electrolyte contributions are minimal and that the washing procedure effectively removes electrolyte-derived artifacts.

Upon sodiation to 0 V vs. Na^+/Na, significant changes in the PDF profile are observed, particularly through attenuation of C–C correlations (**Fig. S21c**). Rather than indicating structural degradation of the carbon, this behavior likely reflects modification and distortion of the local environments caused by the sodium incorporation. The ionic Na–C interactions alter the local scattering environment and reduce the coherence of the C–C correlations. After subsequent desodiation to 3 V, those features partially recover, indicating that the structural perturbation is largely reversible. Minor differences remaining after desodiation may originate from irreversible SEI formation during the initial cycle. Importantly, no pronounced Na–Na correlations characteristic of sodium metal-like environments are observed. This could potentially suggest either (i) sodium storage through highly confined clustering during the pore-filling process, or (ii) that the corresponding Na–Na contributions remain masked by the dominant carbon-related correlations, particularly since clusters are expected to produce broad and weak features that could only be pronounced with differential PDF analysis.[35]

Nevertheless, we emphasize that PDF analysis alone cannot unequivocally determine the electronic state of sodium, and therefore, the present measurements should be considered as complementary structural

evidence supporting the proposed pore-filling mechanism involving quasimetallic sodium species, as widely discussed in previous studies.[37, 38]

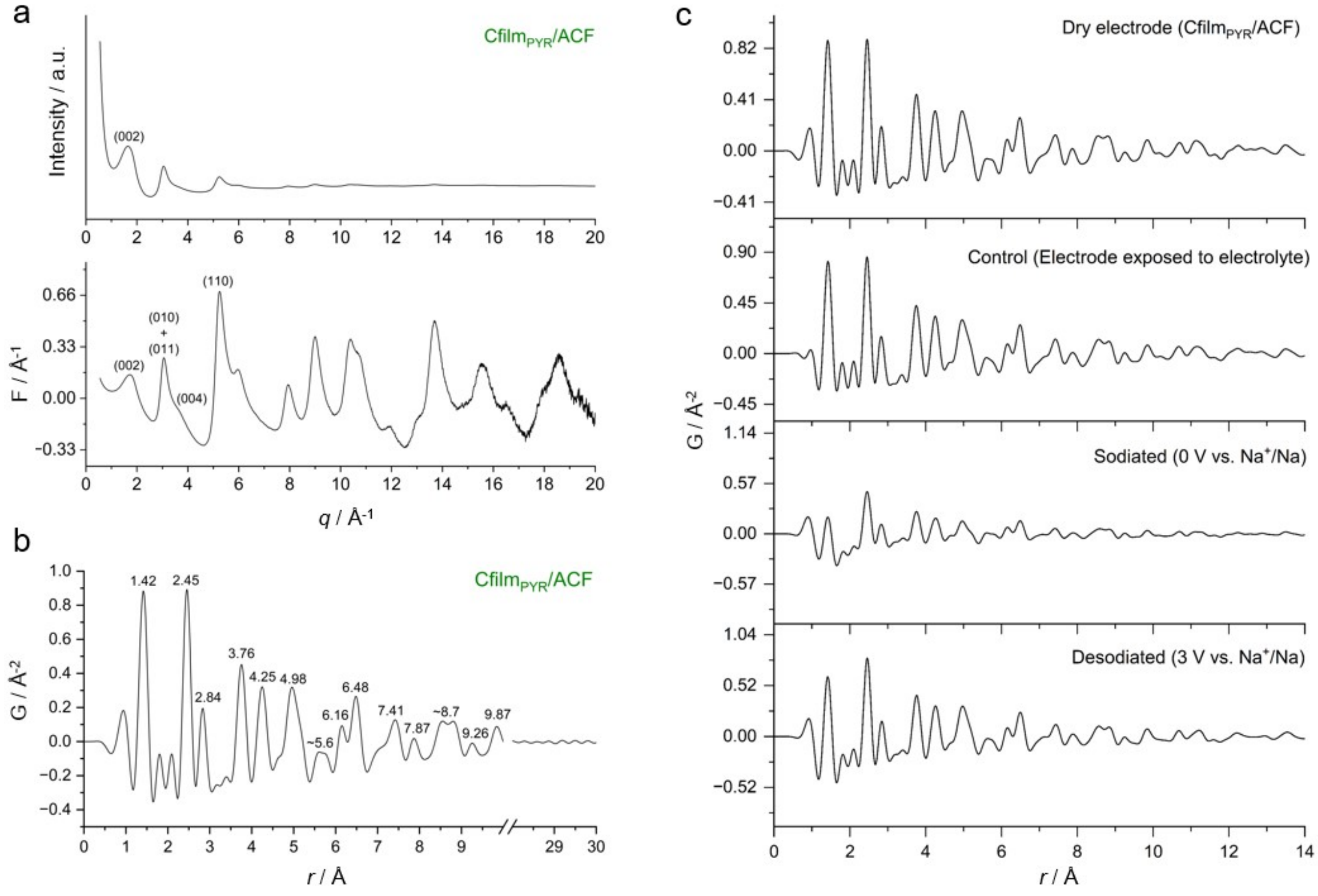

Fig. S21. PDF analysis of Cfilm$_{PYR}$/ACF. **(a)** Scattering intensity, I(q), and reduced structure function, F(q). **(b)** Pair distribution function, G(r), of the dry electrode. **(c)** PDF profiles of dry, electrolyte-exposed control, sodiated, and desodiated electrodes, showing reversible changes in local carbon correlations upon sodiation/desodiation without signatures of crystalline Na metal formation.

Supplementary Note 15. Effect of deposition temperature on structure and electrochemical performance

We also examined the effect of heat treatment on pristine ACF to verify that the electrochemical improvement observed in Cfilm/ACF samples arises solely from the presence of the carbon film rather than thermal modification of the substrate. As shown in **Fig. S22a**, both pristine ACF and pristine ACF annealed at 800 °C (without film deposition) exhibit nearly identical charge-discharge profiles, with low capacities and sloping voltage curves. This demonstrates that annealing alone does not enhance sodium storage capability, confirming that the improvement in electrochemical performance is directly linked to the deposition of the carbon film.

To systematically assess the influence of deposition temperature on the structural and electrochemical properties of the carbon film, a series of $Cfilm_{PYR}$/ACF electrodes were prepared at temperatures ranging from 600 to 1000 °C. As shown in **Fig. S22b**, the charge-discharge profiles reveal a clear dependence on deposition temperature. Among all samples, the $Cfilm_{PYR(800)}$/ACF delivers the highest reversible capacity and plateau capacity. In contrast, the slope capacity, associated with surface adsorption processes,[29] gradually decreases from 600 to 1000 °C, suggesting a gradual loss of defect sites and functional groups that serve as adsorption centers for sodium ions.

When the deposition temperature is increased to 900-1000 °C, the overall capacity decreases. This trend can be attributed to excess graphitization, which removes defect sites and heteroatom functionalities while tightening the carbon stacking, thereby limiting ion diffusion pathways within the carbon scaffold. At lower deposition temperatures, the carbonization process is milder, resulting in more disordered films that retain more nitrogen and oxygen functionalities. These features promote stronger sodium-carbon interactions and provide a more open, accessible structure for ion storage, thereby decreasing plateau capacity while increasing slope capacity. However, below 600 °C, film formation remains incomplete, and no significant deposition is observed, confirming that sufficient thermal energy is necessary to initiate carbon growth. These electrochemical observations are consistent with the Raman analysis, which corroborates the structural ordering with increasing deposition temperature. As shown in **Fig. S22c**, the Raman spectra reveal a clear structural evolution of the carbon films with increasing deposition temperature. The *G* band gradually shifts from approximately 1550 cm^{-1} to 1585 cm^{-1}, indicating the

transformation of the carbon network toward a more graphitic, sp^2-oriented structure.[3, 39] This shift reflects the development of larger conjugated domains and improved in-plane ordering, consistent with enhanced graphitization at elevated temperatures.

Interestingly, the *D* band also becomes more pronounced with increasing temperature, even as the films become more ordered. This trend can be understood in the context of the Ferrari-Robertson model of carbon evolution. As amorphous sp^2 carbon reorganizes into nanocrystalline graphitic domains, the *D* band intensifies because of the higher number of edge sites and domain boundaries.[3] In addition, vacancies, Stone-Wales defects, and turbostratic stacking formed during the reconstruction process contribute further to *D*-band activation.[40, 41] Consequently, the simultaneous upshift of the *G* band and growth of the *D* band signify not competing but coexisting structural effects.

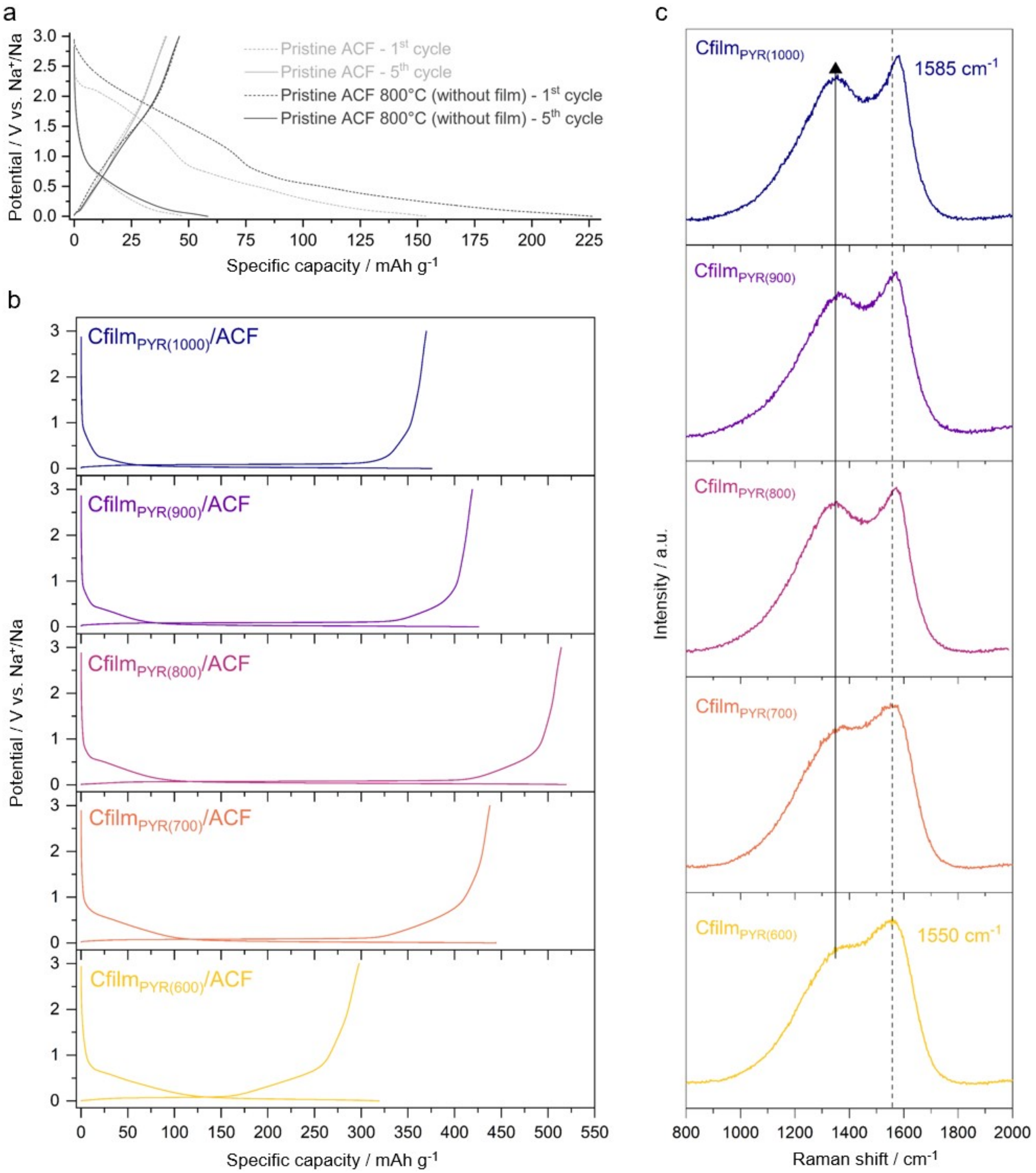

Fig. S22. Influence of deposition temperature on specific capacity and structural ordering of carbon films. **(a)** Comparison of GCD profiles for pristine ACF and pristine ACF treated at 800 °C without film deposition (no precursor for carbon film provided to the system) reveals that annealing alone does not improve the performance. **(b)** GCD profiles (5th cycle, 30 mA g^{-1}) of $Cfilm_{PYR}$/ACF materials at different deposition temperatures (600-1000 °C). **(c)** Corresponding Raman spectra of the carbon films, illustrating a gradual shift of the *G* band position from ~1550 cm^{-1} to ~1585 cm^{-1} with increasing deposition temperature, indicative of enhanced structural ordering in the carbon network.

Supplementary Note 16. Extension of the $Cfilm_{PYR}$ to different porous carbons

To evaluate the broader applicability of $Cfilm_{PYR}$, we extended the coating process to a series of widely used, commercially available carbon materials, including coconut-shell-based porous carbon (CSBC), wood-based porous carbon (WBC), CMK-3, activated carbon powder (ACP), carbide-derived carbon (CDC), Ketjenblack (KB), activated microwave-exfoliated graphite oxide (aMEGO), and hard carbon (HC). These substrates represent a diverse set of highly porous, high-surface-area carbon frameworks.

The $Cfilm_{PYR}$ coating was applied to these carbon materials following the same procedure described for the ACF substrate in the Experimental Section. **Fig. S23** illustrates how the $Cfilm_{PYR}$ coating restructures pore accessibility. The selected substrates represent distinct classes of porous carbons, ranging from highly open frameworks to closed-pore systems, enabling a comparative assessment of the coating effect. For porous carbons such as aMEGO and CDC, the coating strongly suppresses gas-accessible porosity. In contrast, hard carbon shows only minor changes in pore volume and distribution after coating, reflecting its largely closed pore structure that limits conformal infiltration. Together, these results demonstrate that the impact of the carbon film is most significant for open porous carbons.

This trend is further supported by the gas sorption analysis of ACF- and CSBC-based systems (**Fig. S24**). N_2 sorption reveals that the $Cfilm_{PYR}$ coating effectively suppresses mesoporosity, yielding negligible accessible surface areas (<10 m^2 g^{-1}) comparable to commercial hard carbon. In contrast, CO_2 sorption indicates the presence of residual ultramicroporosity (<1 nm), which remains partially accessible but significantly reduced relative to pristine materials. Notably, the extent of pore sealing depends on the initial substrate structure: while $Cfilm_{PYR}$/ACF still exhibits a CO_2-accessible surface area of ~83 m^2 g^{-1}, the more structurally confined CSBC-based system is reduced to ~15 m^2 g^{-1}. These results highlight that the Cfilm coating effectively modulates pore accessibility, but complete sealing is governed by the intrinsic pore architecture of the host carbon.

Sodium-ion half-cells were assembled using identical protocols. **Fig. S25** presents the initial GCD profiles at 30 mA g^{-1} for pristine and $Cfilm_{PYR}$-coated versions of each substrate. For all materials except hard carbon (HC), the ICE increases significantly after $Cfilm_{PYR}$ deposition, rising from ~20-25% in the pristine state to 41-71% after coating, depending on the substrate. This improvement originates from a substantial reduction in electrolyte-accessible surface area induced by the conformal carbon film. Pristine high-surface-area carbons expose extensive porosity and defect-rich sites to the electrolyte, which promotes excessive electrolyte decomposition and uncontrolled SEI formation. In contrast, the $Cfilm_{PYR}$ coating

mostly seals the porous scaffold and passivates reactive surface sites, thereby suppressing side reactions, consistent with the behavior observed for the ACF substrate. It is important to note, however, that the ICE is not governed solely by the gas-accessible surface area: while the suppression of N_2-accessible surface area correlates with improved stability, the CO_2-accessible ultramicroporosity does not directly determine first-cycle irreversibility. For example, samples with lower CO_2-accessible surface areas can still exhibit notable initial capacity loss, indicating that additional factors such as surface chemistry, pore accessibility at the electrolyte interface, and ion desolvation in confined regions play a critical role, as discussed in the main manuscript.

This trend does not extend to HC (**Fig. S25h**). Owing to its intrinsically closed-pore architecture and limited open porosity (**Fig. S23e**), HC already provides a confined environment for sodium storage and is therefore less amenable to further structural modification by conformal Cfilm. In this case, the deposition of an additional nitrogen-doped carbon layer primarily introduces new surface sites rather than beneficial pore restructuring, slightly increasing surface reactivity and resulting in a modest decrease in ICE. In **Fig. S23f**, pores around ~1 nm are largely suppressed after coating; however, the formation of new surface features leads to the emergence of pores around ~2 nm. These newly accessible mesopores facilitate electrolyte penetration, which contributes to the slight decrease in ICE.

Beyond the first cycle, the $Cfilm_{PYR}$ coating consistently enhances reversible capacity and induces a clear transition from sloping to plateau-type profiles across most porous carbon substrates. With appropriate precursor selection, total capacities exceeding 450 mAh g^{-1} can be achieved, confirming that the performance gains are not unique to ACFs but extend broadly to open porous carbon frameworks (**Fig. S26**). In the case of HC, although a slight decrease in ICE is observed due to its inherently closed-pore structure, a modest increase in reversible capacity is still achieved (**Fig. S26h**). This behavior highlights the expected trade-off and highlights that the Cfilm strategy is not effective for such carbons.

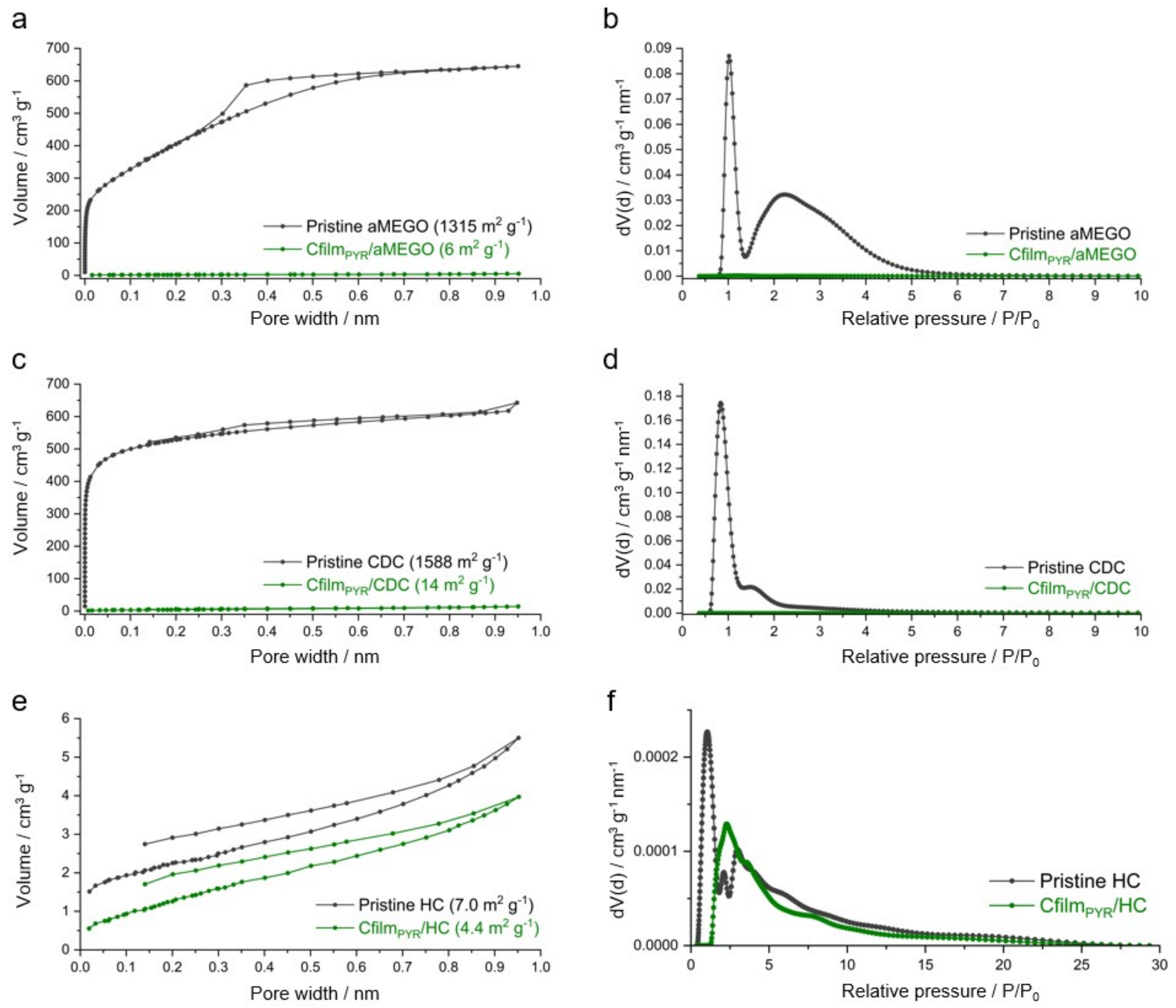

Fig. S23. Cumulative surface area (**a**, **c**, and **e**) and pore size distributions (**b**, **d**, and **f**) for pristine and Cfilm$_{PYR}$-coated aMEGO, CDC, and HC obtained from Ar-sorption analysis. The coating strongly suppresses gas-accessible porosity in highly open porous carbons (aMEGO, CDC), while only minor changes are observed for HC due to its intrinsically closed pore structure.

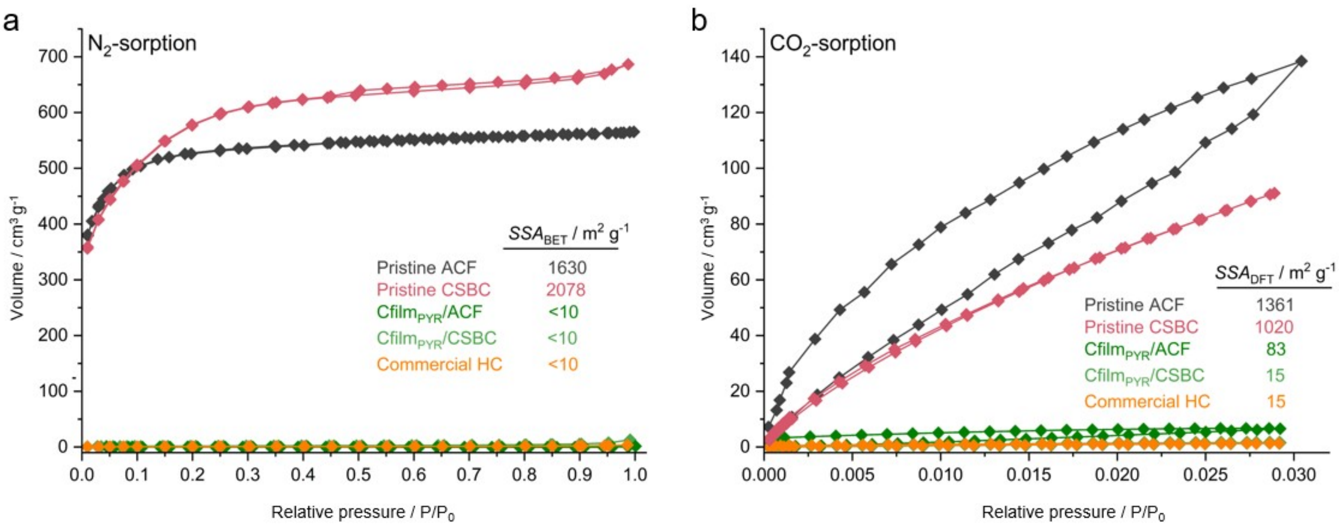

Fig. S24. N_2 **(a)** and CO_2 **(b)** sorption isotherms of pristine and $Cfilm_{PYR}$-coated carbons, showing suppressed mesoporosity and reduced ultramicroporosity after Cfilm deposition, with values comparable to commercial hard carbon.

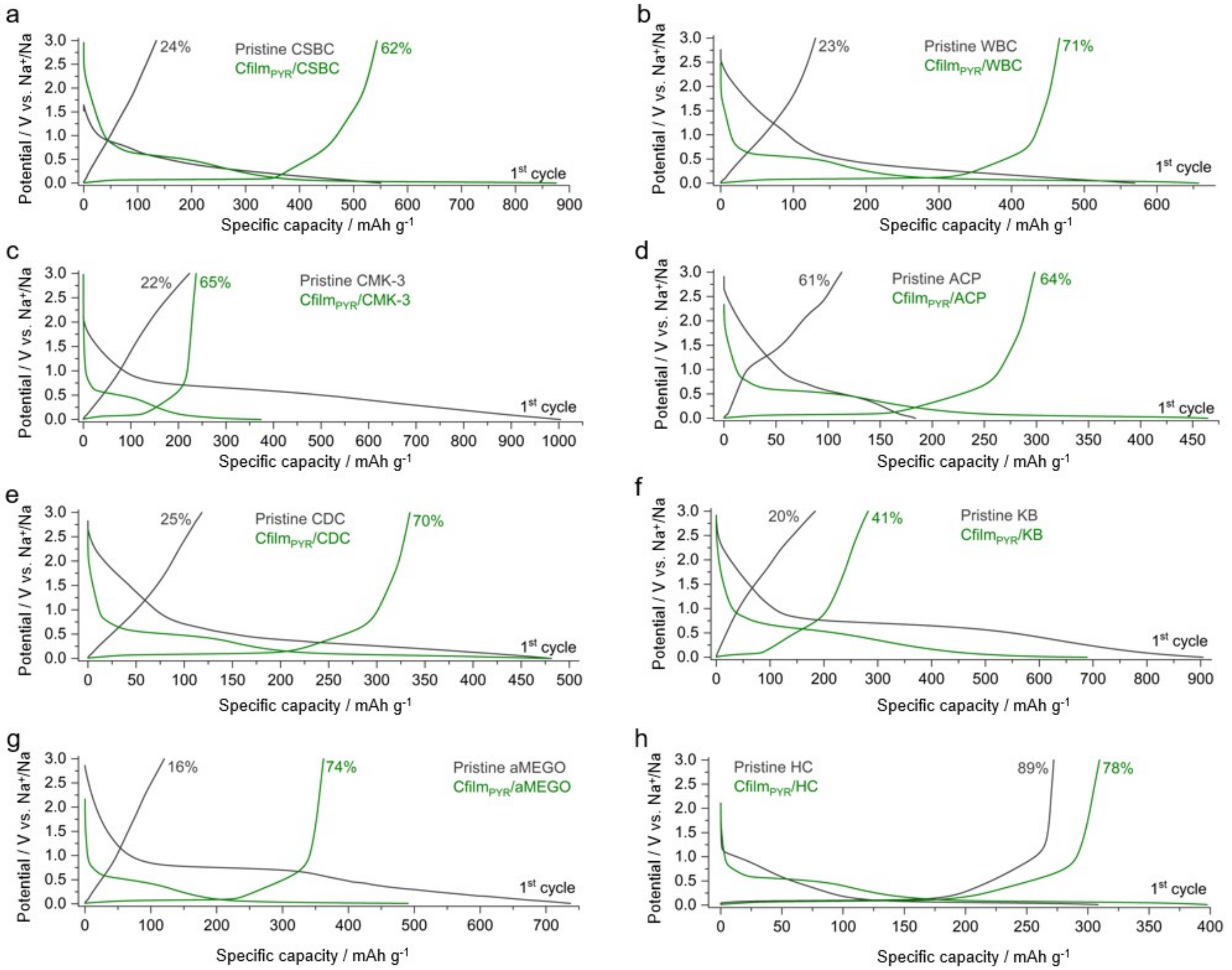

Fig. S25. Initial GCD profiles at 30 mA g^{-1} for pristine (black) and $Cfilm_{PYR}$-coated (green) porous carbons showing ICEs. Substrates include **(a)** CSBC, **(b)** WBC, **(c)** CMK-3, **(d)** ACP, **(e)** CDC, **(f)** KB, **(g)** aMEGO, and **(h)** HC.

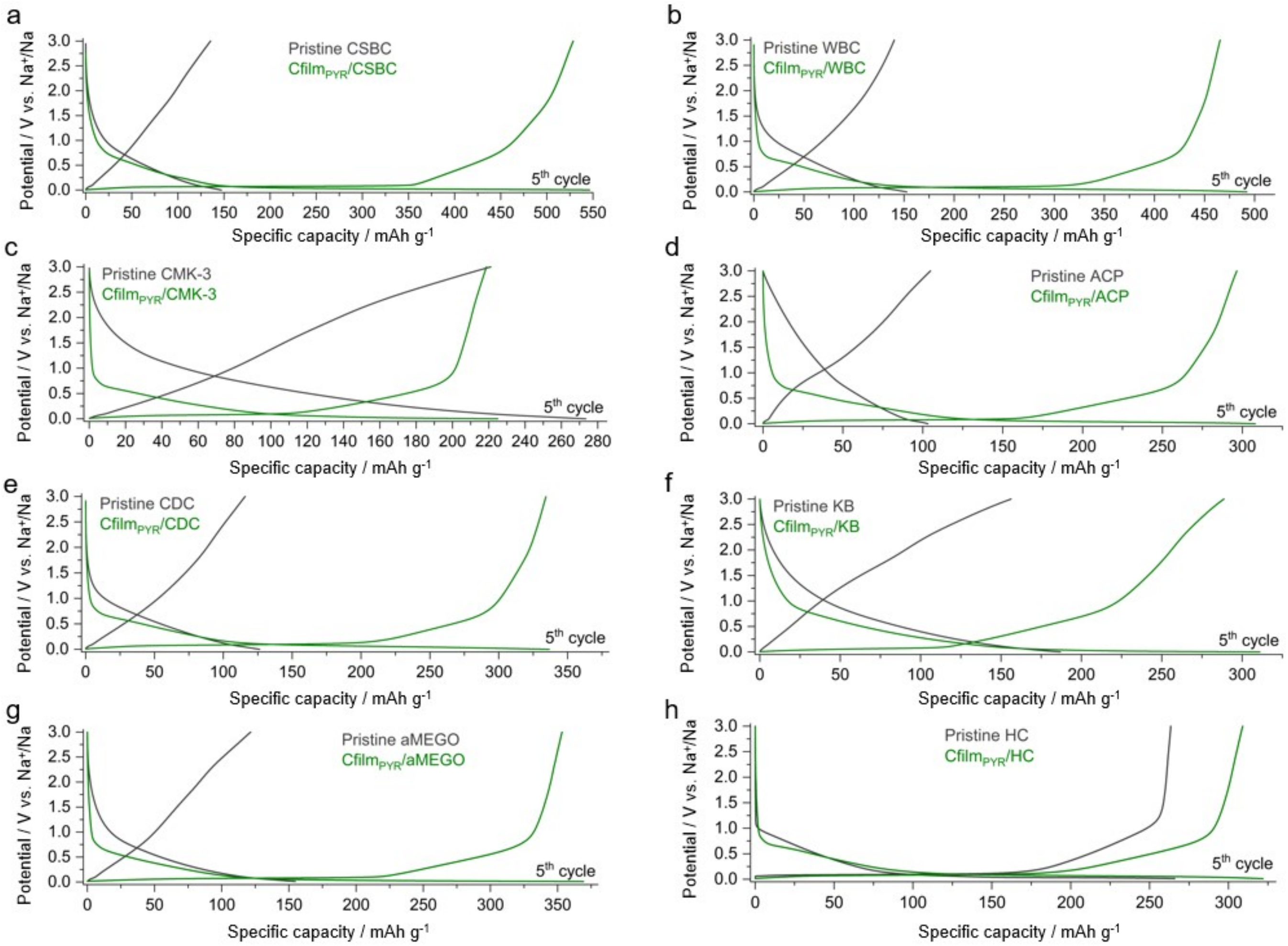

Fig. S26. Fifth GCD profiles at 30 mA g^{-1} for pristine (black) and Cfilm$_{PYR}$-coated (green) porous carbons showing stable capacities at this current density. Substrates include **(a)** CSBC, **(b)** WBC, **(c)** CMK-3, **(d)** ACP, **(e)** CDC, **(f)** KB, **(g)** aMEGO, and **(h)** HC.

Supplementary Note 17. Rate capability and cycling stability assessment of Cfilm-coated electrodes

Fig. S27a-d compares the rate-dependent electrochemical performance of pristine ACF and Cfilm/ACF electrodes. At low current densities (≤60 mA g^{-1}), all Cfilm/ACF electrodes exhibit high reversible capacities with stable voltage profiles, indicating efficient sodium storage and good ionic accessibility within the porous carbon structure. Among them, $Cfilm_{PYR}$/ACF consistently delivers the highest capacity, followed by $Cfilm_{HMF}$/ACF and $Cfilm_{THP}$/ACF.

As the current density increases, rate performance decreases across all samples, particularly above 300 mA g^{-1}, where sodium diffusion through the carbon layer and into confined pores becomes increasingly rate-limiting. Since the dominant storage mechanism in Cfilm/ACF electrodes involves diffusion-controlled pore-filling, higher rates shorten the diffusion time and restrict ion transport, thereby reducing the capacity. Importantly, this rate behavior also depends strongly on the structural characteristics of the underlying substrate, particularly its porous architecture and ion-accessible surface area. The observed trend is therefore not primarily governed by the Cfilm coating method itself but rather by the intrinsic properties of the substrate.

The Coulombic efficiency (CE) (**Fig. S27f**) provides further insight into the interfacial stability of the electrodes. Pristine ACF exhibits a notably lower CE, particularly during the early cycles, which can be attributed to its oxygen-rich and unstable surface functionalities that promote parasitic reactions. In contrast, all Cfilm-coated electrodes maintain high and stable Coulombic efficiencies above 99% after the initial cycles, indicating that the carbon films effectively suppress side reactions and promote highly reversible sodiation and desodiation processes.

Because the $Cfilm_{PYR}$/ACF electrode shows the highest reversible capacity among all samples but limited rate capability at high current densities, its long-term stability was evaluated at 30 mA g^{-1} to minimize polarization effects and ensure consistent sodiation-desodiation behavior. As shown in **Fig. S27g**, the electrode maintains a stable capacity over nearly three months of continuous cycling, demonstrating the durability of the Cfilm coating and a stable electrode-electrolyte interface. During the first 50 cycles, the capacity retention remains close to 95%. Toward the end of testing, however, a gradual capacity fading is observed, with retention decreasing to around 80% after 100 cycles. This degradation is attributed not only to the $Cfilm_{PYR}$/ACF intrinsic properties but is also strongly influenced by the instability of the sodium counter electrode during prolonged cycling. To further investigate this behavior, we point to the E_{ce} (vs. Na^+/Na) vs. capacity plot (**Fig. S27h**). As cycling proceeds, the Na plating/stripping potential

shifts. This behavior strongly suggests an increasing polarization of the Na metal electrode, associated with dead-sodium formation, gradual loss of active Na surface area, or contamination. Such instability may cause uneven sodium redistribution and local sodium depletion, while the accumulation of inactive sodium residues can further increase cell resistance and reduce the apparent capacity. These phenomena are well known in long-term sodium half-cell testing, particularly under low current densities and extended cycling times, and highlight the importance of stable counter-electrode configurations.[42]

The rate capability of carbon electrodes is influenced by both material structure and electrode-level parameters, which together control ion transport and storage kinetics. To systematically evaluate these effects, we designed a series of controlled experiments. In **Fig. S28a**, the effect of CVD deposition temperature (600-1000 °C) on the rate performance of Cfilm/ACF is presented. Increasing deposition temperature leads to a higher degree of structural ordering, which, while beneficial for electronic conductivity, results in a denser carbon framework with reduced pore accessibility. This densification limits ion transport pathways and increases diffusion resistance, particularly at higher current densities where transport becomes rate-limiting. As a result, samples prepared at higher temperatures exhibit reduced rate capability. For example, the $Cfilm_{PYR(600)}$/ACF sample shows superior rate performance, which can be attributed to its lower degree of graphitization and higher contribution from the sloping capacity region. Since the sloping region is associated with faster surface-driven processes, this leads to improved capacity retention at high rates compared to higher deposition temperatures.

Fig. S28b highlights the influence of electrode mass loading on the rate capability of $Cfilm_{PYR(800)}$/ACF. Increasing the areal loading results in thicker electrodes, which increases ionic diffusion distances and tortuosity within the electrode. This leads to enhanced transport limitations and polarization at higher current densities, thereby reducing the accessible capacity. These results emphasize the importance of electrode design parameters, such as mass loading and associated calendaring conditions, in determining rate performance, as both directly affect ion transport and electrode density. Although the rate performance at a lower mass loading (~0.5 mg cm^{-2}) is significantly better, the data presented in the main manuscript correspond to electrodes with a mass loading of ~1.5 mg cm^{-2} to ensure better comparability with the literature, where typical values generally lie in the range of 1-1.5 mg cm^{-2}.

In **Fig. S28c**, the effect of different carbon substrates on the electrochemical behavior of $Cfilm_{PYR(800)}$-coated electrodes is shown. Despite identical coating conditions, the underlying substrate strongly influences the resulting performance due to differences in carbon structure. Substrates that promote a

larger contribution from the sloping capacity region exhibit better rate capability. In contrast, substrates that favor more pronounced plateau behavior tend to show reduced rate performance, reflecting the slower kinetics associated with ion transport and storage in nanoconfined pores. These results demonstrate that both the carbon film and the underlying substrate play a critical role in governing the balance between capacity and rate capability.

Reproducibility tests using three randomly selected cells show highly consistent GCD profiles and reversible capacities (**Fig. S29**). The averaged reversible capacities (mean ± s.d., n = 3) are 519 ± 14 mAh g^{-1} for $Cfilm_{PYR}$/ACF, 381 ± 10 mAh g^{-1} for $Cfilm_{HMF}$/ACF, and 255 ± 9 mAh g^{-1} for $Cfilm_{THP}$/ACF. Comparable results reproduced across different working groups further validate the reliability and transferability of the approach.

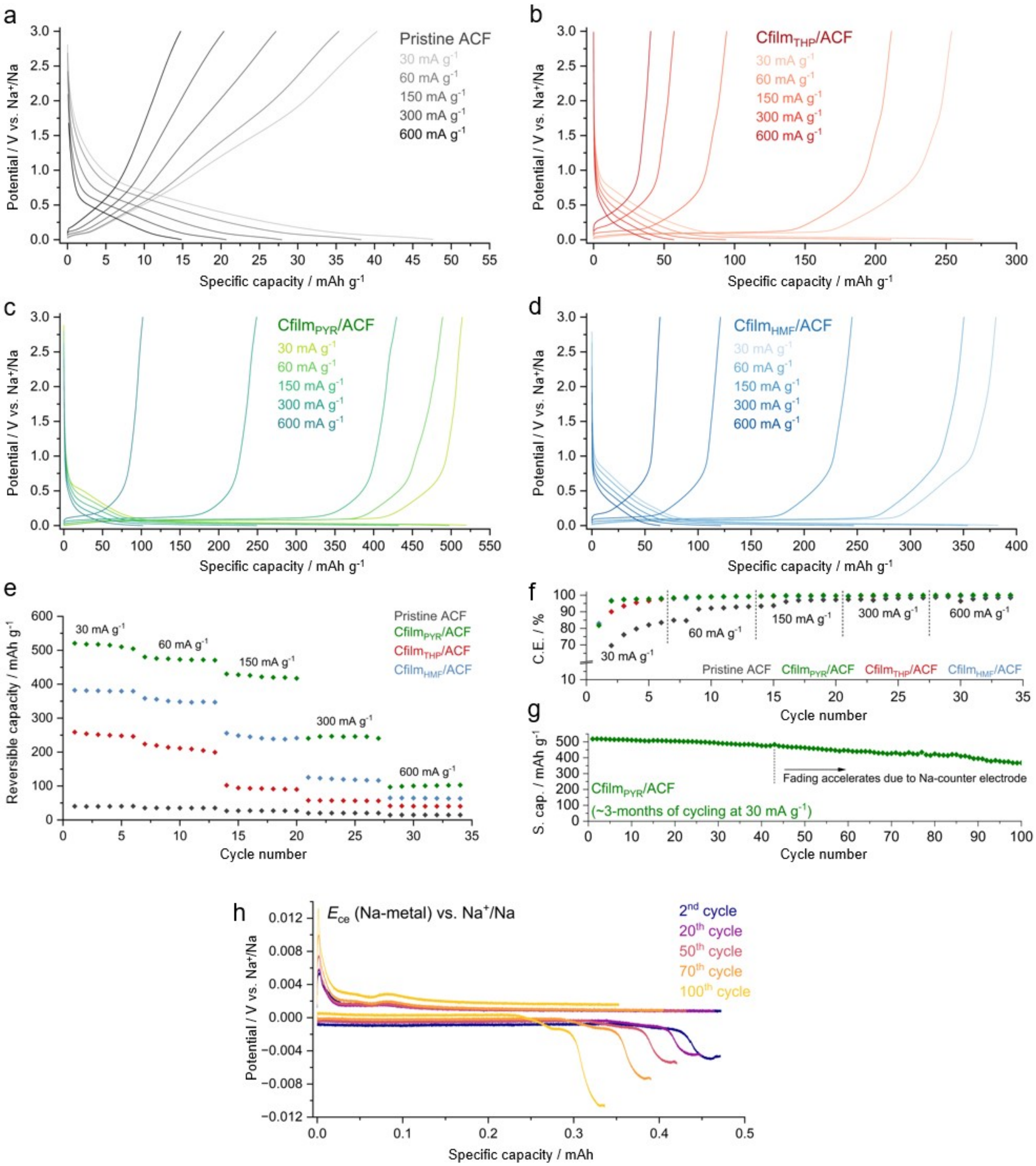

Fig. S27. Rate performance of pristine ACF and Cfilm/ACF electrodes and cyclic stability of Cfilm_{PYR}/ACF. **(a-d)** GCD profiles of **(a)** pristine ACF, **(b)** Cfilm_{THP}/ACF, **(c)** Cfilm_{PYR}/ACF, and **(d)** Cfilm_{HMF}/ACF at various current densities from 30 to 600 mA g^{-1}. **(e)** Reversible capacity versus cycle number at each current density. **(f)** Coulombic efficiencies of all samples at different current densities. **(g)** Long-term cycling of Cfilm_{PYR}/ACF at 30 mA g^{-1}. **(h)** Electrochemical potential profile Na-metal versus for the 2nd, 20th, 50th, 70th, and 100th cycles. The evolution of the polarization behavior with cycle number indicates instability upon prolonged cycling.

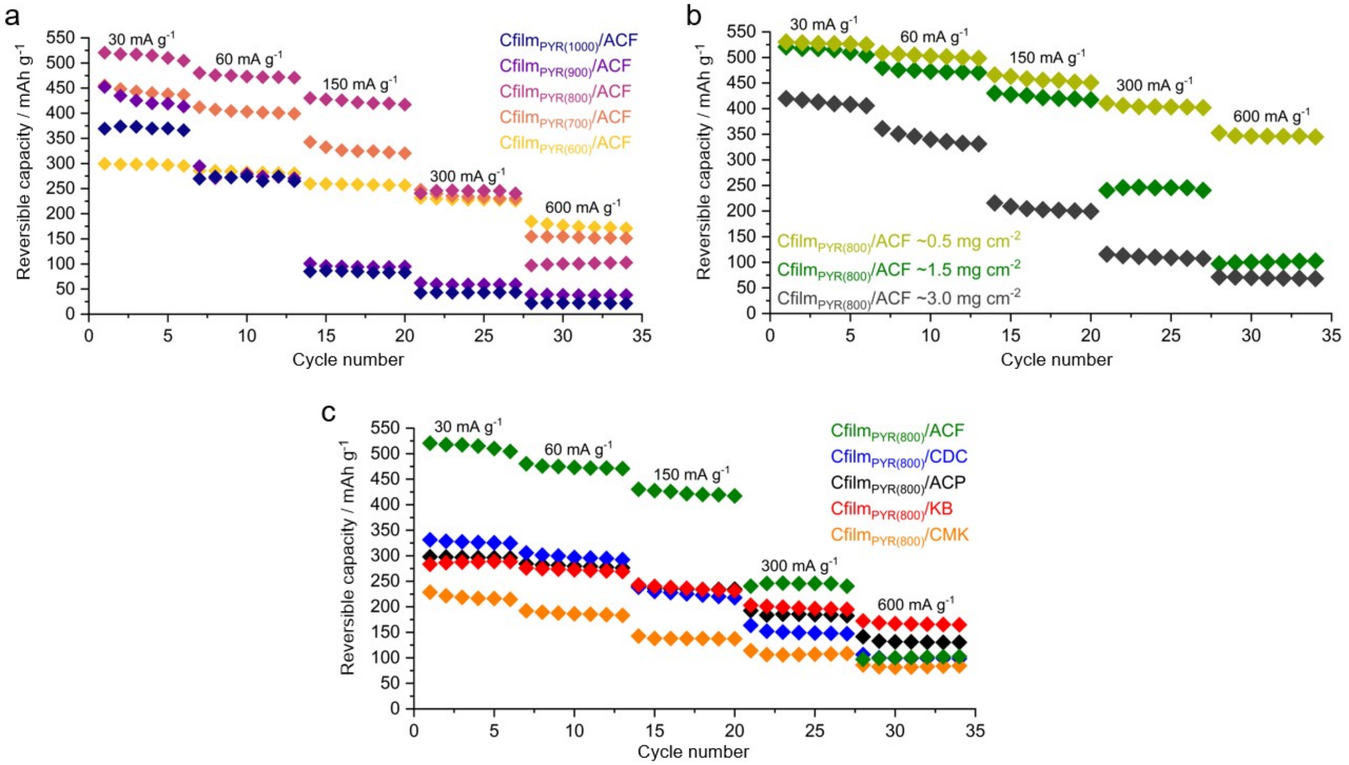

Fig. S28. Rate capability of $Cfilm_{PYR}$-coated electrodes. **(a)** Reversible capacity versus cycle number at different current densities for $Cfilm_{PYR}$ samples prepared at different deposition temperatures. **(b)** Effect of areal mass loading (~0.5-3.0 mg cm^{-2}) on the rate performance of $Cfilm_{PYR(800)}$/ACF electrodes. **(c)** Influence of different carbon substrates (ACF, CDC, ACP, KB, and CMK) on the rate capability.

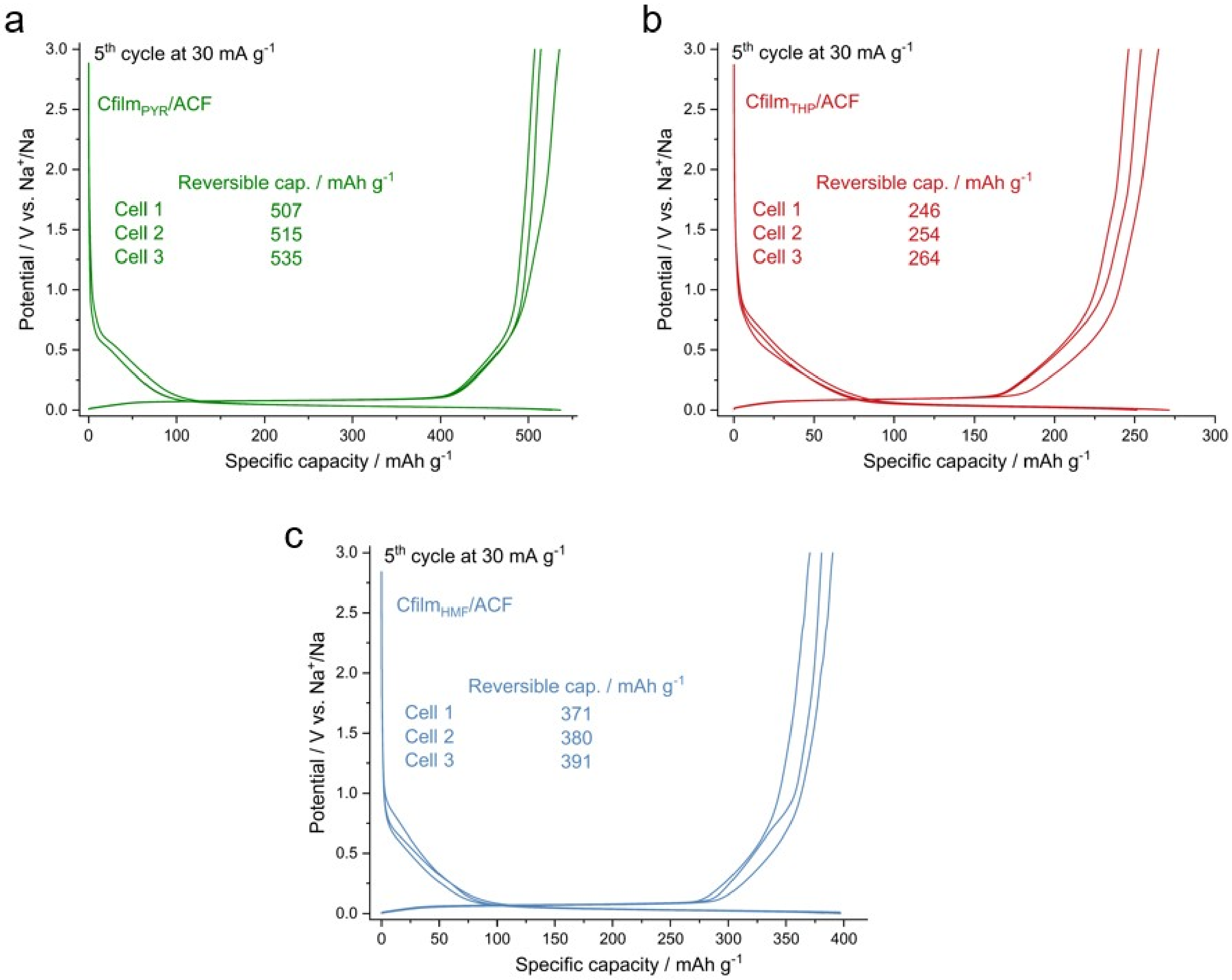

Fig. S29. GCD profiles of three independently assembled half-cells measured at 30 mA g^{-1} (5th cycle) for **(a)** $Cfilm_{PYR}$/ACF, **(b)** $Cfilm_{THP}$/ACF, and **(c)** $Cfilm_{HMF}$/ACF.

Supplementary Note 18. Benchmarking plateau capacities of $Cfilm_{PYR}$-coated carbons

To contextualize the sodium-storage performance of the $Cfilm_{PYR}$ hybrids, we benchmarked the reversible low-voltage plateau capacities against representative state-of-the-art hard carbons reported in the literature. The plateau region at low potential is widely regarded as the most relevant contribution for practical SIB anodes because it directly supports high energy density when paired with cathodes. For a consistent comparison across studies, we therefore focus on the plateau capacity during desodiation below 0.15 V (vs. Na^+/Na). Plateau values were taken from the corresponding reports where available; when needed, reference GCD curves were digitized from published figures (RGB-based image analysis) to enable direct visualization (**Fig. S30**).

As summarized in **Table S3**, $Cfilm_{PYR}$/ACF delivers a reversible plateau capacity of 420 mAh $g^{-1,}$ exceeding the closest benchmark examples. Notably, the gain is not limited to the plateau alone: the same electrode reaches a reversible capacity >500 mAh g^{-1}, demonstrating that the conformal film restructures porous carbons into a storage regime typically associated with optimized hard carbons, while preserving high overall capacity.

Beyond ACF, the same film concept translates to other porous carbon substrates. For example, $Cfilm_{PYR}$/CSBC achieves a plateau of 365 mAh g^{-1}, comparable to high-performing benchmark hard carbons around 350-360 mAh g^{-1} (**Table S3**). Likewise, $Cfilm_{PYR}$/WBC reaches 320 mAh g^{-1}, still surpassing the plateau capacity of widely cited hard carbons. These trends support that the performance increase is not an isolated outcome of one precursor, but a consequence of the conformal, reaction-limited CVD film growth that tightens pore networks, suppresses parasitic surface reactivity, and creates nanoconfined domains favorable for plateau-type sodium storage.

Table S3. Benchmarking of reversible plateau capacities (<0.15 V vs. Na^+/Na) and initial Coulombic efficiencies of the $Cfilm_{PYR}$/ACF against benchmark carbonaceous anodes from the literature.

Material	Plateau capacity at desodiation <0.15V (vs. Na^+/Na) / mAh g^{-1}	ICE / %	Reference
**$Cfilm_{PYR}$/ACF**	ca. 420	81	This work
**HC600-1500**	ca. 400	88	[43]
**RC-90-2M-NDD**	ca. 400	82	[44]
**SC-4**	ca. 385	80	[45]
**EPNHC-1800**	ca. 380	84	[46]
**POP_{70}-600-1600**	ca. 360	89	[47]
**ZGHC-1500**	ca. 350	72	[48]
**HCMP-CO_2**	ca. 340	90	[49]
**HC**	ca. 340	80	[50]
**Starch-HC**	ca. 320	87	[51]
**SPC-G-0.010**	ca. 300	86	[52]
**FC-3h-1300**	ca. 300	80	[53]
**HC-21-1550**	ca. 300	86	[54]
**HC-1500**	ca. 300	85	[55]

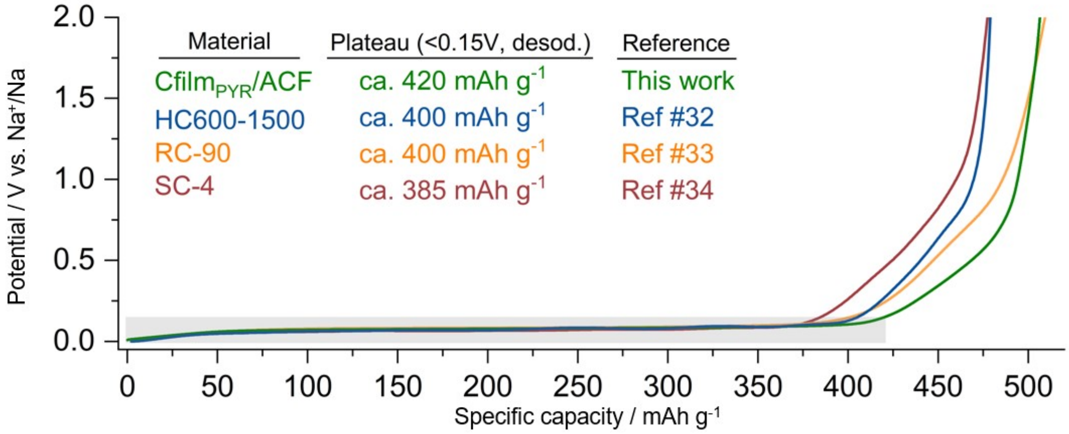

Fig. S30. GCD curves of Cfilm$_{PYR}$/ACF benchmarked against state-of-the-art samples, showing superior plateau capacity. Reference curves were digitized from the literature using RGB image analysis.

Supplementary Note 19. Full-cell evaluation of $Cfilm_{PYR}$/ACF

To demonstrate practical relevance, a proof-of-concept full-cell was assembled using $Cfilm_{PYR}$/ACF as the anode and $Na_3V_2(PO_4)_2F_3$ (NVPF) as the cathode (**Fig. S31**). NVPF was selected as a well-established, structurally robust cathode that delivers stable capacity and clearly defined voltage plateaus, allowing the anode behavior to be evaluated without cathode-limited artifacts. The full cell was assembled with a 1.02 N/P ratio. The $Cfilm_{PYR}$/ACF anode mass was 3.28 mg, while the NVPF cathode mass was 10.7 mg.

The GCD profiles of the $Cfilm_{PYR}$/ACF//NVPF full cell recorded at a current density of 30 mA g^{-1}. The cell exhibits a stable operating voltage window with clearly defined plateaus, indicating effective coupling between the low-voltage sodium storage process enabled by the $Cfilm_{PYR}$/ACF anode and the characteristic redox reactions of the NVPF cathode. Importantly, the presence of a pronounced discharge plateau confirms that the anode's low-voltage storage mechanism is retained under full-cell operation.

As shown in **Fig. S31b**, the full cell reveals a gradual capacity decay over repeated cycling. This behavior is primarily attributed to the non-optimized configuration, such as electrolyte formulation, and the absence of pre-oxidation strategies. Despite this, the cell maintains a high and stable coulombic efficiency.

Based on the active material masses of both electrodes, the assembled full cell delivers an energy density of approximately 240 Wh kg^{-1} (Eq. 8). This value highlights the advantage of the extended low-voltage plateau provided by the $Cfilm_{PYR}$/ACF anode, which enables efficient utilization of the cathode voltage window and directly contributes to enhanced full-cell energy density.

$$E = \int_0^Q \frac{U}{m} dQ \tag{8}$$

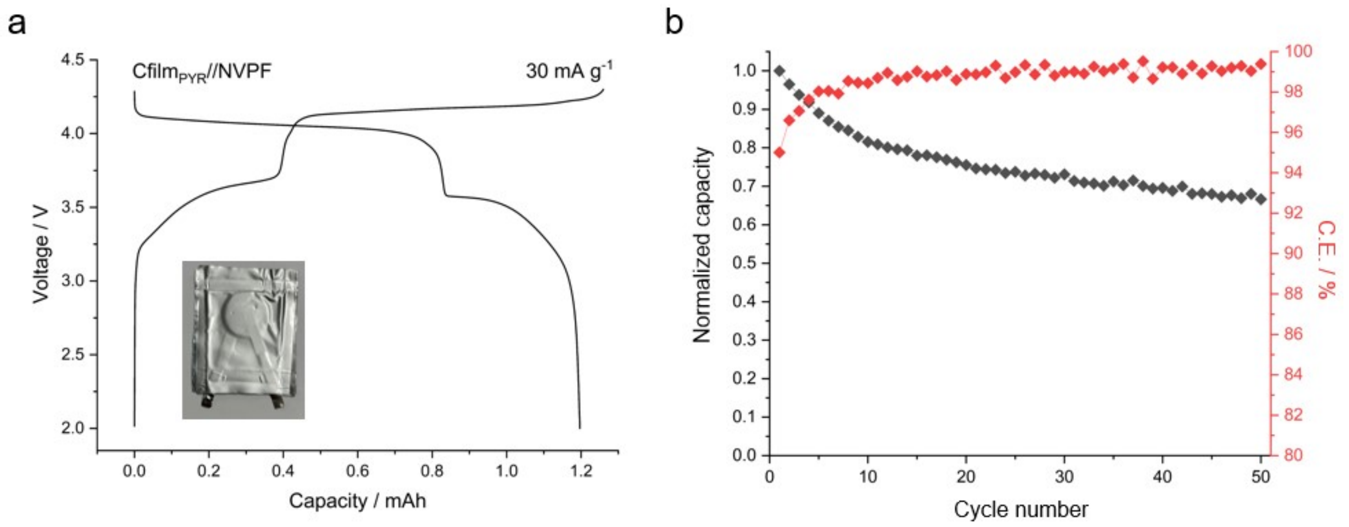

Fig. S31. (a) GCD profile of the Cfilm$_{PYR}$/ACF//NVPF full cell recorded at a current density of 30 mA g^{-1}. **(b)** Cycling performance of the full cell at 30 mA g^{-1}.

References

1. Eren EO, Senokos E, Song Z, Mondal B, Perju A, Horner T, *et al.* Hard carbon from a sugar derivative for next-generation sodium-ion batteries. *Materials Horizons* 2025, **12**(3)**:** 886-898.

2. Sadezky A, Muckenhuber H, Grothe H, Niessner R, Pöschl U. Raman microspectroscopy of soot and related carbonaceous materials: Spectral analysis and structural information. *Carbon* 2005, **43**(8)**:** 1731-1742.

3. Ferrari AC, Robertson J. Interpretation of Raman spectra of disordered and amorphous carbon. *Physical Review B* 2000, **61**(20)**:** 14095-14107.

4. Kaniyoor A, Ramaprabhu S. A Raman spectroscopic investigation of graphite oxide derived graphene. *AIP Advances* 2012, **2**(3)**:** 032183.

5. Saurel D, Orayech B, Xiao B, Carriazo D, Li X, Rojo T. From Charge Storage Mechanism to Performance: A Roadmap toward High Specific Energy Sodium-Ion Batteries through Carbon Anode Optimization. *Advanced Energy Materials* 2018, **8**(17)**:** 1703268.

6. Cai X, Hou S, Wu H, Lv Z, Fu Y, Wang D, *et al.* All-carbon electrode-based fiber-shaped dye-sensitized solar cells. *Physical Chemistry Chemical Physics* 2012, **14**(1)**:** 125-130.

7. Antonietti M, Oschatz M. The Concept of "Noble, Heteroatom-Doped Carbons," Their Directed Synthesis by Electronic Band Control of Carbonization, and Applications in Catalysis and Energy Materials. *Advanced Materials* 2018, **30**(21)**:** 1706836.

8. Heidarinejad Z, Dehghani MH, Heidari M, Javedan G, Ali I, Sillanpää M. Methods for preparation and activation of activated carbon: a review. *Environmental Chemistry Letters* 2020, **18**(2)**:** 393-415.

9. Beda A, Vaulot C, Matei Ghimbeu C. Hard carbon porosity revealed by the adsorption of multiple gas probe molecules (N2, Ar, CO2, O2 and H2). *Journal of Materials Chemistry A* 2021, **9**(2)**:** 937-943.

10. Luo W, Bommier C, Jian Z, Li X, Carter R, Vail S, *et al.* Low-surface-area hard carbon anode for na-ion batteries via graphene oxide as a dehydration agent. *ACS Applied Materials & Interfaces* 2015, **7**(4)**:** 2626-2631.

11. Hou B-H, Wang Y-Y, Ning Q-L, Li W-H, Xi X-T, Yang X, *et al.* Self-Supporting, Flexible, Additive-Free, and Scalable Hard Carbon Paper Self-Interwoven by 1D Microbelts: Superb Room/Low-Temperature Sodium Storage and Working Mechanism. *Advanced Materials* 2019, **31**(40)**:** 1903125-1903125.

12. Jafta CJ, Petzold A, Risse S, Clemens D, Wallacher D, Goerigk G, *et al.* Correlating pore size and shape to local disorder in microporous carbon: A combined small angle neutron and X-ray scattering study. *Carbon* 2017, **123:** 440-447.

13. Saurel D, Segalini J, Jauregui M, Pendashteh A, Daffos B, Simon P, *et al.* A SAXS outlook on disordered carbonaceous materials for electrochemical energy storage. *Energy Storage Materials* 2019, **21:** 162-173.

14. Mathis TS, Kurra N, Wang XH, Pinto D, Simon P, Gogotsi Y. Energy Storage Data Reporting in Perspective-Guidelines for Interpreting the Performance of Electrochemical Energy Storage Systems. *Advanced Energy Materials* 2019, **9**(39)**:** 1902007.

15. Horner T, Eren EO, Yılmaz EB, Kim J, Scoppola E, Vasileiadis A, *et al.* Unravelling the Secret of Sulfur Confinement and High Sulfur Utilization in Hybrid Sulfur-Carbons. *Advanced Materials* 2026, **n/a**(n/a)**:** e13346.

16. Dong R, Zheng L, Bai Y, Ni Q, Li Y, Wu F, *et al.* Elucidating the Mechanism of Fast Na Storage Kinetics in Ether Electrolytes for Hard Carbon Anodes. *Advanced Materials* 2021, **33**(36)**:** 2008810.

17. Wu F, Li H, Diemant T, Mullaliu A, Zhang H, Passerini S. Layered Oxide Material as a Highly Stable Na-ion Source and Sink for Investigation of Sodium-ion Battery Materials. *ChemElectroChem* 2024, **11**(3)**:** e202300529.

18. Xu X, Zhou D, Qin X, Lin K, Kang F, Li B*, et al.* A room-temperature sodium–sulfur battery with high capacity and stable cycling performance. *Nature Communications* 2018, **9**(1)**:** 3870.

19. Li K, Zhang J, Lin D, Wang D-W, Li B, Lv W*, et al.* Evolution of the electrochemical interface in sodium ion batteries with ether electrolytes. *Nature Communications* 2019, **10**(1)**:** 725.

20. Fondard J, Irisarri E, Courrèges C, Palacin MR, Ponrouch A, Dedryvère R. SEI Composition on Hard Carbon in Na-Ion Batteries After Long Cycling: Influence of Salts (NaPF6, NaTFSI) and Additives (FEC, DMCF). *Journal of the Electrochemical Society* 2020, **167**(7)**:** 070526.

21. Xu Z-L, Lim K, Park K-Y, Yoon G, Seong WM, Kang K. Engineering Solid Electrolyte Interphase for Pseudocapacitive Anatase TiO2 Anodes in Sodium-Ion Batteries. *Advanced Functional Materials* 2018, **28**(29)**:** 1802099.

22. Itagaki M, Honda K, Hoshi Y, Shitanda I. In-situ EIS to determine impedance spectra of lithium-ion rechargeable batteries during charge and discharge cycle. *Journal of Electroanalytical Chemistry* 2015, **737:** 78-84.

23. Linsenmann F, Pritzl D, Gasteiger HA. Comparing the Lithiation and Sodiation of a Hard Carbon Anode Using In Situ Impedance Spectroscopy. *Journal of the Electrochemical Society* 2021, **168**(1)**:** 010506.

24. Hua N, Chen H, Dong G. Deep transfer learning-enabled battery health prognosis using impedance spectrum data. *Journal of Energy Storage* 2025, **132:** 117855.

25. Zhao Y, Kücher S, Jossen A. Investigation of the diffusion phenomena in lithium-ion batteries with distribution of relaxation times. *Electrochimica Acta* 2022, **432:** 141174.

26. Semerukhin DY, Kubarkov AV, Sergeyev VG, Semenikhin OA, Antipov EV. Analysis of the Distribution of Relaxation Times (DRT) Responses of Li-Ion Cells as a Function of Their Preparation Conditions. *Electrochimica Acta* 2024, **486:** 144092.

27. Schutjajew K, Tichter T, Schneider J, Antonietti M, Roth C, Oschatz M. Insights into the sodiation mechanism of hard carbon-like materials from electrochemical impedance spectroscopy. *Physical Chemistry Chemical Physics* 2021, **23**(19)**:** 11488-11500.

28. Iurilli P, Brivio C, Wood V. Detection of Lithium-Ion Cells' Degradation through Deconvolution of Electrochemical Impedance Spectroscopy with Distribution of Relaxation Time. *Energy Technology* 2022, **10**(10)**:** 2200547.

29. Eren EO, Senokos E, Scoppola E, Song Z, Antonietti M, Giusto P. An enhanced three-stage model for sodium storage in hard carbons. *Energy & Environmental Science* 2025, **18**(16)**:** 7859-7868.

30. Weppner W, Huggins RA. Determination of the Kinetic Parameters of Mixed-Conducting Electrodes and Application to the System Li3Sb. *Journal of the Electrochemical Society* 1977, **124**(10)**:** 1569.

31. Delacourt C, Ati M, Tarascon JM. Measurement of Lithium Diffusion Coefficient in LiyFeSO4F. *Journal of the Electrochemical Society* 2011, **158**(6)**:** A741.

32. Horner JS, Whang G, Ashby DS, Kolesnichenko IV, Lambert TN, Dunn BS*, et al.* Electrochemical Modeling of GITT Measurements for Improved Solid-State Diffusion Coefficient Evaluation. *ACS Applied Energy Materials* 2021, **4**(10)**:** 11460-11469.

33. Jian Z, Xing Z, Bommier C, Li Z, Ji X. Hard Carbon Microspheres: Potassium-Ion Anode Versus Sodium-Ion Anode. *Advanced Energy Materials* 2016, **6**(3)**:** 1501874.

34. Feng X, Li Y, Li Y, Liu M, Zheng L, Gong Y*, et al.* Unlocking the local structure of hard carbon to grasp sodium-ion diffusion behavior for advanced sodium-ion batteries. *Energy & Environmental Science* 2024, **17**(4)**:** 1387-1396.

35. Stratford JM, Kleppe AK, Keeble DS, Chater PA, Meysami SS, Wright CJ*, et al.* Correlating Local Structure and Sodium Storage in Hard Carbon Anodes: Insights from Pair Distribution Function Analysis and Solid-State NMR. *Journal of the American Chemical Society* 2021, **143**(35)**:** 14274-14286.

36. Claye A, Fischer JE. Short-range order in disordered carbons: where does the Li go? *Electrochimica Acta* 1999, **45**(1)**:** 107-120.

37. Au H, Alptekin H, Jensen ACS, Olsson E, O'Keefe CA, Smith T*, et al.* A revised mechanistic model for sodium insertion in hard carbons. *Energy & Environmental Science* 2020, **13**(10)**:** 3469-3479.

38. Chen D, Zhang W, Luo K, Song Y, Zhong Y, Liu Y*, et al.* Hard carbon for sodium storage: mechanism and optimization strategies toward commercialization. *Energy & Environmental Science* 2021, **14**(4)**:** 2244-2262.

39. Yuan R, Guo Y, Gurgan I, Siddique N, Li Y-S, Jang S*, et al.* Raman spectroscopy analysis of disordered and amorphous carbon materials: A review of empirical correlations. *Carbon* 2025, **238:** 120214.

40. Merlen A, Buijnsters JG, Pardanaud C. A Guide to and Review of the Use of Multiwavelength Raman Spectroscopy for Characterizing Defective Aromatic Carbon Solids: from Graphene to Amorphous Carbons. *Coatings*; 2017.

41. Shirodkar SN, Waghmare UV. Electronic and vibrational signatures of Stone-Wales defects in graphene: First-principles analysis. *Physical Review B* 2012, **86**(16)**:** 165401.

42. Conder J, Villevieille C. How reliable is the Na metal as a counter electrode in Na-ion half cells? *Chemical Communications* 2019, **55**(9)**:** 1275-1278.

43. Kamiyama A, Kubota K, Igarashi D, Youn Y, Tateyama Y, Ando H*, et al.* MgO-Template Synthesis of Extremely High Capacity Hard Carbon for Na-Ion Battery. *Angewandte Chemie International Edition* 2021, **60**(10)**:** 5114-5120.

44. Peng J, Wang H, Shi X, Fan HJ. Ultrahigh Plateau-Capacity Sodium Storage by Plugging Open Pores. *Advanced Materials* 2024, **n/a**(n/a)**:** 2410326.

45. Li Q, Liu X, Tao Y, Huang J, Zhang J, Yang C*, et al.* Sieving carbons promise practical anodes with extensible low-potential plateaus for sodium batteries. *National Science Review* 2022, **9**(8)**:** nwac084.

46. Fan C, Zhang R, Luo X, Hu Z, Zhou W, Zhang W*, et al.* Epoxy phenol novolac resin: A novel precursor to construct high performance hard carbon anode toward enhanced sodium-ion batteries. *Carbon* 2023, **205:** 353-364.

47. Zhao X, Shi P, Wang H, Meng Q, Qi X, Ai G*, et al.* Unlocking plateau capacity with versatile precursor crosslinking for carbon anodes in Na-ion batteries. *Energy Storage Materials* 2024, **70:** 103543.

48. Qiu C, Li A, Qiu D, Wu Y, Jiang Z, Zhang J*, et al.* One-Step Construction of Closed Pores Enabling High Plateau Capacity Hard Carbon Anodes for Sodium-Ion Batteries: Closed-Pore Formation and Energy Storage Mechanisms. *ACS Nano* 2024, **18**(18)**:** 11941-11954.

49. Zheng Z, Hu S, Yin W, Peng J, Wang R, Jin J*, et al.* CO2-Etching Creates Abundant Closed Pores in Hard Carbon for High-Plateau-Capacity Sodium Storage. *Advanced Energy Materials* 2024, **14**(3)**:** 2303064.

50. Zhao C, Wang Q, Lu Y, Li B, Chen L, Hu Y-S. High-temperature treatment induced carbon anode with ultrahigh Na storage capacity at low-voltage plateau. *Science Bulletin* 2018, **63**(17)**:** 1125-1129.

51. Liu J, You Y, Huang L, Zheng Q, Sun Z, Fang K*, et al.* Precisely Tunable Instantaneous Carbon Rearrangement Enables Low-Working-Potential Hard Carbon Toward Sodium-Ion Batteries with Enhanced Energy Density. *Advanced Materials* 2024, **36**(44)**:** 2407369.

52. Liu L, Gu Y, Li J, Bashir S, Kasi R, Fang Y*, et al.* A Stage-Wise Plateau-Sodiation Mechanism Enabled by Ultramicropores in the Hard Carbon Anode for Sodium Storage. *Advanced Energy Materials* 2025, **n/a**(n/a)**:** e04853.

53. Chen X, Sawut N, Chen K, Li H, Zhang J, Wang Z*, et al.* Filling carbon: a microstructure-engineered hard carbon for efficient alkali metal ion storage. *Energy & Environmental Science* 2023, **16**(9)**:** 4041-4053.

54. Meng Q, Lu Y, Ding F, Zhang Q, Chen L, Hu Y-S. Tuning the Closed Pore Structure of Hard Carbons with the Highest Na Storage Capacity. *ACS Energy Letters* 2019, **4**(11)**:** 2608-2612.

55. Kamiyama A, Kubota K, Nakano T, Fujimura S, Shiraishi S, Tsukada H*, et al.* High-Capacity Hard Carbon Synthesized from Macroporous Phenolic Resin for Sodium-Ion and Potassium-Ion Battery. *ACS Applied Energy Materials* 2020, **3**(1)**:** 135-140.
